# Correlated quantum shift vector of particle-hole excitations

**DOI:** 10.1038/s41467-026-72878-8

**Published:** 2026-05-15

**Authors:** Xu Yang, Ajit Srivastava, Justin C. W. Song

**Affiliations:** 1 Division of Physics and Applied Physics, School of Physical and Mathematical Sciences, Nanyang Technological University, Singapore, Singapore; 2 Department of Quantum Matter Physics, University of Geneva, Geneva, Switzerland

**Keywords:** Nonlinear optics, Topological matter, Two-dimensional materials

## Abstract

Excitons are a prime example of how electron interactions affect optical response and excitation. For example, electron-hole interactions produce a bound excitonic spectrum. Here we show that, beyond its spectra, the bound nature of an exciton’s electron-hole pair produces a correlated quantum geometry: excitonic excitations possess a quantum shift vector that is independent of light polarization. We find this counterintuitive behavior has dramatic consequences for geometric response: e.g., in noncentrosymmetric but non-polar materials, vertical excitonic transitions possess vanishing shift vector zeroing their shift photocurrent; this contrasts with finite and strongly light polarization dependent shift vectors for non-interacting delocalized particle-hole excitations. This dichotomy makes shift vector a sharp diagnostic of the pair localization properties of particle-hole excitations and demonstrates the non-perturbative effects of electron interactions in excited state quantum geometric response.

## Abstract (web summary)

Both excitons and free particle-hole excitations comprise electrons and holes in the conduction and valence bands. Here, the authors show that excitonic electron-hole interactions drive a correlated excitonic quantum geometry that is distinct from free particle-hole excitations.

## Introduction

Particle-hole excitations are key processes in optoelectronics. Often characterized by their excitation spectra, the quantum geometry of (optical) transition dipole moments is now recognized to play an outsized role in dynamical response^1–3^: e.g., the quantum metric determines the spectral weight^1,4,5^ and the quantum shift vector^3,6^, which describes light-induced changes to electric polarization, produces bulk photocurrents even in the absence of p-n junctions^1–3,6–13^.

Weakly interacting particle-hole excitations possess a quantum geometry that mirrors the wavefunction winding of single-particle Bloch states^1,2^. This correspondence renders the opto-electronic response a powerful probe of the Bloch band geometry of quantum materials^1,14^. For example, weakly interacting particle-hole excitations possess shift vectors that are strongly dependent on light polarization^8,9^; their associated shift current tracks the Hermitian connection of resonant optical transitions^1^. Excitonic particle-hole excitations, on the other hand, are fundamentally different. Even as each exciton is mobile and moves freely as a single unit, its electron and hole are bound strongly together by interactions, producing a pair envelope wavefunction that is real-space localized^15^.

Here, we argue that such electron-hole interactions drive a correlated excitonic quantum geometry distinct from free particle-hole excitations. In particular, we find that the quantum shift vector for excitonic optical excitations is independent of the light polarization. This counterintuitive result sharply contrasts with the strongly light polarization-dependent shift vector for free particle-hole excitations. It is especially striking given that both excitonic and delocalized particle-hole excitations can possess similar transition dipole moments and quantum weight. We trace this delineation to the interaction-induced relative coordinate localization of the electron-hole pair envelope wavefunction for bound excitons; free particle-hole pairs, in contrast, possess a delocalized pair envelope. We find that pair localization differentiates the way geometric phases accumulate, enabling the quantum shift vector to act as a “geometric ruler” of pair localization. These correlated features readily dominate the photocurrent response for a range of technologically relevant optoelectronic materials^10,11^. A dramatic example is vertical transitions, where we find the excitonic quantum shift vector vanishes in noncentrosymmetric but non-polar point groups, rendering the shift photocurrent zero for excitonic transitions. In sharp contrast, shift photocurrents for delocalized free particle-hole excitations are generically allowed when inversion symmetry is broken^8,9^. Together, these underscore the importance of electron interactions in the optical response and quantum geometry of excited quantum matter.

## Results

### Flux threading and particle-hole excitations

We begin by examining the structure of excitations of an insulating ground state, $| GS\rangle$. For clarity, we focus on $| GS\rangle$ with filled valence bands *v* and empty conduction bands *c*. Generic particle-hole excitations on top of $| GS\rangle$ with a conserved total momentum ***Q*** take the form:

1 $${| {\Phi }_{{{{\boldsymbol{Q}}}}}\rangle }_{p-h}=\frac{1}{\sqrt{N}}{\sum }_{{{{\bf{r}}}},{{{{\bf{R}}}}}_{cm}}{\psi }_{{{{\boldsymbol{Q}}}}}({{{\bf{r}}}}){e}^{i{{{\boldsymbol{Q}}}}\cdot {{{{\bf{R}}}}}_{cm}}| {{{{\bf{R}}}}}_{cm},{{{\bf{r}}}}\rangle,$$

where *N* is the number of unit-cells, $| {{{{\bf{R}}}}}_{cm},{{{\bf{r}}}}\rangle \equiv {c}_{c,{{{{\bf{R}}}}}_{e}}^{{{\dagger}} }{c}_{v,{{{{\bf{R}}}}}_{h}}| GS\rangle$ denotes a particle-hole pair with a center-of-mass coordinate **R**_cm_ = (**R**_*e*_ + **R**_*h*_)/2 and relative coordinate **r** = **R**_*e*_ − **R**_*h*_. Here ${c}_{c(v),{{{\bf{R}}}}}^{{{\dagger}} }$ is the creation operator of a Wannier state with wavefunction *w*_*c*(*v*),**R**_(**x**)^16^ in the conduction (valence) bands and *ψ*_***Q***_(**r**) is the envelope function of the particle-hole excitation. In what follows, we will employ localized Wannier functions^16,17^.

Momentum ***Q*** is a good quantum number describing the free motion of the mobile center of mass of the particle-hole pair. In contrast, *ψ*_***Q***_(**r**) encodes the internal structure of the particle-hole excitation characterizing its interaction with electromagnetic fields. By projecting onto the particle-hole basis in Eq. (1), *ψ*_***Q***_(**r**) can be directly obtained via the Bethe-Salpeter equation (BSE): ${\sum }_{{{{{\bf{r}}}}}^{{\prime} }}{{{{\mathcal{H}}}}}_{{{{\boldsymbol{Q}}}}}({{{\bf{r}}}},{{{{\bf{r}}}}}^{{\prime} }){\psi }_{{{{\boldsymbol{Q}}}}}({{{{\bf{r}}}}}^{{\prime} })=E{\psi }_{{{{\boldsymbol{Q}}}}}({{{\bf{r}}}})$ with^15,18,19^

2 $${{{{\mathcal{H}}}}}_{{{{\boldsymbol{Q}}}}}={\delta }_{{{{\bf{r}}}},{{{{\bf{r}}}}}^{{\prime} }}[{\epsilon }_{c}(\widehat{{{{\bf{p}}}}}+{{{\boldsymbol{Q}}}}/2)-{\epsilon }_{v}(\widehat{{{{\bf{p}}}}}-{{{\boldsymbol{Q}}}}/2)]+{{{{\mathcal{V}}}}}_{{{{\boldsymbol{Q}}}}}({{{\bf{r}}}},{{{{\bf{r}}}}}^{{\prime} }),$$

where $\widehat{{{{\bf{p}}}}}\equiv -i{\partial }_{{{{\bf{r}}}}}$ and ${{{{\mathcal{V}}}}}_{{{{\boldsymbol{Q}}}}}({{{\bf{r}}}},{{{{\bf{r}}}}}^{{\prime} })$ is the particle-hole interaction; see Supplementary Information (SI), Sec. I for discussion. Here *ϵ*_*c*,*v*_ are the *c*, *v* band energies.

In what follows, we focus on a real-space BSE to expose the critical role pair localization plays in the correlated quantum geometry of excitons. Indeed, Eq. (2) readily displays how the scattering (free particle-hole excitations) and bound (exciton) states are delineated by their pair localization properties in the relative (particle-hole) coordinate. When the excitation energies are above the optical gap, $\Delta ({{{\boldsymbol{Q}}}})={\min }_{{{{\bf{p}}}}}[{\epsilon }_{c}({{{\bf{p}}}}+{{{\boldsymbol{Q}}}}/2)-{\epsilon }_{v}({{{\bf{p}}}}-{{{\boldsymbol{Q}}}}/2)]$, Eq. (2) can be naturally solved with the asymptotic plane wave scattering solutions for the envelope function (*e*^*i***p**⋅**r**^ for large *r*) with energies *ϵ*_*c*_(**p** + ***Q***/2) − *ϵ*_*v*_(**p** − ***Q***/2). Such scattering states are completely delocalized, corresponding to unbound particle-hole pairs. In contrast, when the energy *E* lies below the optical gap *Δ*(***Q***), the value of **p** becomes imaginary, leading to an exponentially decaying envelope function: these are the bound exciton states.

As we now argue, the pair localization properties of the particle-hole excitation directly affect their quantum geometry. To illustrate this, we examine the behavior of the envelope function under insertion of uniform flux ***κ*** (units inverse length) in a periodic system size *L*. Flux-insertion has been used in the literature to describe the many-body Berry curvature and quantum metric of electronic systems^20–24^. Operationally, this corresponds to imposing periodic boundary conditions along one direction to form a cylinder and threading a static gauge flux *ϕ* through the hole (see **SI**, Fig. S1). This induces a uniform static vector potential ***κ*** along the periodic direction, with ∮***κ*** ⋅ *d***l** = *ϕ*. A particle moving in such a background accumulates a quantum-mechanical phase, so that the flux-inserted electronic Wannier function is modified by a phase depending on the electron’s displacement from its Wannier center^20,25^:

3 $${w}_{{{{\boldsymbol{R}}}}}^{{{{\boldsymbol{\kappa }}}}}({{{\bf{x}}}})={e}^{-i{{{\boldsymbol{\kappa }}}}\cdot ({{{\bf{x}}}}-{{{\boldsymbol{R}}}})}{w}_{{{{\boldsymbol{R}}}}}({{{\bf{x}}}}).$$

Crucially, since ***κ*** couples to the charge of the particle, the accumulated phase has opposite signs for electrons and holes. As a result, for particle-hole excitations, the co-motion of an electron and a hole incurs no net phase. Instead, ***κ*** couples to the relative coordinate ***r***. Accordingly, the flux-inserted BSE matrix elements acquire a phase factor (see **SI**, Sec. II):

4 $${{{{\mathcal{H}}}}}_{{{{\boldsymbol{Q}}}}}^{{{{\boldsymbol{\kappa }}}}}({{{\bf{r}}}},{{{{\bf{r}}}}}^{{\prime} })={e}^{-i{{{\boldsymbol{\kappa }}}}\cdot ({{{\bf{r}}}}-{{{{\bf{r}}}}}^{{\prime} })}{{{{\mathcal{H}}}}}_{{{{\boldsymbol{Q}}}}}({{{\bf{r}}}},{{{{\bf{r}}}}}^{{\prime} }),$$

where ${{{{\mathcal{H}}}}}_{{{{\boldsymbol{Q}}}}}^{{{{\boldsymbol{\kappa }}}}}$ is the flux-inserted BSE. Note that flux accumulation through the relative coordinate arises from opposite signs of particle and hole charge, rendering the envelope function key to understanding its response. In contrast, for superconductors, flux couples to the center of mass coordinate of Cooper pairs^26^.

In what follows, we work with periodic boundary conditions. The envelope function satisfies *ψ*_***Q***_(**r** + *L****e***_*κ*_) = *ψ*_***Q***_(**r**) with ***e***_*κ*_ along the periodic direction. Notice that **r** defines relative coordinates denoting **R**_*e*_ − **R**_*h*_. Consequently, it is useful to distinguish two regions. When **r** is near $| {{{\bf{r}}}}\cdot {{{{\boldsymbol{e}}}}}_{\kappa }|=0\,(mod\,L)$, the electron and hole remain close to each other. In contrast, when **r** is close to $| {{{\bf{r}}}}\cdot {{{{\boldsymbol{e}}}}}_{\kappa }|=L/2\,(mod\,L)$, they are maximally separated; we refer to this as the boundary region. As we will see below, localization properties are most apparent at the boundary region.

The bound and scattering solutions of Eq. (2) change differently under flux insertion. The exponential pair localization of the exciton envelope function *ψ*_***Q***_(**r**) severely constrains its transformation properties. To see this, we apply a gauge transformation to gauge away the phase accumulated by flux insertion in Eq. (4)^20,23^ via the ansatz: ${\widetilde{\psi }}_{{{{\boldsymbol{Q}}}}}^{\kappa }({{{\bf{r}}}})={e}^{-i{{{\boldsymbol{\kappa }}}}\cdot {{{\bf{r}}}}}{\psi }_{{{{\boldsymbol{Q}}}}}({{{\bf{r}}}})$.

**Fig. 1:**
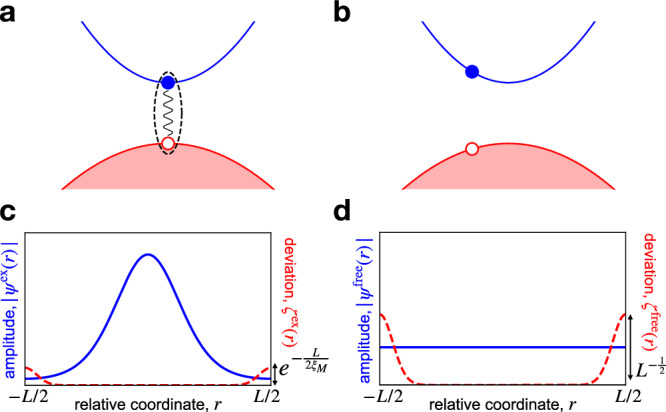
Localized vs. delocalized particle-hole excitations. **a** Both excitons and **b** free particle-hole excitations consist of electrons (solid dots) in the conduction band (blue) and holes (open circles) in the valence band (red). The former are localized by strong particle-hole interactions, while the latter are delocalized. **c** Schematic of amplitude of exciton envelope wavefunction (blue) and flux-threading ansatz deviation *ζ*^ex^(**r**) (red) in a one-dimensional finite periodic system of size *L*. Because the exciton is bound, the deviation is exponentially suppressed at the boundaries. **d** Schematic of the amplitude of the delocalized envelope of a free particle-hole pair (blue) and “ansatz deviation” *ζ*^free^(**r**) (red). For delocalized states, the deviation is large and comparable to that of the envelope function.

Notice that ${\widetilde{\psi }}_{{{{\boldsymbol{Q}}}}}^{\kappa }({{{\bf{r}}}})$ is not the exact solution of Eq. (4). While it closely tracks the actual flux-inserted envelope wavefunction [solution of Eq. (4)] in the bulk, it deviates close to the boundary region due to the phase factor *e*^−*i****κ***⋅**r**^ [see Fig. 1c,d]. This deviation can be quantified by direct substitution into Eq. (4)(see evaluation in “Methods”):

5 $$\zeta ({{{\boldsymbol{r}}}})=\left| {\sum }_{{{{{\bf{r}}}}}^{{\prime} }}{{{{\mathcal{H}}}}}_{{{{\boldsymbol{Q}}}}}^{{{{\boldsymbol{\kappa }}}}}({{{\bf{r}}}},{{{{\bf{r}}}}}^{{\prime} }){\widetilde{\psi }}_{{{{\boldsymbol{Q}}}}}^{{{{\boldsymbol{\kappa }}}}}({{{{\bf{r}}}}}^{{\prime} })-E{\widetilde{\psi }}_{{{{\boldsymbol{Q}}}}}^{{{{\boldsymbol{\kappa }}}}}({{{\bf{r}}}})\right| \le {{{\mathcal{C}}}}{e}^{-\frac{L}{2{\xi }_{M}}},$$

where we have substituted the exciton envelope wavefunction in the last inequality: this exponentially suppresses the deviation [see Fig. 1c]. Here ${{{\mathcal{C}}}}$ is a constant independent of *L* and *ξ*_*M*_ is ${\xi }_{M}\equiv \max ({\xi }_{W},\xi )$, with *ξ*_*W*_ and *ξ* is the extent of the exponentially decaying Wannier function and exciton envelope function, respectively.

**Fig. 2:**
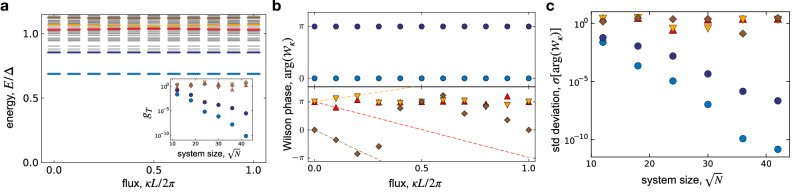
Numerical flux threading properties of particle-hole excitations. **a** Energy spectrum versus flux *κ* threaded along $\widehat{x}$ direction: exciton (light blue, dark blue) energy remains constant with flux, while delocalized particle-hole excitations vary significantly with *κ* (gray, colored). (inset) Thouless number *g*_*T*_ ≡ ∣*E*^*κL*=0^ − *E*^*κL*=*π*^∣/(*Δ*/*N*) quantifies sensitivity to flux insertion (normalized by level spacing *Δ*/*N*): *g*_*T*_ exponentially decays for excitons (light blue dots, dark blue dots) but remains ${{{\mathcal{O}}}}(1)$ for free particle-hole pairs even at large *N* (red upward triangle, orange downward triangle, brown diamond). **b** Argument of Wilson loop operator (Eq. (9)) with ${\widehat{V}}_{1}=(\sqrt{3}{\widehat{v}}_{x}+{\widehat{v}}_{y})/2$ and ${\widehat{V}}_{2}=({\widehat{v}}_{x}-\sqrt{3}{\widehat{v}}_{y})/2$ versus *κ* with $\widehat{v}$ the velocity operator. For excitons (top panel, light blue and dark blue dots), $\arg ({{{{\mathcal{W}}}}}_{{{{\boldsymbol{\kappa }}}}})$ is flat demonstrating insensitivity to $\widehat{V}$; for delocalized particle-hole excitations (bottom panel, red upward triangles, orange downward triangles, brown diamonds) $\arg ({{{{\mathcal{W}}}}}_{{{{\boldsymbol{\kappa }}}}})$ shows strong ***κ*** dependence. Dashed lines indicate the derivative of $\arg ({{{{\mathcal{W}}}}}_{{{{\boldsymbol{\kappa }}}}})$ at ***κ*** = 0. **c** Standard deviation of $\arg ({{{{\mathcal{W}}}}}_{{{{\boldsymbol{\kappa }}}}})$ vs. $\sqrt{N}$, showing exponential decay $\sim {e}^{-L/{\xi }_{M}}$ for excitons (light blue and dark blue dots), and ${{{\mathcal{O}}}}(1)$ behavior for delocalized particle-hole states (red upward triangles, orange downward triangles, brown diamonds). In all plots, Eq. (4) was numerically solved on an *N* cell honeycomb lattice with staggered sublattice potential, system size $L=\sqrt{N}a$, and *a* the lattice constant, see “Methods” and **SI**, Sec. VII for parameter values and numerical details. Colors and symbols consistently label the same states across all panels.

For excitons, *ζ*^ex^(***r***) diminishes exponentially. Accordingly, we conclude that the bound excitonic state reads:

6 $${[{\psi }_{{{{\boldsymbol{Q}}}}}^{{{{\boldsymbol{\kappa }}}}}]}^{ex}({{{\bf{r}}}})={e}^{-i{{{\boldsymbol{\kappa }}}}\cdot {{{\bf{r}}}}}{[{\psi }_{{{{\boldsymbol{Q}}}}}]}^{ex}({{{\bf{r}}}})+{{{\mathcal{O}}}}(\exp [-L/(2{\xi }_{M})]),$$

with an energy that is insensitive to flux threading ${E}^{ex}({{{\boldsymbol{\kappa }}}})={E}^{ex}({{{\boldsymbol{0}}}})+{{{\mathcal{O}}}}(\exp [-L/{\xi }_{M}])$. Indeed, performing a scaling analysis in *L* with numerical solutions of Eq. (4), we find the exciton’s energy variation with ***κ*** is exponentially suppressed, Fig. 2(a) and inset (light blue and dark blue dots). This mirrors what is expected of an insulator^20^. As we will see below, this insensitivity to flux threading plays a key role in the polarization properties of excitonic excitations.

In contrast, flux threading for delocalized free particle-hole excitations cannot be gauged away. While the deviation *ζ*^free^(***r***) for the ansatz ${e}^{-i{{{\boldsymbol{\kappa }}}}\cdot {{{\bf{r}}}}}{\psi }_{{{{\boldsymbol{Q}}}}}^{free}({{{\bf{r}}}})$ vanishes in the bulk, it becomes significant near the boundary due to the discontinuous phase factor *e*^−*i****κ***⋅**r**^ across the boundary and the finite envelope amplitude there. The resulting violation is comparable in magnitude to the envelope function itself, see Fig. 1d. This indicates that the delocalized states change in an essential way (not just via a phase factor) that is sensitive to the details of how ***κ*** is inserted. Indeed, a numerical solution of Eq. (4) for delocalized particle-hole excitations produces energy shifts comparable to the level spacing as ***κ*** is varied, characteristic of a strong sensitivity to ***κ***, Fig. 2(a) and inset.

### Shift vector probes pair localization of particle-hole excitations

We now proceed to analyze the electric polarization change in a many-electron system produced by a particle-hole excitation. Such light induced changes are captured by a many-body quantum shift vector^6^:

7 $${{{\boldsymbol{{\mathscr{R}}}}}}_{0\to n}\equiv {{{\mathrm{lim}}}}_{{{{\boldsymbol{\kappa }}}}\to {{{\bf{0}}}}}\left\{\delta {{{\boldsymbol{{\mathscr{A}}}}}}_{{{{\boldsymbol{\kappa }}}}}^{(0\to n)}+{\nabla }_{{{{\boldsymbol{\kappa }}}}}\arg \left[\langle {\Phi }_{0}({{{\boldsymbol{\kappa }}}})| \widehat{V}({{{\boldsymbol{\kappa }}}})| {\Phi }_{n}({{{\boldsymbol{\kappa }}}})\rangle \right]\right\},$$

where $| {\Phi }_{n,0}({{{\boldsymbol{\kappa }}}})\rangle$ are the many-body excited and ground states, the many-body Berry connection difference reads $\delta {{{\boldsymbol{{\mathscr{A}}}}}}_{{{{\boldsymbol{\kappa }}}}}^{(0\to n)}=i\langle {\Phi }_{n}({{{\boldsymbol{\kappa }}}})| {\nabla }_{{{{\boldsymbol{\kappa }}}}}| {\Phi }_{n}({{{\boldsymbol{\kappa }}}})\rangle -i\langle {\Phi }_{0}({{{\boldsymbol{\kappa }}}})| {\nabla }_{{{{\boldsymbol{\kappa }}}}}| {\Phi }_{0}({{{\boldsymbol{\kappa }}}})\rangle$ for the excited (*n*) and (0) ground states, and $\widehat{V}$ is the light-matter interaction. This flux-defined quantum shift vector^6^ allows us to isolate the effect of real-space pair localization and address both free and excitonic excitations within the same framework.

Observe that all terms of Eq. (7) are required to ensure its gauge invariance: the last term of Eq. (7) compensates gauge transformations $| \Phi \rangle \to {e}^{i\theta ({{{\boldsymbol{\kappa }}}})}| \Phi \rangle$. As a result, light polarization dependence encoded in $\widehat{V}$ is often regarded as an essential feature of light-induced electric polarization changes^7,27^; indeed, shift vectors often change sign with light polarization^8,9^.

We now show that the converse is true for excitonic excitations: polarization changes from an insulating many-body ground state to an excited excitonic state are insensitive to $\widehat{V}$. To see this, we consider two light matter interactions ${\widehat{V}}_{1}$ and ${\widehat{V}}_{2}$ e.g., from two different polarizations of light; both ${\widehat{V}}_{1}$ and ${\widehat{V}}_{2}$ produce an excitation from the ground to exciton state. The difference between their light-induced polarization changes $\delta {{\boldsymbol{{\mathscr{R}}}}}={{{\boldsymbol{{\mathscr{R}}}}}}_{0\to ex}^{{V}_{1}}-{{{\boldsymbol{{\mathscr{R}}}}}}_{0\to ex}^{{V}_{2}}={\nabla }_{{{{\boldsymbol{\kappa }}}}}\arg {{{{\mathcal{W}}}}}_{{{{\boldsymbol{\kappa }}}}}$, where the Wilson loop ${{{{\mathcal{W}}}}}_{{{{\boldsymbol{\kappa }}}}}$^28^ is ${{{{\mathcal{W}}}}}_{{{{\boldsymbol{\kappa }}}}}={[{V}_{1}^{{{{\boldsymbol{\kappa }}}}}]}_{0\to ex}{[{V}_{2}^{{{{\boldsymbol{\kappa }}}}}]}_{ex\to 0}$, with a general transition matrix element represented by ${[{V}^{{{{\boldsymbol{\kappa }}}}}]}_{n\to m}=\langle {\Phi }_{n}({{{\boldsymbol{\kappa }}}})| \widehat{V}({{{\boldsymbol{\kappa }}}})| {\Phi }_{m}({{{\boldsymbol{\kappa }}}})\rangle$. Note that the transition matrix element between the ground state and the exciton state can be directly evaluated via Eq. (1):

8 $${\left[{V}^{{{{\boldsymbol{\kappa }}}}}\right]}_{0\to ex}={\sum }_{{{{{\bf{R}}}}}_{e},{{{{\bf{R}}}}}_{h}}\frac{{e}^{i{{{\boldsymbol{Q}}}}\cdot {{{{\bf{R}}}}}_{cm}}}{\sqrt{N}}{v}_{{{{{\bf{R}}}}}_{h},{{{{\bf{R}}}}}_{e}}^{{{{\boldsymbol{\kappa }}}}}{\psi }_{{{{\boldsymbol{Q}}}}}^{{{{\boldsymbol{\kappa }}}}}({{{{\bf{R}}}}}_{e}-{{{{\bf{R}}}}}_{h}),$$

where ${v}_{{{{{\bf{R}}}}}_{h},{{{{\bf{R}}}}}_{e}}^{{{{\boldsymbol{\kappa }}}}}=\langle {w}_{{{{{\bf{R}}}}}_{h}}^{{{{\boldsymbol{\kappa }}}}}| \widehat{V}| {w}_{{{{{\bf{R}}}}}_{e}}^{{{{\boldsymbol{\kappa }}}}}\rangle$ is the transition amplitude in the Wannier basis. To analyze Eq. (8), we first note that by directly applying Eq. (3), the flux-inserted transition amplitude is related to that without flux via the relation: ${v}_{{{{{\bf{R}}}}}_{h},{{{{\bf{R}}}}}_{e}}^{{{{\boldsymbol{\kappa }}}}}={e}^{-i{{{\boldsymbol{\kappa }}}}\cdot ({{{{\bf{R}}}}}_{h}-{{{{\bf{R}}}}}_{e})}{v}_{{{{{\bf{R}}}}}_{h},{{{{\bf{R}}}}}_{e}}$. Secondly, the flux-inserted envelope function ${\psi }_{{{{\boldsymbol{Q}}}}}^{{{{\boldsymbol{\kappa }}}}}({{{{\bf{R}}}}}_{e}-{{{{\bf{R}}}}}_{h})$ picks up a phase factor ${e}^{-i{{{\boldsymbol{\kappa }}}}\cdot ({{{{\bf{R}}}}}_{e}-{{{{\bf{R}}}}}_{h})}$ according to Eq. (6). Combining these, we find that the two ***κ***-dependent phases compensate in the transition matrix element in Eq. (8). As a result, evaluating $\arg ({{{{\mathcal{W}}}}}_{\kappa })$ we find:

9 $$| \delta {{\boldsymbol{{\mathscr{R}}}}}|=| {\nabla }_{{{{\boldsymbol{\kappa }}}}}\arg {{{{\mathcal{W}}}}}_{{{{\boldsymbol{\kappa }}}}}| < {{{\mathcal{D}}}}{e}^{-L/{\xi }_{M}},$$

where ${{{\mathcal{D}}}}$ is a constant independent of *L* (see also **SI**, Sec. III).

Exponential suppression of $\delta {{\boldsymbol{{\mathscr{R}}}}}$ means that light-induced changes to electric polarization from a ground state to an excitonic state are exponentially insensitive to $\widehat{V}$; it loses all dependence on $\widehat{V}$ in the thermodynamic limit *L* → *∞*.

The behavior of the Wilson loop ${{{{\mathcal{W}}}}}_{{{{\boldsymbol{\kappa }}}}}$ as a function of ***κ*** for two different incident light polarizations is shown in Fig. 2(b). For excitonic states (upper panel), the ***κ*** dependence is exponentially suppressed, indicating that the shift vector is insensitive to light polarization. This stands in stark contrast to scattering states (lower panel), where ${{{{\mathcal{W}}}}}_{{{{\boldsymbol{\kappa }}}}}$ varies significantly with ***κ***: this reflects a strong light polarization dependence of the free particle-hole excitation shift vector^8,9^. This sensitivity arises from the strong dependence of delocalized wavefunctions on flux insertion^20,23^. As a result, we arrive at one of our key results: the shift vector probes the pair localization properties of particle-hole excitations.

### Symmetry and intrinsic excitonic transition shift vector

Note that while we have focused on light matter interaction $\widehat{V}$, Eq. (9) applies generally for particle number conserving interactions. Indeed, by explicitly computing the polarization change between two excitonic states (e.g., transitions induced by disorder or phonons), we find: ${{{\boldsymbol{{\mathscr{R}}}}}}_{{ex}_{1}\to {ex}_{2}}\doteq {{{\boldsymbol{{\mathscr{R}}}}}}_{0\to {ex}_{2}}-{{{\boldsymbol{{\mathscr{R}}}}}}_{0\to {ex}_{1}}$ where ex_1,2_ denote distinct excitonic states (e.g., with different center of mass momentum ***Q***). Here, we use  ≐ to denote equality up to an exponentially suppressed term in system size *L*^20^; it becomes an equality in the thermodynamic limit.

As a result, shift vectors for excitonic transitions are intrinsic: electric polarization changes induced by light only depend on the initial and final state. For non-degenerate excitonic states, this produces a compact excitonic transition shift vector in real-space:

10 $${{{\boldsymbol{{\mathscr{R}}}}}}_{0\to ex}\doteq {\sum }_{{{{\bf{r}}}},{{{{\bf{r}}}}}^{{\prime} }}\rho ({{{\bf{r}}}},{{{{\bf{r}}}}}^{{\prime} })[{{{\bf{r}}}}{\delta }_{{{{\bf{r}}}},{{{{\bf{r}}}}}^{{\prime} }}+{{{{\boldsymbol{D}}}}}_{cv}({{{{\bf{r}}}}}^{{\prime} }-{{{\bf{r}}}})],$$

where $\rho ({{{\bf{r}}}},{{{{\bf{r}}}}}^{{\prime} })={[{\psi }_{{{{\boldsymbol{Q}}}}}^{*}]}^{ex}({{{\bf{r}}}}){[{\psi }_{{{{\boldsymbol{Q}}}}}]}^{ex}({{{{\bf{r}}}}}^{{\prime} })$ is the reduced density matrix in the relative coordinates and ***D***_*cv*_(**R**) ≡ *e*^*i****Q***⋅**R**/2^***d***_*c*_(**R**) − *e*^−*i****Q***⋅**R**/2^***d***_*v*_(**R**) represents the difference between the electron and hole dipole moments in the Wannier basis. Here ${{{{\boldsymbol{d}}}}}_{c(v)}({{{\bf{R}}}})\equiv \int \,d{{{\bf{x}}}}{w}_{c(v),0}^{*}({{{\bf{x}}}}){{{\bf{x}}}}{w}_{c(v),{{{\bf{R}}}}}({{{\bf{x}}}})$. A full discussion of the physical significance of each of these terms can be found in “Methods.” From a numerical perspective, Eq. (10) enables efficient computation of the excitonic transition shift vector using real-space Wannier functions and exciton envelope functions from first-principles approaches^29–32^.

We emphasize that ${{{\boldsymbol{{\mathscr{R}}}}}}_{0\to \{\cdots \,\}\to ex}$ tracks the electric polarization change for excitonic transitions, including the cumulative electronic effects of subsequent transitions between excitonic states. This is a priori distinct from the electric dipole of an exciton by itself^33^ (*R*_0→ex_ captures electric polarization changes produced by transitions (e.g., optically generated, scattering induced) closely tracking the properties of the relative coordinate important for shift responses. It should not be confused with the center-of-mass shift vector described in ref. ^33^ that affects center-of-mass motion and dynamics). Strikingly, our analysis demonstrates that the details of the transitions are exponentially suppressed [Eq. (9)] yielding ${{{\boldsymbol{{\mathscr{R}}}}}}_{0\to \{\cdots \,\}\to ex}\doteq {{{\boldsymbol{{\mathscr{R}}}}}}_{0\to ex}$. As a result, Eq. (10) coincides with the exciton electric dipole moment^33,34^ in the thermodynamic limit by invoking the standard relation between position matrix elements in the Wannier basis and the Berry connection in momentum space (see e.g., ref. ^17^). This counterintuitive result further delineates pair localized and delocalized excitations, as the shift vectors of the latter depend sensitively on the optical processes involved.

The insensitivity of ${{{\boldsymbol{{\mathscr{R}}}}}}_{0\to ex}$ to $\widehat{V}$ has important implications for its symmetry properties. Accounting for this, we find the excitonic transition shift vector transforms as (see **SI**, Sec. IV for analysis):

11 $${{{\boldsymbol{{\mathscr{R}}}}}}_{0\to ex}({\widehat{U}}_{g}{{{\boldsymbol{Q}}}})\doteq {\widehat{U}}_{g}[{{{\boldsymbol{{\mathscr{R}}}}}}_{0\to ex}({{{\boldsymbol{Q}}}})],$$

where ${\widehat{U}}_{g}$ represents an operation of a point group symmetry (of the material) and we have explicitly specified center-of-mass ***Q*** for clarity.

This distinction becomes dramatic for vertical transitions. Taking ***Q*** = 0 in Eq. (11), we find that ${{{\boldsymbol{{\mathscr{R}}}}}}_{0\to ex}$ transforms as a vector: ${{{\boldsymbol{{\mathscr{R}}}}}}_{0\to ex}({{{\boldsymbol{Q}}}}=0)={\widehat{U}}_{g}[{{{\boldsymbol{{\mathscr{R}}}}}}_{0\to ex}({{{\boldsymbol{Q}}}}=0)]$, where we henceforth omit the explicit ***Q*** = 0 notation. Consequently, ${{{\boldsymbol{{\mathscr{R}}}}}}_{0\to ex}$ is directly locked to the symmetry of the crystal and can only be non-zero along a polar axis. Importantly, it vanishes in non-polar point groups even when inversion symmetry is broken. In contrast, ${{{\boldsymbol{{\mathscr{R}}}}}}_{0\to free}$ does not transform as a vector^8,9^, as explicitly demonstrated in **SI**, Sec. IX. It remains finite in both polar and non-polar materials and points in a direction determined by both $\widehat{V}$ and material properties^8,9^. Notice that among 21 noncentrosymmetric point groups, only 10 are polar^35^, yielding a point group dichotomy between the non-degenerate shift vectors of excitonic and delocalized particle-hole excitations, see Table 1.

**Table 1:** ***Q*** = 0 shift vector behavior for excitonic and delocalized states in different point group (PG) classes

NCS PG	Excitonic shift	Delocalized shift
Non-polar	✗	*✓*
Polar	*✓*	*✓*

*NCS* denotes noncentrosymmetric point groups.

This feature directly impacts physical observables such as the excitonic transition shift photocurrent^6^ for non-degenerate excitons: ${{{{\boldsymbol{j}}}}}_{shift}^{ex}=(-2\pi e/\hslash ){\sum }_{n}| \langle n| {\widehat{V}}^{\omega }| 0\rangle {| }^{2}{{{\boldsymbol{{\mathscr{R}}}}}}_{0\to n}\delta ({E}_{n}-{E}_{0}-\hslash \omega )$ where ${\widehat{V}}^{\omega }$ is the *ω* component of the light-matter interaction, see also **SI**, Sec. VA. For *ℏω* below the gap, it corresponds to excitonic transitions *n* = ex. This means that for nonpolar and noncentrosymmetric point groups, ${{{{\boldsymbol{j}}}}}_{shift}^{ex}$ vanishes.

This sharply contrasts with the non-interacting particle-hole shift current that is non-vanishing for non-polar noncentrosymmetric point groups^27^. This demonstrates the critical role interactions play in determining the shift current: strong particle-hole interactions create a bound state that has a vanishing shift vector in non-polar systems. This is particularly surprising since the matrix elements for both delocalized particle-hole transitions $\langle n=free| {\widehat{V}}^{\omega }| 0\rangle$ and excitonic transitions $\langle n=ex| {\widehat{V}}^{\omega }| 0\rangle$ have the same symmetry properties. Nevertheless, their associated shift vectors have contrasting symmetry properties as shown in Table 1; this arises from the distinct behavior of geometric phases accrued for delocalized vs excitonic transitions in Eq. (9). As a result, symmetry analysis of shift current based on the matrix elements^36,37^ alone cannot distinguish between delocalized vs excitonic transitions.

In contrast, finite momentum transfer ***Q*** ≠ 0 can enable access to non-vanishing finite ***Q*** shift vectors^38^. The shift vector for such non-vertical transitions obeys Eq. (11) above and can be non-zero even in non-polar materials. Nevertheless, we expect that for far-field light, the values of such ***Q*** are severely constrained by the light cone^39^ due to energy-momentum conservation, thereby suppressing finite ***Q*** shift vectors accessed.

### Excitonic transition shift vector in topologically obstructed bands

Our previous discussion relies on the exponentially decaying nature of Wannier functions. In topologically obstructed bands (e.g., *c*, *v* bands with non-trivial Chern number), it is impossible to construct Wannier functions that are simultaneously exponentially localized along all spatial directions^40^.

This topological obstruction can be circumvented, however, by employing hybrid Wannier functions, which are exponentially localized along one direction but delocalized along the others^41,42^. Indeed, by aligning the pair localization direction with the flux ***κ*** (i.e. the direction of the shift), our conclusions remain valid namely: insensitivity of exciton envelope function to flux threading Eq. (6), independence of excitonic transition shift vector on light-matter interactions Eq. (9), and the transformation rule of excitonic transition shift vector under spatial symmetries Eq. (11); see **SI**, Sec. VI for full discussion.

### Excitonic vs. delocalized particle-hole excitation quantum geometry

It is interesting to explore the intrinsic quantum geometric structure of excitonic transitions and how it differs from delocalized particle-hole transitions. Resonant optical transitions for non-interacting particle-hole pairs possess a Riemannian geometry in momentum space^1^ that enables mapping a range of nonlinear optical processes to geometric quantities^2,3^. This analysis can be extended to many-body resonant transitions by employing flux threading. To do so, we define the many-body tangent vector for a resonant optical transition as

12 $${\widehat{e}}_{a}^{0n}({{{\boldsymbol{\kappa }}}})\equiv | {\Phi }_{0}({{{\boldsymbol{\kappa }}}})\rangle {r}_{0n}^{a}({{{\boldsymbol{\kappa }}}})\langle {\Phi }_{n}({{{\boldsymbol{\kappa }}}})| .$$

Here ${{\boldsymbol{r}}}_{0n}({{\boldsymbol{\kappa }}}) = \langle \Phi_{0}({{\boldsymbol{\kappa}}})| i\nabla_{{\boldsymbol{\kappa}}} | {\Phi }_{n}({{{\boldsymbol{\kappa }}}})\rangle$ is the optical matrix element.

Notice that, unlike the non-interacting case, where tangent vectors associated with non-interacting particle-hole transitions are parameterized by *k* momentum, ${\widehat{e}}_{a}^{0n}({{{\boldsymbol{\kappa }}}})$ in Eq. (12) is parameterized by flux *κ*. Parallel transport (in *κ*) of this tangent vector is captured by the covariant derivative ∇_***κ***_, defined by projecting the derivative onto the tangent plane:

13 $${\nabla }_{{{{\boldsymbol{\kappa }}}}}{\widehat{e}}_{a}^{0n}({{{\boldsymbol{\kappa }}}})={P}_{0}[{\partial }_{{{{\boldsymbol{\kappa }}}}}{\widehat{e}}_{a}^{0n}({{{\boldsymbol{\kappa }}}})]{P}_{n},$$

where ∂_***κ***_ is the ordinary derivative and ${P}_{0/n}=| {\Phi }_{0/n}\rangle \langle {\Phi }_{0/n}|$ are projectors. By construction, ${\nabla }_{{{{\boldsymbol{\kappa }}}}}{\widehat{e}}_{a}^{0n}$ lies in the tangent plane and can be expanded in terms of tangent vectors via ${\nabla }_{c}{\widehat{e}}_{a}^{0n}={\sum }_{b}{({C}^{0n})}_{ca}^{b}{\widehat{e}}_{b}^{0n}$, from which we define the Hermitian connection ${C}_{bac}^{0n}$ as:

14 $${C}_{bac}^{0n}\equiv {\sum }_{e}{Q}_{be}^{0n}{({C}^{0n})}_{ac}^{e}=\left(\,{\widehat{e}}_{b}^{0n},{\nabla }_{{\kappa }_{a}}{\widehat{e}}_{c}^{0n}\right),$$

where the many-body Hermitian metric ${Q}_{ba}^{0n}\equiv ({\widehat{e}}_{b}^{0n},{\widehat{e}}_{a}^{0n})={r}_{n0}^{b}{r}_{0n}^{a}$ determines the optical transition rate. Resonant optical responses can thus be succinctly represented using this geometric language, e.g., the many-body shift current for linearly polarized light is ${j}_{shift}^{a}=-\frac{2\pi {e}^{3}}{\hslash }{\sum }_{n}\delta ({E}_{n0}-\hslash \omega )\,Im[{C}_{bab}^{n0}]\,| {E}^{b}{| }^{2}$.

As we now explain, excitonic transitions have fundamentally distinct Riemannian geometry from delocalized particle-hole transitions. By directly evaluating Eq. (13) using Eq. (6), we find (see **SI**, Sec. X for details):

15 $${\nabla }_{a}({\hat{e}}_{c}^{0{n}_{ex}})={\overbrace{i{{{\mathcal{R}}}}_{0{n}_{ex}}^{a}{\widehat{e}}_{c}^{0{n}_{ex}}}}^{exciton},\,\,\,{\nabla }_{a}({\hat{e}}_{c}^{0{n}_{d}})={\overbrace{[{D}_{{\kappa }_{a}}{{\mathrm{ln}}}({r}_{0{n}_{d}}^{c})]{\hat{e}}_{c}^{0{n}_{d}}}}^{delocalized\,p-h},$$

where *n*_ex_, *n*_d_ denote exciton and delocalized particle-hole pair states, respectively. Importantly, the parallel transport of the exciton-bound pairs takes a factorized form: the tangent vector is multiplied by *i* times the excitonic transition shift vector, which is purely real (Eq. (10)) and is independent of the direction of the tangent vector ${\widehat{e}}_{c}^{0{n}_{ex}}$. Geometrically, this means that parallel transport of the exciton tangent vectors is norm and angle-preserving (see **SI** Sec. X for details). In stark contrast, parallel transport of the delocalized pair tangent vector is multiplied by a prefactor ${D}_{{\kappa }_{a}}{{\mathrm{ln}}}({r}_{0n}^{c})$ (with *D* the covariant derivative: ${D}_{{\kappa }_{a}}{{\mathrm{ln}}}({r}_{0n}^{c})={\partial }_{{\kappa }_{a}}{{\mathrm{ln}}}({r}_{0n}^{c})+i({{{{\mathcal{A}}}}}_{0}^{a}-{{{{\mathcal{A}}}}}_{n}^{a})$) that is in general complex^1,43,44^ and depends on the direction of the tangent vector ${\widehat{e}}_{c}^{0{n}_{d}}$. Therefore, parallel transport of the tangent vector of delocalized particle-hole transitions is, in general, not norm-preserving, nor is it angle-preserving.

The contrasting properties of the tangent vectors in Eq. (15) directly influence the Hermitian connection. By evaluating Eq. (14) using Eq. (15) we find

16 $${C}_{bac}^{0{n}_{ex}}={\overbrace{i{{{\mathcal{R}}}}_{0{n}_{ex}}^{a}{r}_{{n}_{ex}0}^{b}{r}_{{0{n}_{ex}}}^{c}}}^{exciton},\,{C}_{bac}^{0{n}_{d}}={\overbrace{{r}_{{n}_{d}0}^{b}{D}_{{\kappa }_{a}}({r}_{0{n}_{d}}^{c})}}^{delocalizedp-h},$$

Notice ${C}_{bac}^{0{n}_{d}}$ mirrors the Hermitian connection of non-interacting particle-hole transitions in ref. ^1^; the unfactorized form of ${C}_{bac}^{0{n}_{d}}$ means that the shift vector for delocalized particle-hole transitions ${{{{\mathcal{R}}}}}_{0{n}_{d}}^{a,c}=i{C}_{cac}^{0{n}_{d}}/{Q}_{cc}^{0{n}_{d}}$^1,43,44^ directly depends on light polarization^8,27^. In contrast, ${C}_{bac}^{0{n}_{ex}}$ takes on a factorized form: ${{{{\mathcal{R}}}}}_{0{n}_{ex}}^{a}$ is independent of the direction of ${r}_{{n}_{ex}0}^{a,b}$. Since the direction of ${r}_{{n}_{ex}0}^{a,b}$ is locked to the light polarization, this factorization echoes the insensitivity of the shift vector to light polarization established in Eq. (9). Both Eq. (15) and (16) demonstrate the stark difference between the geometry of delocalized particle-hole transitions versus excitonic transitions.

### Excitonic transition shift vector in MoS_2_

**Fig. 3:**
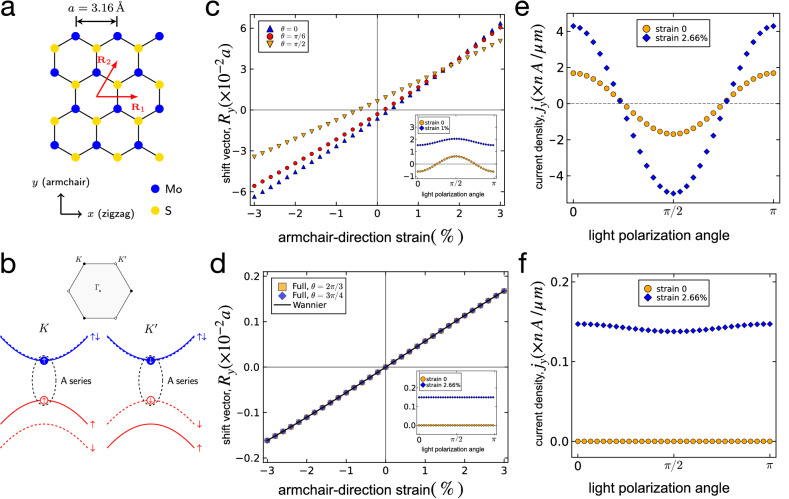
Numerical calculation of the shift vector and the shift current in a three-band tight-binding model of MoS_2_. **a** Schematic of the monolayer MoS_2_ lattice; the armchair direction is along *y*. **b** Schematic of *A*-series excitons at the $K,{K}^{{\prime} }$ valleys in the Brillouin zone. Each exciton is two-fold valley-degenerate. Conduction (valence) bands are shown in blue (red), with spin-up (down) bands indicated by solid (dashed) lines. Strong spin-orbit coupling splits the valence bands (with smaller conduction-band splittings), inducing spin-valley locking: *K*-valley *A*-excitons involve up-spin electron-hole pairs, while ${K}^{{\prime} }$-valley excitons involve down-spin pairs. **c** Strain dependence of free particle-hole pair shift vectors for direct optical transitions at momentum *K*, which remain non-zero in the unstrained case and depend strongly on light-polarization angle *θ* (inset: polarization dependence of shift vector at *K* for the unstrained and 1% strained case; the *y*-axis unit is the same as in (3c)). **d** Strain dependence of the A1s excitonic transition shift vector at *K* composed of spin-up particle-hole pairs, which are polarization independent and vanish in the unstrained limit. Squares and diamonds are computed via the full formula Eq. (7), and the line is computed via the Wannier-based formula Eq. (10); both agree with each other. (inset: polarization dependence of A1s excitonic transition shift vector for the unstrained and 2.66% strained (*δt*/*t* = − 0.1) case via full formula; the *y*-axis unit is the same as in (**d**)). **e** Shift current for free particle-hole pairs at a photon energy of *ℏω* = 2.5eV, for both zero strain and 2.66% strain (*δt*/*t* = − 0.1). Strong polarization-dependent shift vectors cause the current to reverse sign as the polarization direction changes. **f** *A*_1*s*_ excitonic transition shift current at photon energy *ℏω* = 1.08eV with the same strains as in (**e**). Since the excitonic transition shift vector is polarization-independent, the resulting excitonic transition shift current’s weak polarization dependence comes from the squared optical matrix element and does not change sign. See “Methods” as well as **SI**, Sec. VIII for numerical details.

As a concrete illustration of the differences between the shift vector/responses for delocalized particle-hole transitions versus excitonic transitions, we performed a numerical study of shift vectors and shift currents in monolayer MoS_2_ (see Fig. 3). Before we proceed, we note that in the presence of time-reversal symmetry, MoS_2_ excitons are valley-degenerate (see e.g., discussion of responses in degenerate many-body systems **SI**, Sec. VB). Nevertheless, strong spin-valley locking (see Fig. 3(b)) ensures that the shift vector is diagonal in the spin-subspace so that the shift vectors of K and K’ excitonic transitions are effectively decoupled. This enables us to analyze the shift vector in each spin/valley block using the non-degenerate shift vector formula in Eq. (7).

We proceed by computing the shift vector for both delocalized free particle-hole transitions (Fig. 3(c)) and excitonic transitions (Fig. 3(d)) using a realistic three-band nearest-neighbor tight-binding model of MoS_2_ with parameters fitted to Density Functional Theory (DFT) bandstructure calculations^45^; we have also implemented a tunable strain^46^, see “Methods” as well as **SI**. Sec. VIII for detailed numerical parameters. Here we have focused on vertical transitions.

Figure 3(c) shows that delocalized free particle-hole transitions have a shift vector that is finite at zero strain and strongly depends on light polarization (Fig. 3(c) inset). In contrast, the excitonic transition shift vector in Fig. 3(d) vanishes at zero strain and is independent of light polarization (Fig. 3(d) inset). The vanishing of ***Q*** = 0 excitonic transition shift vector is a direct consequence of the nonpolar nature of the *C*_3*z*_ symmetric MoS_2_ without strain (see Table. 1 and analysis below Eq. (11)). Its light polarization independence directly follows from the bound nature of the exciton as described above. We note that once *C*_3*z*_ is broken by strain, MoS_2_ becomes polar, and the excitonic transition shift vector is turned on (Fig. 3(d)). Notice that ${{{\boldsymbol{{\mathscr{R}}}}}}_{0\to ex}$ is directed only along the polar direction (armchair, *y*). This contrasts with ${{{\boldsymbol{{\mathscr{R}}}}}}_{0\to free}$ that is not constrained to point along *y* only and depends on the light polarization angle. The shift vectors in Fig. 3(c) and (d) enable direct evaluation of the shift current producing the delocalized free particle-hole transition shift current (Fig. 3(e)), and the excitonic transition shift current (Fig. 3(f)). Notice that while the delocalized free particle-hole transition shift current is strongly polarization dependent, the excitonic transition shift current is only weakly dependent on light polarization.

This behavior directly mirrors the as-yet-unexplained experimental observations in ref. ^10^, where negligible photocurrent was observed in *C*_3*z*_-symmetric 3R-MoS_2_; a giant photocurrent was activated when 3R-MoS_2_ was strained. Our symmetry-based analysis from Eq. (11), as well as numerical simulations in this section, underscores the importance of strain and lowered symmetry in the appearance of finite bulk photovoltaic currents^10,11,47^.

## Discussion

Our work demonstrates that strong electron-hole interaction radically transforms excited state quantum geometry: excitonic transition shift vectors are insensitive to light polarization and are intrinsic in nature. Indeed, the many-body tangent vector and Hermitian connection associated with the excitonic transition take on a factorized form with distinct geometric properties from that of delocalized particle-hole transitions^1^. This result is non-perturbative and has important physical ramifications: vertical excitonic transition shift current in non-polar materials vanishes, its current and shift vector are aligned to polar axes, and the shift vector is a “geometric ruler” for pair localization.

From a computational perspective, the real-space formulation of shift current responses we discuss only requires (i) the optical transition matrix element between the ground state and the specific excitonic state and (ii) the excitonic transition shift vector in the real-space Wannier basis. Both are readily extracted from ab initio GW+BSE calculations^30^. For example, maximally localized Wannier functions (MLWFs) can be directly obtained from Wannier packages (e.g., ref. ^29^) and DFT-GW calculations of the band structure. Using these MLWFs, the excitonic envelope function can be obtained from standard BSE packages, e.g., refs. ^48,49^. These envelope functions, combined with the MLWFs, allow direct evaluation of the excitonic shift vector via the Wannier-based formula Eq. (10). As a demonstration of the power of Eq. (10), we plot the excitonic transition shift vector using both the Wannier-based Eq. (10) formula (solid line) as well as the full flux-based shift vector formula in Eq. (7) (diamonds and squares) in Fig. 3(d) for a simple three-band DFT fitted tight-binding model of MoS_2_. Both line up on top of each other, demonstrating the high precision of the Wannier-based formula, see detailed discussion in **SI** Sec. VIII for numerical procedure.

This sharply contrasts with momentum-space formulations of the excitonic shift current^36,37,50,51^ that require an explicit summation over an entire set of intermediate states. We expect real-space calculations will open up efficient numerics of shift current and other geometric responses^14,52^ for excitons.

Perhaps most exciting is the prospect of electron interactions qualitatively transforming other aspects of quantum geometry^1–6^ in strongly interacting materials. For e.g., the Hermitian curvature^1^ of optical transitions involves covariant derivatives of the Hermitian connection. Given the flux threading properties we introduce for excitonic transitions, we anticipate that its Hermitian curvature may vanish, producing an interaction-induced flat quantum geometry. Looking forward, we expect interactions to play critical roles in new classes of geometrical behavior beyond topology, as well as understanding a growing range of dynamical nonequilibrium phenomena.

## Methods

### Deviation estimate of the ansatz function for exciton states with flux insertion

In this section, we describe how to estimate the value of the deviation described in the main text for the exciton. Here and in what follows, the subscript ***Q*** is suppressed as it is conserved and decoupled from ***κ***. Without loss of generality, we take ***κ*** along the *x* direction. First, a periodic boundary condition is implemented in BSE by restricting relative coordinates to  − *L*/2 < *r*_*x*_ < *L*/2. The BSE is then defined with the minimal distance:

17 $${{{\mathcal{H}}}}({{{\bf{r}}}},{{{{\bf{r}}}}}^{{\prime} })\equiv {{{\mathcal{H}}}}\left({{{\bf{r}}}},{{{{\bf{r}}}}}^{{\prime} }+ML{{{{\bf{e}}}}}_{x}\right),$$

with $M\in {\mathbb{Z}}$ minimizing $| {r}_{x}-{r}_{x}^{{\prime} }-ML|$. Correspondingly, flux insertion modifies matrix elements by

18 $${{{{\mathcal{H}}}}}^{{{{\boldsymbol{\kappa }}}}}({{{\bf{r}}}},{{{{\bf{r}}}}}^{{\prime} })={e}^{-i{\kappa}_{x}({r}_{x}-{r}_{x}^{{\prime} }-ML)}{{{\mathcal{H}}}}({{{\bf{r}}}},{{{{\bf{r}}}}}^{{\prime} })$$

For instance, when *r*_*x*_ ≲ *L*/2 and ${r}_{x}^{{\prime} }\gtrsim -L/2$, the minimal distance is ${r}_{x}-{r}_{x}^{{\prime} }-L$ with *M* = 1.

The exciton solution *ψ*(**r**) of the BSE at ***κ*** = 0 satisfies ${\sum }_{{{{{\boldsymbol{r}}}}}^{{\prime} }}{{{\mathcal{H}}}}({{{\boldsymbol{r}}}},{{{{\boldsymbol{r}}}}}^{{\prime} })\psi ({{{{\boldsymbol{r}}}}}^{{\prime} })=E\psi ({{{\boldsymbol{r}}}})$ and is exponentially decaying ∣*ψ*(**r**)∣ ~ *e*^−∣**r**∣/*ξ*^. We now estimate the deviation of the ansatz ${\widetilde{\psi }}^{{{{\boldsymbol{\kappa }}}}}({{{\bf{r}}}})={e}^{-i{{{\boldsymbol{\kappa }}}}\cdot {{{\bf{r}}}}}\psi ({{{\bf{r}}}})$ from the exciton solution to the flux-inserted ${{{{\mathcal{H}}}}}^{{{{\boldsymbol{\kappa }}}}}$ by examining the following deviation:

19 $$\zeta ({{{\bf{r}}}})\equiv \left| {\sum }_{{{{{\bf{r}}}}}^{{\prime} }}{{{{\mathcal{H}}}}}^{{{{\boldsymbol{\kappa }}}}}({{{\bf{r}}}},{{{{\bf{r}}}}}^{{\prime} }){\widetilde{\psi }}^{{{{\boldsymbol{\kappa }}}}}({{{{\bf{r}}}}}^{{\prime} })-E{\widetilde{\psi }}^{{{{\boldsymbol{\kappa }}}}}({{{\bf{r}}}})\right| .$$

Without loss of generality, we assume 0 < *r*_*x*_ < *L*/2. To evaluate *ζ*(**r**), we split the sum over ${{{{\bf{r}}}}}^{{\prime} }$ into a *bulk region *${{\mathbb{R}}}_{bulk}$ where $| {r}_{x}-{r}_{x}^{{\prime} }| < L/2$, and an *edge region *${{\mathbb{R}}}_{edge}$ where $| {r}_{x}-{r}_{x}^{{\prime} }| > L/2$. The Hamiltonian matrix elements relate to those without flux as:

$${{{{\mathcal{H}}}}}^{{{{\boldsymbol{\kappa }}}}}({{{\bf{r}}}},{{{{\bf{r}}}}}^{{\prime} })=\left\{\begin{array}{ll}{e}^{-i{{{\boldsymbol{\kappa }}}}\cdot ({{{\bf{r}}}}-{{{{\bf{r}}}}}^{{\prime} })}{{{\mathcal{H}}}}({{{\bf{r}}}},{{{{\bf{r}}}}}^{{\prime} }),& {{{{\bf{r}}}}}^{{\prime} }\in {{\mathbb{R}}}_{bulk},\\ {e}^{-i{{{\boldsymbol{\kappa }}}}\cdot ({{{\bf{r}}}}-{{{{\bf{r}}}}}^{{\prime} }-L{{{{\bf{e}}}}}_{x})}{{{\mathcal{H}}}}({{{\bf{r}}}},{{{{\bf{r}}}}}^{{\prime} }),& {{{{\bf{r}}}}}^{{\prime} }\in {{\mathbb{R}}}_{edge}.\end{array}\right.$$

Inserting this into the flux-threaded equation yields:

20 $${\sum }_{{{{{\bf{r}}}}}^{{\prime} }}{{{{\mathcal{H}}}}}^{{{{\boldsymbol{\kappa }}}}}({{{\bf{r}}}},{{{{\bf{r}}}}}^{{\prime} }){\widetilde{\psi }}^{{{{\boldsymbol{\kappa }}}}}({{{{\bf{r}}}}}^{{\prime} })={e}^{-i{{{\boldsymbol{\kappa }}}}\cdot {{{\bf{r}}}}}\left[{\sum }_{{{\mathbb{R}}}_{bulk}}{{{\mathcal{H}}}}({{{\bf{r}}}},{{{{\bf{r}}}}}^{{\prime} })\psi ({{{{\bf{r}}}}}^{{\prime} })+{e}^{i{\kappa }_{x}L}{\sum }_{{{\mathbb{R}}}_{edge}}{{{\mathcal{H}}}}({{{\bf{r}}}},{{{{\bf{r}}}}}^{{\prime} })\psi ({{{{\bf{r}}}}}^{{\prime} })\right].$$

Subtracting $E{\widetilde{\psi }}^{{{{\boldsymbol{\kappa }}}}}({{{\boldsymbol{r}}}})={e}^{-i{{{\boldsymbol{\kappa }}}}\cdot {{{\bf{r}}}}}E\psi ({{{\bf{r}}}})$, we find that the deviation in Eq. (19) manifests in ${{\mathbb{R}}}_{edge}$:

21 $$\zeta ({{{\bf{r}}}})=\left| {e}^{-i{{{\boldsymbol{\kappa }}}}\cdot {{{\bf{r}}}}}({e}^{i{\kappa }_{x}L}-1){\sum }_{{{\mathbb{R}}}_{edge}}{{{\mathcal{H}}}}({{{\bf{r}}}},{{{{\bf{r}}}}}^{{\prime} })\psi ({{{{\bf{r}}}}}^{{\prime} })\right| .$$

For localized exciton envelope functions, *ψ*(**r**) ~ *e*^−∣**r**∣/*ξ*^ and ${{{\mathcal{H}}}}({{{\bf{r}}}},{{{{\bf{r}}}}}^{{\prime} })$ decays with range *ξ*_*W*_. Bounding the sum, we obtain:

22 $$\begin{array}{rcl} & & \zeta ({{{\bf{r}}}}) < | {e}^{i{\kappa }_{x}L}-1| \cdot {e}^{-| {{{\bf{r}}}}-{{{{\bf{r}}}}}^{{\prime} }-L{{{{\bf{e}}}}}_{x}| /{\xi }_{W}}\cdot {e}^{-| {{{{\bf{r}}}}}^{{\prime} }| /\xi }\\ & & < | {e}^{i{\kappa }_{x}L}-1| \cdot {e}^{-| {{{\bf{r}}}}-L{{{{\bf{e}}}}}_{x}| /{\xi }_{M}} < | {e}^{i{\kappa }_{x}L}-1| \cdot {e}^{-L/(2{\xi }_{M})}\end{array}$$

where ${\xi }_{M}=\max (\xi,{\xi }_{W})$ and we used the triangular inequality and 0 < *r*_*x*_ < *L*/2. Consequently, we arrive at

23 $${\sum }_{{{{{\bf{r}}}}}^{{\prime} }}{{{{\mathcal{H}}}}}^{{{{\boldsymbol{\kappa }}}}}({{{\bf{r}}}},{{{{\bf{r}}}}}^{{\prime} }){\widetilde{\psi }}^{{{{\boldsymbol{\kappa }}}}}({{{{\bf{r}}}}}^{{\prime} })=E{\widetilde{\psi }}^{{{{\boldsymbol{\kappa }}}}}({{{\bf{r}}}})+{{{\mathcal{O}}}}({e}^{-L/(2{\xi }_{M})}),$$

or equivalently,

24 $$\begin{array}{rcl}{\psi }^{{{{\boldsymbol{\kappa }}}}}({{{\bf{r}}}}) &=& {e}^{-i{{{\boldsymbol{\kappa }}}}\cdot {{{\bf{r}}}}}\psi ({{{\bf{r}}}})+{{{\mathcal{O}}}}({e}^{-L/(2{\xi }_{M})}),\\ E({{{\boldsymbol{\kappa }}}}) &=& E(0)+{{{\mathcal{O}}}}({e}^{-L/{\xi }_{M}}),\hfill\end{array}$$

which is Eq. (6). This mirrors the Thouless criterion for localized states^53–55^.

In contrast, substituting the delocalized envelope for free particle-hole excitations such as plane waves ∣*ψ*(**r**)∣ ~ *L*^−*d*/2^ into Eq. (21) results in significant deviations. Indeed, when *r*_*x*_ ~ *L*/2 and ${r}_{x}^{{\prime} } \sim -L/2$, the amplitude $| \psi ({{{{\bf{r}}}}}^{{\prime} })| \sim {L}^{-d/2}$ leads to:

25 $$\zeta ({{{\bf{r}}}}) \sim | {e}^{i{\kappa }_{x}L}-1| \cdot {{{\mathcal{O}}}}({L}^{-d/2}),$$

which constitutes a non-negligible correction for an extended envelope function. Thus, for extended states, the ansatz ${\widetilde{\psi }}_{{{{\boldsymbol{\kappa }}}}}$ fails to solve the flux-threaded Bethe-Salpeter equation, mirroring the breakdown of “gauge invariance” for extended states in a periodic system^20^.

### Real-space Wannier expression for the excitonic transition shift vector

In this section, we obtain a compact and direct real-space Wannier expression for the excitonic transition shift vector. We proceed by evaluating the excitonic transition shift vector using the exciton wave function $| {\psi }_{{{{\boldsymbol{Q}}}}}\rangle=\frac{1}{\sqrt{N}}{\sum }_{{{{\bf{r}}}},{{{{\bf{R}}}}}_{cm}}{\psi }_{{{{\boldsymbol{Q}}}}}({{{\bf{r}}}}){e}^{i{{{\boldsymbol{Q}}}}\cdot {{{{\bf{R}}}}}_{cm}}| {{{{\bf{R}}}}}_{cm},{{{\bf{r}}}}\rangle$ along with the definition of the shift vector:

26 $${{{\boldsymbol{{\mathscr{R}}}}}}_{0n}\equiv i\langle {\Phi }_{n}| {\nabla }_{{{{\boldsymbol{\kappa }}}}}| {\Phi }_{n}\rangle -i\langle {\Phi }_{0}| {\nabla }_{{{{\boldsymbol{\kappa }}}}}| {\Phi }_{0}\rangle+{\nabla }_{{{{\boldsymbol{\kappa }}}}}\arg (\langle {\Phi }_{0}| \widehat{V}| {\Phi }_{n}\rangle ).$$

Under flux insertion, the Wannier functions transform as ${w}_{{{{\bf{R}}}}}({{{\bf{x}}}})\to {w}_{{{{\bf{R}}}}}^{{{{\boldsymbol{\kappa }}}}}({{{\bf{x}}}})={e}^{-i{{{\boldsymbol{\kappa }}}}\cdot ({{{\bf{x}}}}-{{{\bf{R}}}})}{w}_{{{{\bf{R}}}}}({{{\boldsymbol{x}}}})$, whose corresponding creation operator we denote as ${c}_{{{{\bf{R}}}}}^{{{{\boldsymbol{\kappa }}}},{{\dagger}} }$.

Under flux insertion the the exciton state is

27 $$| {\psi }_{{{{\boldsymbol{Q}}}}}^{{{{\boldsymbol{\kappa }}}}}\rangle=\frac{1}{\sqrt{N}}{\sum }_{{{{\bf{r}}}},{{{{\bf{R}}}}}_{cm}}{\psi }_{{{{\boldsymbol{Q}}}}}^{{{{\boldsymbol{\kappa }}}}}({{{\bf{r}}}}){e}^{i{{{\boldsymbol{Q}}}}\cdot {{{{\bf{R}}}}}_{cm}}{c}_{c,{{{{\bf{R}}}}}_{e}}^{{{{\boldsymbol{\kappa }}}},{{\dagger}} }{c}_{v,{{{{\bf{R}}}}}_{h}}^{{{{\boldsymbol{\kappa }}}}}| G{S}^{{{{\boldsymbol{\kappa }}}}}\rangle,$$

where $$| {GS}^{{{{\boldsymbol{\kappa }}}}}\rangle={\prod }_{{{{\bf{R}}}}}{c}_{v,{{{\bf{R}}}}}^{{{{\boldsymbol{\kappa }}}},{{\dagger}} }| 0\rangle$$.

Recalling that with ${\psi }_{{{{\boldsymbol{Q}}}}}^{{{{\boldsymbol{\kappa }}}}}({{{\bf{r}}}})={e}^{-i{{{\boldsymbol{\kappa }}}}\cdot {{{\bf{r}}}}}{\psi }_{{{{\boldsymbol{Q}}}}}({{{\bf{r}}}})$, the transition matrix element Eq. (8) is insensitive to ***κ***, therefore the exciton transition shift vector is

28 $${{{\boldsymbol{{\mathscr{R}}}}}}_{0\to ex}\doteq i\langle {\psi }_{{{{\boldsymbol{Q}}}}}^{{{{\boldsymbol{\kappa }}}}}\left| {\nabla }_{{{{\boldsymbol{\kappa }}}}}\left[\frac{1}{\sqrt{N}}{\sum }_{{{{\bf{r}}}},{{{{\bf{R}}}}}_{cm}}{\psi }_{{{{\boldsymbol{Q}}}}}^{{{{\boldsymbol{\kappa }}}}}({{{\bf{r}}}}){e}^{i{{{\boldsymbol{Q}}}}\cdot {{{{\bf{R}}}}}_{cm}}{c}_{c,{{{{\bf{R}}}}}_{e}}^{{{{\boldsymbol{\kappa }}}},{{\dagger}} }{c}_{v,{{{{\bf{R}}}}}_{h}}^{{{{\boldsymbol{\kappa }}}}}\right]\right| {GS}^{{{{\boldsymbol{\kappa }}}}}\rangle,$$

which can be delineated into three terms.

1. The Wannier center part is obtained by examining ∇_***κ***_ acting on ${\psi }_{{{{\boldsymbol{Q}}}}}^{{{{\boldsymbol{\kappa }}}}}({{{\bf{r}}}})$, which yields:
  29 $${\sum }_{{{{\bf{r}}}}}{{{\bf{r}}}}| {\psi }_{{{{\boldsymbol{Q}}}}}({{{\bf{r}}}}){| }^{2}\equiv {\sum }_{{{{\bf{r}}}}}\rho ({{{\bf{r}}}},{{{\bf{r}}}}){{{\bf{r}}}},$$
  where we have used the exciton envelope function’s reduced density matrix $\rho ({{{\bf{r}}}},{{{{\bf{r}}}}}^{{\prime} })={\psi }_{{{{\boldsymbol{Q}}}}}^{*}({{{\bf{r}}}}){\psi }_{{{{\boldsymbol{Q}}}}}({{{{\bf{r}}}}}^{{\prime} })$. This term originates from the polarization contributions of electrons and holes localized at their respective Wannier centers and is diagonal in *ρ*.
2. The electron cloud correction is obtained by taking ***κ*** derivative of ${c}_{c,{{{{\bf{R}}}}}_{e}}^{{{{\boldsymbol{\kappa }}}},{{\dagger}} }$. After a straightforward evaluation we arrive at:
  30 $${\sum }_{{{{\bf{r}}}},{{{{\bf{r}}}}}^{{\prime} }}\rho ({{{\bf{r}}}},{{{{\bf{r}}}}}^{{\prime} }){e}^{i{{{\boldsymbol{Q}}}}\cdot ({{{{\bf{r}}}}}^{{\prime} }-{{{\bf{r}}}})/2}{{{{\boldsymbol{d}}}}}_{c}({{{{\bf{r}}}}}^{{\prime} }-{{{\bf{r}}}}),$$
  where we have noted the translational symmetry of Wannier functions as well as utilized the dipole matrix element in the Wannier basis as defined in ref. ^17^: $\int \,d{{{\bf{x}}}}{w}_{c,{{{\bf{0}}}}}^{*}({{{\bf{x}}}}){{{\bf{x}}}}{w}_{c,{{{\bf{R}}}}}({{{\bf{x}}}})={{{{\boldsymbol{d}}}}}_{c}({{{\bf{R}}}})$. Here, the density matrix $\rho ({{{\bf{r}}}},{{{{\bf{r}}}}}^{{\prime} })$ reflects the electron density probability distribution. The ${e}^{\frac{i}{2}{{{\boldsymbol{Q}}}}\cdot ({{{{\bf{r}}}}}^{{\prime} }-{{{\bf{r}}}})}$ factor is due to an effective shift of the center-of-mass coordinate due to the electron cloud. This term captures the correction from the spatial spread of conduction-band Wannier orbitals.
3. The hole cloud correction is obtained from taking ***κ*** derivative of ${c}_{v,{{{{\bf{R}}}}}_{h}}^{{{{\boldsymbol{\kappa }}}}}$ producing:
  31 $$-{\sum }_{{{{\bf{r}}}},{{{{\bf{r}}}}}^{{\prime} }}\rho ({{{\bf{r}}}},{{{{\bf{r}}}}}^{{\prime} }){e}^{i{{{\boldsymbol{Q}}}}\cdot ({{{\bf{r}}}}-{{{{\bf{r}}}}}^{{\prime} })/2}{{{{\boldsymbol{d}}}}}_{v}({{{{\bf{r}}}}}^{{\prime} }-{{{\bf{r}}}}).$$
  Similar to the electron case, this term is the correction to polarization due to the spatial spreading of hole Wannier orbitals. Similar to above, the ${e}^{i{{{\boldsymbol{Q}}}}\cdot ({{{\bf{r}}}}-{{{{\bf{r}}}}}^{{\prime} })/2}$ factor arises from an effective shift of center-of-mass coordinates.

Combining these three terms, we arrive at the following compact expression for the excitonic transition shift vector:

32 $${{{\boldsymbol{{\mathscr{R}}}}}}_{0\to ex}\doteq {\sum }_{{{{\bf{r}}}},{{{{\bf{r}}}}}^{{\prime} }}\rho ({{{\bf{r}}}},{{{{\bf{r}}}}}^{{\prime} })\left[{{{\bf{r}}}}{\delta }_{{{{\bf{r}}}},{{{{\bf{r}}}}}^{{\prime} }}+{e}^{i{{{\boldsymbol{Q}}}}\cdot ({{{{\bf{r}}}}}^{{\prime} }-{{{\bf{r}}}})/2}{{{{\boldsymbol{d}}}}}_{c}({{{{\bf{r}}}}}^{{\prime} }-{{{\bf{r}}}})-{e}^{i{{{\boldsymbol{Q}}}}\cdot ({{{\bf{r}}}}-{{{{\bf{r}}}}}^{{\prime} })/2}{{{{\boldsymbol{d}}}}}_{v}({{{{\bf{r}}}}}^{{\prime} }-{{{\bf{r}}}})\right].$$

Note that in a periodic system, the relative coordinate **r** is inherently ambiguous due to boundary conditions^22^. For localized excitons, however, the envelope *ψ*(**r**) (and thus *ρ*) is concentrated near the origin, rendering **r** well-defined as contributions from large ∣**r**∣ are exponentially suppressed. Consequently, Eq. (32) applies only to localized particle-hole excitations and breaks down for delocalized ones.

### Numerical methods

In this section, we summarize our numerical simulations. In obtaining Fig. 2, we considered a two-band tight-binding model defined on a honeycomb lattice with two sublattices per unit cell. The single-particle Hamiltonian consists of nearest-neighbor hopping terms and a staggered sublattice potential that opens a gap at momenta $K,{K}^{{\prime} }$. Electron-hole interactions are modeled by a screened Coulomb potential of Keldysh form^56,57^. Within the BSE, we retain only the direct interaction term for computational simplicity. The flux-inserted Hamiltonian and corresponding current operator are constructed via the prescription in Eq. (3) and Eq. (4).

We solve the BSE to obtain the energy spectra of both exciton and delocalized states at various ***κ*** for a fixed system size $\sqrt{N}=24$, as shown in Fig. 2(a). The inset of Fig. 2(a) presents the finite-size scaling of selected localized and delocalized energy levels.

At system size $\sqrt{N}=24$, we compute the standard deviation of $\arg ({{{{\mathcal{W}}}}}_{{{{\boldsymbol{\kappa }}}}})$ (evaluated for two different light polarizations) over discrete values of *κL*/2*π* ∈ [0.01, 0.91] with step size 0.1, which is used in producing Fig. 2(b). The offset 0.01 avoids accidental degeneracies at *κL* = 0, *π*. Fig. 2(c) shows the corresponding finite-size scaling of $\arg ({{{{\mathcal{W}}}}}_{{{{\boldsymbol{\kappa }}}}})$ for selected localized and delocalized states. For the finite-size scaling analysis in Fig. 2(a,c), we vary the system size from $\sqrt{N}=12$ to 42 in steps of 6.

We now turn to describing our numerical methods for Fig. 3. For monolayer MoS_2_, we adopt the three-band nearest-neighbor tight-binding model of ref. ^45^, constructed from Mo *d*-orbitals $\{| {d}_{{z}^{2}}\rangle,| {d}_{xy}\rangle,| {d}_{{x}^{2}-{y}^{2}}\rangle \}$. Spin–orbit coupling (SOC) is incorporated via an on-site term projected onto this orbital subspace, yielding an effective *λL*_*z*_*S*_*z*_ interaction. The tight-binding parameters and the SOC value are taken from ref. ^45^. The conservation of *S*_*z*_ allows excitonic states to be classified by spin. Uniaxial strain along the armchair (*y*) direction is introduced by modifying the corresponding bond lengths and rescaling hopping amplitudes according to ref. ^10,46^. Similar to our plots in Fig. 2, the electron-hole interaction is also described by the Keldysh potential, with material parameters taken from ref. ^58^.

At each strain value *ϵ*_*y*_, we compute Bloch eigenstates on a 60 × 60 **k**-point mesh. MLWFs^17^ are then constructed via (i) gauge initialization using local trial orbitals $\{| {g}_{v}\rangle,| {g}_{{c}_{1}}\rangle,| {g}_{{c}_{2}}\rangle \}$, followed by (ii) minimization of the Wannier spread functional through unitary rotations of Bloch states using a gradient-based algorithm. This procedure yields a smooth gauge for the Bloch functions. These Bloch eigenstates are used to obtain free p-h transition shift vectors and shift currents in Fig. 3(c,e). The excitonic envelope functions can be subsequently obtained by solving the BSE in the gauge-fixed basis, from which the excitonic transition shift vectors and shift currents are calculated in Fig. 3(d,f).

We evaluate the excitonic transition shift vector in Fig. 3(d) using two complementary approaches. First, we compute the full expression Eq. (7), which explicitly incorporates light-matter coupling. The ***κ***-dependence is introduced via a shift of Bloch states, $| {u}_{c/v,{{{\bf{k}}}}}\rangle \to | {u}_{c/v,{{{\bf{k}}}}+{{{\boldsymbol{\kappa }}}}}\rangle$, implemented by Fourier transforming Eq. (3). The resulting flux-inserted Hamiltonian is projected onto the particle-hole basis to construct the ***κ***-dependent BSE kernel, from which the exciton envelope functions *ψ*^***κ***^(**k**) are obtained. Derivatives with respect to ***κ*** are evaluated using a central finite-difference scheme ${\partial }_{{{{\boldsymbol{\kappa }}}}}f{| }_{{{{\boldsymbol{\kappa }}}}=0}=\frac{f(\delta {{{\boldsymbol{\kappa }}}})-f(-\delta {{{\boldsymbol{\kappa }}}})}{2| \delta {{{\boldsymbol{\kappa }}}}| }+{{{\mathcal{O}}}}(| \delta {{{\boldsymbol{\kappa }}}}{| }^{2}),$ with step size $| \delta {{{\boldsymbol{\kappa }}}}|=0.01| {{{{\bf{b}}}}}_{1}| /\sqrt{N}$, where **b**_1_ is a reciprocal lattice vector.

Second, we evaluate the Wannier-based expression Eq. (10), which depends only on the MLWFs and the exciton envelope function at ***κ*** = **0**. The Wannier-based expression is evaluated to produce the solid line in Fig. 3(d). The two approaches are in quantitative agreement: the full expression Eq. (7), evaluated for different light polarizations, yields polarization-independent results [colored symbols in Fig. 3(d)] consistent with the Wannier-based calculation [solid black line in Fig. 3(d)]. A more detailed quantitative comparison is provided in **SI**. Sec. VIII. Finally, in computing the shift current, the Dirac delta function is regularized via a Lorentzian broadening, $\delta (E-\hslash \omega )=\frac{\Gamma }{\pi }\frac{1}{{(E-\hslash \omega )}^{2}+{\Gamma }^{2}},$ with *Γ* = 20 meV to account for the spectral broadening.

## Supplementary information

Supplementary Information

Transparent Peer Review file

## Data Availability

All data underlying the figures in this study are publicly available on Zenodo at 10.5281/zenodo.19363165. The repository includes README.md files that explain how to use the data to reproduce the figures.

## Code availability

The code used to generate all data and figures in this study is available at the GitHub repository https://github.com/yangxucmt/exciton.
