# Supplementary material for: Correlated quantum shift vector of particle-hole excitations
Source: Nat Commun. 2026 May 15;17:6465. doi: 10.1038/s41467-026-72878-8 (PMC13376538; doi:10.1038/s41467-026-72878-8)
Supplement: Supplementary file 1 — Supplementary Information [file 41467_2026_72878_MOESM1_ESM.pdf]

Supplementary Information for:
Correlated Quantum Shift Vector of Particle-Hole Excitations

Xu Yang,[1] Ajit Srivastava,[2] and Justin C. W. Song[1,*]

[1]Division of Physics and Applied Physics, School of Physical and Mathematical Sciences, Nanyang Technological University, Singapore 637371

[2]Department of Quantum Matter Physics, University of Geneva, Geneva 1211, Switzerland

I. BETHE-SALPETER EQUATION IN RELATIVE COORDINATES

It is instructive to express the Bethe-Salpeter equation (BSE) in terms of relative coordinates. Below we use $\mathbf{R}$ to denote the real coordinates of individual electrons/holes, $\mathbf{R}_{\rm cm}$ to denote the center-of-mass coordinates of electron-hole pair, and $\mathbf{r}$ to denote the relative coordinates between electrons and holes.

We use $c^\dagger$ to denote creation operators for Wannier functions, which can be represented by

$$|n,\mathbf{R}\rangle \equiv c^\dagger_{n,\mathbf{R}}|0\rangle = \int d\mathbf{x}\ w_{n,\mathbf{R}}(\mathbf{x}) a^\dagger_{\mathbf{x}}|0\rangle\,, \tag{S1}$$

where n is the band index and $a_{\mathbf{x}}$ is the field operator in free space. Here $w_{n,\mathbf{R}}(\mathbf{x})$ is the exponentially localized Wannier functions centered around $\mathbf{R}$ which decays as $e^{-|\mathbf{x}-\mathbf{R}|/\xi_W}$ at large $|\mathbf{x}-\mathbf{R}|$ and ξ_W is the Wannier function extent. Using the completeness relation $\sum\limits_{n,\mathbf{R}} w_{n,\mathbf{R}}(\mathbf{x})w^*_{n,\mathbf{R}}(\mathbf{x}') = \delta(\mathbf{x}-\mathbf{x}')$, we can expand $a^\dagger_{\mathbf{x}}$ in terms of $c^\dagger_{n,\mathbf{R}}$, where n is the band index:

$$a^\dagger_{\mathbf{x}}|0\rangle = \sum_{n,\mathbf{R}} w^*_{n,\mathbf{R}}(\mathbf{x}) c^\dagger_{n,\mathbf{R}}|0\rangle\,. \tag{S2}$$

Starting from the eigen equation $\hat{H}|\psi_{\boldsymbol{Q}}\rangle_{\rm p-h} = E|\psi_{\boldsymbol{Q}}\rangle_{\rm p-h}$, we can project it onto the particle-hole basis $|\mathbf{R}_{\rm cm},\mathbf{r}\rangle$ to arrive at the BSE for envelope functions: $\sum\limits_{\mathbf{r}'}\mathcal{H}_{\boldsymbol{Q}}(\mathbf{r},\mathbf{r}')\psi_{\boldsymbol{Q}}(\mathbf{r}') = E\psi_{\boldsymbol{Q}}(\mathbf{r})$. The BSE Hamitonian is therefore:

$$\mathcal{H}_{\boldsymbol{Q}}(\mathbf{r},\mathbf{r}') = \frac{1}{N}\sum_{\mathbf{R}_{\rm cm,1},\mathbf{R}_{\rm cm,2}} e^{i\mathbf{Q}\cdot(\mathbf{R}_{\rm cm,2}-\mathbf{R}_{\rm cm,1})}\langle \mathbf{R}_{\rm cm,1},\mathbf{r}|\hat{H}|\mathbf{R}_{\rm cm,2},\mathbf{r}'\rangle \tag{S3}$$

Kinetic term: the kinetic term $\mathcal{H}_{\mathbf{K}}(\mathbf{r},\mathbf{r}')$ of BSE Hamiltonian is obtained by projecting the kinetic part $\hat{H}_{\mathbf{K}}$ of the total Hamiltonian onto the particle-hole basis, where $\hat{H}_{\mathbf{K}} = \int d\mathbf{x} a^\dagger_{\mathbf{x}}(\frac{\hat{\mathbf{p}}^2}{2m} + V_{\rm lattice}(\mathbf{x}))a_{\mathbf{x}}$. After a straightforward evaluation, we arrive at:

$$\mathcal{H}_{\mathbf{K}}(\mathbf{r},\mathbf{r}') = e^{i\mathbf{Q}\cdot(\mathbf{r}'-\mathbf{r})/2}\langle c,\mathbf{r}|\hat{H}_{\mathbf{K}}|c,\mathbf{r}'\rangle - e^{i\mathbf{Q}\cdot(\mathbf{r}-\mathbf{r}')/2}\langle v,\mathbf{r}|\hat{H}_{\mathbf{K}}|v,\mathbf{r}'\rangle\,, \tag{S4}$$

where $\langle c,\mathbf{r}|\hat{H}_{\mathbf{K}}|c,\mathbf{r}'\rangle \equiv \int d\mathbf{x}\ w^*_{c,\mathbf{r}}(\mathbf{x})(\frac{\hat{\mathbf{p}}^2}{2m} + V_{\rm lattice}(\mathbf{x}))w_{c,\mathbf{r}'}(\mathbf{x})$, which is of order $e^{-|\mathbf{r}-\mathbf{r}'|/\xi_W}$ from the exponential localization nature of Wannier functions (and similarly for v bands).

Utilizing the relation between the Bloch functions and the Wannier functions $w_{\mathbf{R}}(\mathbf{x}) = \frac{1}{\sqrt{N}}\sum\limits_{\mathbf{k}} e^{-i\mathbf{k}\cdot\mathbf{R}}\psi_{\mathbf{k}}(\mathbf{x})$, we can immediately see that the kinetic term $\mathcal{H}_{\mathbf{K}}(\mathbf{r},\mathbf{r}') = \frac{1}{N}\sum\limits_{\mathbf{p}} e^{i\mathbf{p}\cdot(\mathbf{r}-\mathbf{r}')}[\epsilon_c(\mathbf{p}+\mathbf{Q}/2) - \epsilon_v(\mathbf{p}-\mathbf{Q}/2)]$ as discussed in the main text.

Interaction term: the interaction part of the Hamiltonian is of the general form $\hat{H}_{\mathbf{V}} = \int d\mathbf{x}_1 d\mathbf{x}_2 V(|\mathbf{x}_1 - \mathbf{x}_2|)a^\dagger_{\mathbf{x}_1}a^\dagger_{\mathbf{x}_2}a_{\mathbf{x}_2}a_{\mathbf{x}_1}$. The electron-electron interaction $V(|\mathbf{x}|)$ can either be screened or un-screened and vanishes as $|\mathbf{x}|\to\infty$. After projecting onto the particle-hole basis and performing Hartree-Fock contractions, we arrive at two terms, the direct term $\mathcal{V}_D$ and the exchange interaction term $\mathcal{V}_X$.

* justinsong@ntu.edu.sg

The direct interaction term:

$$\mathcal{V}_D(\mathbf{r},\mathbf{r}') = -\sum_{\Delta\mathbf{R}_{\rm cm}} e^{i\mathbf{Q}\cdot(\Delta\mathbf{R}_{\rm cm})}\int d\mathbf{x}_1 d\mathbf{x}_2 w_{c,\Delta\mathbf{R}_{\rm cm}+\mathbf{r}'/2}(\mathbf{x}_1) w^*_{c,\mathbf{r}/2}(\mathbf{x}_1) V(|\mathbf{x}_1-\mathbf{x}_2|) w_{v,-\mathbf{r}/2}(\mathbf{x}_2) w^*_{v,\Delta\mathbf{R}_{\rm cm}-\mathbf{r}'/2}(\mathbf{x}_2), \quad \text{(S5)}$$

where the minus sign comes from electron anti-commutations and $\Delta\mathbf{R}_{\rm cm} = \mathbf{R}_{{\rm cm},2} - \mathbf{R}_{{\rm cm},1}$.

Physically, each coordinate $\mathbf{x}_i$ only takes significant non-zero values near the location of the Wannier functions which makes the $\mathcal{V}_D(\mathbf{r},\mathbf{r}')$ almost diagonal in the relative coordinate space. A simple estimate can be made by noticing that e.g., $w_{n,\mathbf{R}}(\mathbf{x})$ is upper bounded by $e^{-|\mathbf{R}-\mathbf{x}|/\xi_W}$. Therefore we have

$$\begin{aligned} |\mathcal{V}_D(\mathbf{r},\mathbf{r}')| &< e^{-(|\Delta\mathbf{R}_{\rm cm}+\mathbf{r}'/2-\mathbf{x}_1|+|\mathbf{r}/2-\mathbf{x}_1|+|-\mathbf{r}/2-\mathbf{x}_2|+|\Delta\mathbf{R}_{\rm cm}-\mathbf{r}'/2-\mathbf{x}_2|)/\xi_W} \\ &< e^{-(|(\Delta\mathbf{R}_{\rm cm}+\mathbf{r}'/2-\mathbf{x}_1)-(\mathbf{r}/2-\mathbf{x}_1)+(-\mathbf{r}/2-\mathbf{x}_2)-(\Delta\mathbf{R}_{\rm cm}-\mathbf{r}'/2-\mathbf{x}_2)|)/\xi_W} = e^{-(|\mathbf{r}'-\mathbf{r}|)/\xi_W}. \end{aligned} \quad \text{(S6)}$$

This means that $\mathcal{V}_D(\mathbf{r},\mathbf{r}')$ is non-zero only when $\mathbf{r}\sim\mathbf{r}'$ (i.e., nearly diagonal in $\mathbf{r}$).

The exchange interaction is:

$$\mathcal{V}_X(\mathbf{r},\mathbf{r}') = \sum_{\Delta\mathbf{R}_{\rm cm}} e^{i\mathbf{Q}\cdot(\Delta\mathbf{R}_{\rm cm})}\int d\mathbf{x}_1 d\mathbf{x}_2 w_{c,\Delta\mathbf{R}_{\rm cm}+\mathbf{r}'/2}(\mathbf{x}_1) w^*_{v,\Delta\mathbf{R}_{\rm cm}-\mathbf{r}'/2}(\mathbf{x}_1) V(|\mathbf{x}_1-\mathbf{x}_2|) w_{v,-\mathbf{r}/2}(\mathbf{x}_2) w^*_{c,\mathbf{r}/2}(\mathbf{x}_2), \quad \text{(S7)}$$

Since it is the exchange energy of electron and hole, the electron and hole should be close to have non-negligible exchange (meaning that the relative coordinate should be small). For example if we only look at the product of Wannier functions with common coordinate $\mathbf{x}_1$, then it is upper-bounded by:

$$e^{-(|\Delta\mathbf{R}_{cm}+\mathbf{r}'/2-\mathbf{x}_1|+|\Delta\mathbf{R}_{\rm cm}-\mathbf{r}'/2-\mathbf{x}_1|)/\xi_W} < e^{-(|(\Delta\mathbf{R}_{\rm cm}+\mathbf{r}'/2-\mathbf{x}_1)-(\Delta\mathbf{R}_{\rm cm}-\mathbf{r}'/2-\mathbf{x}_1)|)/\xi_W} = e^{-|\mathbf{r}'|/\xi_W}. \quad \text{(S8)}$$

Similarly the product of Wannier functions with common coordinate $\mathbf{x}_2$ is upper-bounded by $e^{-|\mathbf{r}|/\xi_W}$:

$$e^{-(|-\mathbf{r}/2-\mathbf{x}_2|+|\mathbf{r}/2-\mathbf{x}_2|)/\xi_W} < e^{-(|(-\mathbf{r}/2-\mathbf{x}_2)-(\mathbf{r}/2-\mathbf{x}_2)|)/\xi_W} = e^{-|\mathbf{r}|/\xi_W}. \quad \text{(S9)}$$

As a result, the full $\mathcal{V}_X(\mathbf{r},\mathbf{r}')$ is therefore upper bounded by $e^{-(|\mathbf{r}|+|\mathbf{r}'|)/\xi_W}$, meaning that it only concentrates near $|\mathbf{r}|,|\mathbf{r}'|\sim 0$. Furthermore we have $\mathcal{V}_X(\mathbf{r},\mathbf{r}') < e^{-(|\mathbf{r}|+|\mathbf{r}'|)/\xi_W} < e^{-|\mathbf{r}-\mathbf{r}'|/\xi_W}$, meaning $\mathcal{V}_X$ is still almost diagonal.

In summary, we have given the explicit form of Bethe-Salpeter Hamiltonian in relative coordinates as the sum of three terms $\mathcal{H}\equiv\mathcal{H}_\mathbf{K}+\mathcal{V}_D+\mathcal{V}_X$ and verified the exponentially decaying $e^{-|\mathbf{r}-\mathbf{r}'|/\xi_W}$ of the off-diagonal elements $\mathcal{H}(\mathbf{r},\mathbf{r}')$.

II. FLUX INSERTION AND THE TRANSFORMATION RULE OF MATRIX ELEMENTS

Following Kohn[1, 2], we introduce the following parameterized Hamiltonian on a periodic lattice with linear dimension L:

$$\hat{H}(\boldsymbol{\kappa}) = \frac{1}{2m}\sum_{i=1}^{N}(\hat{\mathbf{p}}_i+\hbar\boldsymbol{\kappa})^2 + \hat{V}_{\rm lattice} + \hat{H}_{\rm int}, \quad \text{(S10)}$$

where $\boldsymbol{\kappa}$ is a uniform flux (units inverse length), $\hat{V}_{\rm lattice}$ is the lattice potential and $\hat{H}_{\rm int}$ describes electron-electron interactions. We label the n-th eigen-states of $\hat{H}(\boldsymbol{\kappa})$ as $|\Phi_n(\boldsymbol{\kappa})\rangle$. The vector $\boldsymbol{\kappa}$ can be interpreted as arising from the insertion of a flux through the hole of the cylinder (see Fig. S1); as shown in the figure $\boldsymbol{\kappa}$ points along the circumference of the cylinder. Henceforth, we will refer to this process as flux insertion; this is also referred to as "twisted boundary conditions" in the literature.

From the free part (the sum of kinetic energy and $\hat{V}_{\rm lattice}$), we can solve for the Bloch functions and then Fourier transform them to Wannier functions. Below we will track the change of Wannier functions and the matrix elements of BSE Hamiltonian in the Wannier basis in response to flux insertion. As discussed in Ref. [1, 3], the Wannier function corresponding to the free part after flux insertion is related to that before flux insertion via $w^{\boldsymbol{\kappa}}_{n,\mathbf{R}}(\mathbf{x}) = e^{-i\boldsymbol{\kappa}\cdot(\mathbf{x}-\mathbf{R})}w_{n,\mathbf{R}}(\mathbf{x})$. We shall denote the corresponding creation operator as $c^\dagger_{\boldsymbol{\kappa};n,\mathbf{R}}$.

We now turn to analyzing the Hamiltonian terms under flux insertion. Any general particle-conserving one-body operator $\hat{G}\equiv\int d\mathbf{x} a^\dagger_\mathbf{x} G(\hat{\mathbf{x}},\hat{\mathbf{p}}) a_\mathbf{x}$ can be expressed in the Wannier basis as:

$$\hat{G} = \sum_{n,m,\mathbf{R},\mathbf{R}'}\langle n,\mathbf{R}|\,\hat{G}\,|m,\mathbf{R}'\rangle\, c^\dagger_{n,\mathbf{R}} c_{m,\mathbf{R}'}, \quad \text{(S11)}$$

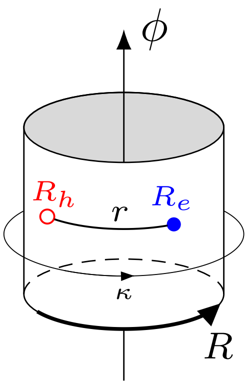

FIG. S1. **A schematic of flux threading.** The system is wrapped around a cylinder with circumference L, with flux ϕ piercing through the hole. $\boldsymbol{R}$ indicates the coordinates of particles. Electrons and holes carry opposite charges, resulting in opposite signs of the phase factor when moving in a background of vector potential $\boldsymbol{\kappa}$. Consequently, only the relative coordinate $\boldsymbol{r} = \boldsymbol{R}_e - \boldsymbol{R}_h$ couples to $\boldsymbol{\kappa}$. Notice that when $\mathbf{r}$ is near $|\mathbf{r} \cdot \boldsymbol{e}_\kappa| = 0 \pmod{L}$, the electron and hole remain close to each other. In contrast, when $\mathbf{r}$ is close to $|\mathbf{r} \cdot \boldsymbol{e}_\kappa| = L/2 \pmod{L}$, they are maximally separated; in the main text we refer to this latter region as the boundary region.

with $\langle n, \mathbf{R}| \hat{G} |m, \mathbf{R}'\rangle \equiv \int d\mathbf{x} w^*_{n,\mathbf{R}}(\mathbf{x}) G(\hat{\mathbf{x}}, \hat{\mathbf{p}}) w_{m,\mathbf{R}'}(\mathbf{x})$.

Upon inserting a uniform flux, the operator becomes: $\hat{G}^{\boldsymbol{\kappa}} = \int d\mathbf{x} a^\dagger_{\mathbf{x}} G(\hat{\mathbf{x}}, \hat{\mathbf{p}} + \hbar\boldsymbol{\kappa}) a_{\mathbf{x}}$, and the matrix elements in the flux-inserted Wannier basis $|\boldsymbol{\kappa}; n, \mathbf{R}\rangle \equiv c^\dagger_{\boldsymbol{\kappa};n,\mathbf{R}} |0\rangle$ are given by:

$$\int d\mathbf{x} w^*_{n,\mathbf{R}}(\mathbf{x}) e^{i\boldsymbol{\kappa}\cdot(\mathbf{x}-\mathbf{R})} G(\hat{\mathbf{x}}, \hat{\mathbf{p}} + \hbar\boldsymbol{\kappa}) e^{-i\boldsymbol{\kappa}\cdot(\mathbf{x}-\mathbf{R}')} w_{m,\mathbf{R}'}(\mathbf{x}) = e^{-i\boldsymbol{\kappa}\cdot(\mathbf{R}-\mathbf{R}')} \int d\mathbf{x} w^*_{n,\mathbf{R}}(\mathbf{x}) G(\hat{\mathbf{x}}, \hat{\mathbf{p}}) w_{m,\mathbf{R}'}(\mathbf{x}), \tag{S12}$$

where we have used $e^{i\boldsymbol{\kappa}\cdot\mathbf{x}} G(\hat{\mathbf{x}}, \hat{\mathbf{p}} + \hbar\boldsymbol{\kappa}) e^{-i\boldsymbol{\kappa}\cdot\mathbf{x}} = G(\hat{\mathbf{x}}, \hat{\mathbf{p}})$ recalling that $(\hat{\mathbf{p}} + \hbar\boldsymbol{\kappa}) e^{-i\boldsymbol{\kappa}\cdot\mathbf{x}} = e^{-i\boldsymbol{\kappa}\cdot\mathbf{x}} \hat{\mathbf{p}}$.

Thus, the full operator after flux insertion becomes (see Fig. S1):

$$\hat{G}^{\boldsymbol{\kappa}} = \sum_{n,m,\mathbf{R},\mathbf{R}'} e^{-i\boldsymbol{\kappa}\cdot(\mathbf{R}-\mathbf{R}')} \langle n, \mathbf{R}| \hat{G} |m, \mathbf{R}'\rangle c^\dagger_{\boldsymbol{\kappa};n,\mathbf{R}} c_{\boldsymbol{\kappa};m,\mathbf{R}'} \tag{S13}$$

Similarly, for any general particle-conserving two-body operator $\hat{F} \equiv \int d\mathbf{x}_1 \int d\mathbf{x}_2 a^\dagger_{\mathbf{x}_1} a^\dagger_{\mathbf{x}_2} F(\hat{\mathbf{x}}_1, \hat{\mathbf{x}}_2) a_{\mathbf{x}_2} a_{\mathbf{x}_1}$, the matrix element $\langle \mathbf{R}_1, \mathbf{R}_2| \hat{F} |\mathbf{R}_3, \mathbf{R}_4\rangle$ in the Wannier basis transforms under flux insertion as:

$$\langle \mathbf{R}_1, \mathbf{R}_2| \hat{F} |\mathbf{R}_3, \mathbf{R}_4\rangle \to e^{-i\boldsymbol{\kappa}\cdot(\mathbf{R}_1+\mathbf{R}_2-\mathbf{R}_3-\mathbf{R}_4)} \langle \mathbf{R}_1, \mathbf{R}_2| \hat{F} |\mathbf{R}_3, \mathbf{R}_4\rangle . \tag{S14}$$

For the BSE Hamiltonian, by directly substituting the flux-threaded Wannier functions into each of its terms, namely, $\mathcal{H}_{\mathbf{K}}$, $\mathcal{V}_D$, $\mathcal{V}_X$, we obtain the following transformation rule of matrix elements $\mathcal{H}(\mathbf{r}, \mathbf{r}')$ under flux insertion:

$$\mathcal{H}^{\boldsymbol{\kappa}}(\mathbf{r}, \mathbf{r}') = e^{-i\boldsymbol{\kappa}\cdot(\mathbf{r}-\mathbf{r}')} \mathcal{H}(\mathbf{r}, \mathbf{r}'). \tag{S15}$$

where we have noted the opposite charge for electrons and holes in the BSE mean that fluxes accumulate through the relative coordinate $\mathbf{r}$.

III. INSENSITIVITY OF EXCITON TRANSITION SHIFT VECTOR VS. $\hat{V}$

In this section, we first prove in general that the exciton transition shift vector is insensitive to the form of an interaction $\hat{V}$ inducing the transition from ground state to exciton state; we then extend it to show that transitions between excitonic states are also insensitive to the form of $\hat{V}$. To that end, we consider excitonic transition shift vectors induced by two general particle-conserving interactions $\hat{V}_1$ and $\hat{V}_2$ and evaluate their differences $\delta\boldsymbol{\mathcal{R}} = \boldsymbol{\mathcal{R}}^{V_1}_{0\to\mathrm{ex}} - \boldsymbol{\mathcal{R}}^{V_2}_{0\to\mathrm{ex}}$.

Any particle-conserving one-body interaction $\hat{V}_1$ can be expanded as $\hat{V}_1 \equiv \sum_{\mathbf{R},\mathbf{R}'} (V_1)^{n,m}_{\mathbf{R},\mathbf{R}'} c^\dagger_{n,\mathbf{R}} c_{m,\mathbf{R}'}$ in the Wannier basis, where n, m are band indices. (The treatment of particle-conserving two-body interaction is exactly the same.) Following the previous discussion, the matrix elements transform as $(V_1)^{n,m}_{\mathbf{R},\mathbf{R}'} \to e^{-i\boldsymbol{\kappa}\cdot(\mathbf{R}-\mathbf{R}')}(V_1)^{n,m}_{\mathbf{R},\mathbf{R}'}$ under flux insertion. The transformation rule holds only for particle-conserving interactions and is violated in particle-non-conserving interactions such as $c^\dagger c^\dagger$, as expected in a superconductor.

Using Wick's theorem, we have $\langle\Phi_0(\boldsymbol{\kappa})|\,\hat{V}_1\,|\Phi_{\rm ex}(\boldsymbol{\kappa})\rangle = \sum_{\mathbf{R},\mathbf{R}'} e^{i\mathbf{Q}\cdot(\mathbf{R}+\mathbf{R}')/2}(V_1)^{v,c}_{\mathbf{R},\mathbf{R}'}\psi_{\mathbf{Q}}(\mathbf{R}'-\mathbf{R})$, which is independent of $\boldsymbol{\kappa}$ (with a deviation $\mathcal{O}(e^{-L/(2\xi_M)})$ from the envelope function) as the phase change of $(V_1)^{v,c}_{\mathbf{R},\mathbf{R}'}$ and $\psi_{\mathbf{Q}}(\mathbf{R}'-\mathbf{R})$ arising from flux insertion exactly cancels. Similarly we can show that $\langle\Phi_{\rm ex}(\boldsymbol{\kappa})|\,\hat{V}_2\,|\Phi_0(\boldsymbol{\kappa})\rangle$ is also independent of $\boldsymbol{\kappa}$ also with a deviation $\mathcal{O}(e^{-L/(2\xi_M)})$. Although each term $\langle\Phi_0(\boldsymbol{\kappa})|\,\hat{V}_1\,|\Phi_{\rm ex}(\boldsymbol{\kappa})\rangle$ and $\langle\Phi_{\rm ex}(\boldsymbol{\kappa})|\,\hat{V}_2\,|\Phi_0(\boldsymbol{\kappa})\rangle$ are both gauge dependent, their product is gauge invariant and is independent of $\boldsymbol{\kappa}$ with a combined deviation $\mathcal{O}(e^{-L/\xi_M})$. Therefore we arrive at Eq. (9) shown in the main text:

$$\boldsymbol{\mathcal{R}}^{V_1}_{0\to{\rm ex}} - \boldsymbol{\mathcal{R}}^{V_2}_{0\to{\rm ex}} = \nabla_{\boldsymbol{\kappa}}\arg(\langle\Phi_0(\boldsymbol{\kappa})|\,\hat{V}_1\,|\Phi_{\rm ex}(\boldsymbol{\kappa})\rangle\,\langle\Phi_{\rm ex}(\boldsymbol{\kappa})|\,\hat{V}_2\,|\Phi_0(\boldsymbol{\kappa})\rangle) = \mathcal{O}(e^{-L/\xi_M}). \tag{S16}$$

Furthermore, we can establish the following relation for the shift vector between two excitons ex$_1$ and ex$_2$:

$$\boldsymbol{\mathcal{R}}^{V_{12}}_{{\rm ex}_1\to{\rm ex}_2} \doteq \boldsymbol{\mathcal{R}}^{V_{02}}_{0\to{\rm ex}_2} - \boldsymbol{\mathcal{R}}^{V_{01}}_{0\to{\rm ex}_1}. \tag{S17}$$

This reduces to showing that $\nabla_{\boldsymbol{\kappa}}\arg(\langle\Phi_0(\boldsymbol{\kappa})|\,\hat{V}_{01}\,|\Phi_{{\rm ex}_1}(\boldsymbol{\kappa})\rangle\,\langle\Phi_{{\rm ex}_1}(\boldsymbol{\kappa})|\,\hat{V}_{12}\,|\Phi_{{\rm ex}_2}(\boldsymbol{\kappa})\rangle\,\langle\Phi_{{\rm ex}_2}(\boldsymbol{\kappa})|\,\hat{V}_{02}\,|\Phi_0(\boldsymbol{\kappa})\rangle) \doteq 0$, which can be demonstrated in the same manner as before, with a deviation of order $\mathcal{O}(e^{-2L/\xi_M})$, arising from the four appearances of the exciton envelope functions in the full Wilson loop.

IV. SYMMETRY PROPERTIES OF SHIFT VECTOR FOR LOCALIZED STATES

In this section, we prove the transformation rule of shift vector between two localized states (e.g., excitonic transitions) Eq. (11) under the spatial symmetries of a system. To this end, we first rewrite the shift vector in terms of a manifestly gauge-invariant Wilson loop operator[4]: $\boldsymbol{\mathcal{R}}_{0\to{\rm ex}}(\boldsymbol{Q}) \equiv \nabla_{\boldsymbol{\kappa}}\arg\mathcal{W}^{\boldsymbol{Q}}(\boldsymbol{\kappa})|_{\boldsymbol{\kappa}=\mathbf{0}}$, where the Wilson loop $\mathcal{W}^{\boldsymbol{Q}}(\boldsymbol{\kappa})$ is:

$$\mathcal{W}^{\boldsymbol{Q}}_{0\to{\rm ex}}(\boldsymbol{\kappa}) = \langle\Phi_0(\mathbf{0})|\Phi_0(\boldsymbol{\kappa})\rangle\,\langle\Phi_0(\boldsymbol{\kappa})|\,\hat{V}\,|\Phi_{{\rm ex},\boldsymbol{Q}}(\boldsymbol{\kappa})\rangle\,\langle\Phi_{{\rm ex},\boldsymbol{Q}}(\boldsymbol{\kappa})|\Phi_{\rm ex}(\mathbf{0})\rangle \tag{S18}$$

This is a direct consequence of the following fact: $\langle\Phi_0(\mathbf{0})|\Phi_0(\boldsymbol{\kappa})\rangle = 1 + \boldsymbol{\kappa}\cdot\langle\Phi_0|\,\nabla_{\boldsymbol{\kappa}}\,|\Phi_0\rangle + \mathcal{O}(\kappa^2)$, which leads to $\lim_{\boldsymbol{\kappa}\to 0}\nabla_{\boldsymbol{\kappa}}\arg(\langle\Phi_0(\mathbf{0})|\Phi_0(\boldsymbol{\kappa})\rangle) = -i\,\langle\Phi_0|\,\nabla_{\boldsymbol{\kappa}}\,|\Phi_0\rangle$ (and similarly for $\Phi_{\rm ex}$). Here we have made explicit the $\boldsymbol{Q}$ dependence of the exciton as it will also change under spatial symmetry.

Let's assume a system has G as its point group symmetry, which may contain rotation C_n, mirror reflections M, other operations and combinations. Under a general point group symmetry operation $g \in G$, the symmetry transformation rule is:

$$g\,|\Phi_{{\rm ex},\boldsymbol{Q}}(\boldsymbol{\kappa})\rangle = e^{i\theta_{\rm ex}(\boldsymbol{\kappa})}\,|\Phi_{{\rm ex},\hat{U}_g\boldsymbol{Q}}(\hat{U}_g\boldsymbol{\kappa})\rangle\,, \tag{S19}$$

where we have included a phase factor $e^{i\theta_{\rm ex}(\boldsymbol{\kappa})}$ to account for symmetry quantum number.

In order to make a connection with the Wilson loop at symmetry-transformed $\boldsymbol{\kappa}' = \hat{U}_g\boldsymbol{\kappa}$, we apply the above relation to the states:

$$\begin{aligned}\mathcal{W}^{\boldsymbol{Q}}_{0\to{\rm ex}}(\boldsymbol{\kappa}) &= \langle\Phi_0(\mathbf{0})|\Phi_0(\boldsymbol{\kappa})\rangle\,\langle\Phi_0(\boldsymbol{\kappa})|\,\hat{V}\,|\Phi_{{\rm ex},\boldsymbol{Q}}(\boldsymbol{\kappa})\rangle\,\langle\Phi_{{\rm ex},\boldsymbol{Q}}(\boldsymbol{\kappa})|\Phi_{{\rm ex},\boldsymbol{Q}}(\mathbf{0})\rangle\,.\\ &= e^{-i\theta_0(\mathbf{0})+i\theta_{\rm ex}(\mathbf{0})}\,\langle\Phi_0(\mathbf{0})|\Phi_0(\boldsymbol{\kappa}')\rangle\,\langle\Phi_0(\boldsymbol{\kappa}')|\,g\hat{V}g^{-1}\,|\Phi_{{\rm ex},\hat{U}_g\boldsymbol{Q}}(\boldsymbol{\kappa}')\rangle\,\langle\Phi_{{\rm ex},\hat{U}_g\boldsymbol{Q}}(\boldsymbol{\kappa}')|\Phi_{{\rm ex},\hat{U}_g\boldsymbol{Q}}(\mathbf{0})\rangle\\ &= e^{-i\theta_0(\mathbf{0})+i\theta_{\rm ex}(\mathbf{0})}\overline{\mathcal{W}}^{\hat{U}_g\boldsymbol{Q}}_{0\to{\rm ex}}(\boldsymbol{\kappa}'),\end{aligned} \tag{S20}$$

where we have used $\langle\Phi_0(\mathbf{0})|\,g^{-1}g\,|\Phi_0(\boldsymbol{\kappa})\rangle = e^{i\theta_0(\boldsymbol{\kappa})-i\theta_0(\mathbf{0})}\,\langle\Phi_0(\mathbf{0})|\Phi_0(\hat{U}_g\boldsymbol{\kappa})\rangle$ and similarly for $|\Phi_{{\rm ex},\boldsymbol{Q}}(\boldsymbol{\kappa})\rangle$, and the Wilson loop $\overline{\mathcal{W}}(\boldsymbol{\kappa}')$ is defined with the symmetry-transformed potential $g\hat{V}g^{-1}$. Notice that $\bar{\mathcal{W}}(\boldsymbol{\kappa}')$ is identical to $\mathcal{W}(\boldsymbol{\kappa}')$ apart from a symmetry-transformed $g\hat{V}g^{-1}$, but the $\hat{V}$-insensitivity of the shift vector is precisely what we have proved in the case of localized states. More precisely, $\arg(\langle\Phi_0(\boldsymbol{\kappa}')|\,\hat{V}_1\,|\Phi_n(\boldsymbol{\kappa}')\rangle\,\langle\Phi_n(\boldsymbol{\kappa}')|\,V_2\,|\Phi_0(\boldsymbol{\kappa}')\rangle)$ is $\boldsymbol{\kappa}'$-independent for $\hat{V}_1 = g\hat{V}g^{-1}$ and $\hat{V}_2 = \hat{V}$. Note that this fact still holds for excitons formed from a Chern band when we represent the

states in terms of hybrid Wannier functions localized along the direction of $\boldsymbol{\kappa}'$-a point we will discuss in Section VI. With these in mind, we have:

$$\nabla_{\boldsymbol{\kappa}'}\arg(\overline{\mathcal{W}}_{0\to\mathrm{ex}}^{\hat{U}_g\boldsymbol{Q}}(\boldsymbol{\kappa}')) \doteq \nabla_{\boldsymbol{\kappa}'}\arg(\mathcal{W}_{0\to\mathrm{ex}}^{\hat{U}_g\boldsymbol{Q}}(\boldsymbol{\kappa}')) \tag{S21}$$

Therefore the shift vector $\boldsymbol{\mathcal{R}}_{0\to\mathrm{ex}}$ can be evaluated with a transformed $\boldsymbol{\kappa}' = \hat{U}_g\boldsymbol{\kappa}$ (noticing that that symmetry quantum numbers $e^{i\theta_{0/\mathrm{ex}}(\mathbf{0})}$ are $\boldsymbol{\kappa}$-independent):

$$\boldsymbol{\mathcal{R}}_{0\to\mathrm{ex}}(\boldsymbol{Q}) = \nabla_{\boldsymbol{\kappa}}\arg\mathcal{W}^{\boldsymbol{Q}}(\boldsymbol{\kappa})|_{\boldsymbol{\kappa}=0} \doteq \nabla_{\boldsymbol{\kappa}}\arg\mathcal{W}^{\hat{U}_g\boldsymbol{Q}}(\boldsymbol{\kappa}')|_{\boldsymbol{\kappa}=0} = \frac{\partial\boldsymbol{\kappa}'}{\partial\boldsymbol{\kappa}}\nabla_{\boldsymbol{\kappa}'}\arg\mathcal{W}^{\hat{U}_g\boldsymbol{Q}}(\boldsymbol{\kappa}')|_{\boldsymbol{\kappa}'=0} = [\hat{U}_g]^{-1}\boldsymbol{\mathcal{R}}_{0\to\mathrm{ex}}(\hat{U}_g\boldsymbol{Q}), \tag{S22}$$

(the last equality is due to $[\frac{\partial\kappa_b'}{\partial\kappa_a}] = [\hat{U}_g]_{ba} = [\hat{U}_g^{-1}]_{ab}$ because $\hat{U}_g$ is an orthogonal matrix). Moving the $\hat{U}_g$ to the left, we obtain Eq. (11) in the main-text:

$$\hat{U}_g[\boldsymbol{\mathcal{R}}_{0\to\mathrm{ex}}(\boldsymbol{Q})] \doteq \boldsymbol{\mathcal{R}}_{0\to\mathrm{ex}}(\hat{U}_g\boldsymbol{Q}). \tag{S23}$$

The expression enforces vectorial symmetry constraints for vertical transitions with $\boldsymbol{Q} = 0$. Consequently, any exciton transition shift vector $\boldsymbol{\mathcal{R}}_{0\to\mathrm{ex}}(\boldsymbol{Q} = 0)$ that is not invariant under the symmetry operation g must vanish. A simple example is the in-plane exciton transition shift vector, which is forced to zero by C_{3z} rotational symmetry. We emphasize that this constraint does not apply to the free-particle transition shift vector $\boldsymbol{\mathcal{R}}_{0\to\mathrm{free}}$.

V. TIME-DEPENDENT PERTURBATION CALCULATION OF THE SHIFT CURRENT IN A MANY-BODY SETTING

A. Non-degenerate case

We note that the many-body shift current expression in terms of a many-body shift vector was first given in Ref. 5. Here in this section, we provide an alternative derivation based on time-dependent perturbation theory in the velocity-gauge. The full many-body Hamiltonian with a time-dependent vector potential $\boldsymbol{A}$ can be written as

$$\hat{H}(\boldsymbol{A}) = \frac{1}{2m}\sum_{i=1}^{N}(\mathbf{p}_i - e\boldsymbol{A})^2 + \hat{V}_{\mathrm{lattice}} + \hat{H}_{\mathrm{int}}, \tag{S24}$$

where $\hat{V}_{\mathrm{lattice}}$ is the lattice potential and H_{int} denotes electron-electron interactions. The full Hamiltonian can be expanded as $\hat{H}(\boldsymbol{A}) = \hat{H}(0) + \hat{\boldsymbol{j}}\cdot\boldsymbol{A}$ and we denote the time-dependent interaction formally as $\hat{\boldsymbol{j}}\cdot\boldsymbol{A} = \hat{V}(t)$. The only thing we need in the following derivation is that $\hat{V}(t) = \hat{V}^{\omega}e^{-i\omega t} + \hat{V}^{-\omega}e^{i\omega t}$ and $\hat{V}^{-\omega} = (\hat{V}^{\omega})^*$. Since our focus is solely on the off-diagonal correction to the density matrix, the diamagnetic contribution to the current can be safely neglected. Below we denote the eigen-states of $\hat{H}(0)$ as $|n\rangle$ ($n = 0, 1, \cdots$), where $n = 0$ is the ground state.

The density matrix evolves under the quantum Liouville equation in the interaction picture:

$$\frac{d\rho_I}{dt} = \frac{i}{\hbar}[\rho_I, \hat{V}_I(t)], \tag{S25}$$

where operators in the interaction picture (with subscript I) and the Schrodinger picture (without subscript) are related via the relation $\hat{O}_I(t) = e^{i\hat{H}(0)/\hbar}\hat{O}(t)e^{-i\hat{H}(0)/\hbar}$. Eq.(S25) can be solved up to second order as:

$$\rho_I(t) = \rho_I^{(0)} + \frac{1}{i\hbar}\int_{-\infty}^{t}[\hat{V}_I(t_1), \rho_I^{(0)}]dt_1 + (\frac{1}{i\hbar})^2\int_{-\infty}^{t}\int_{-\infty}^{t_1}[\hat{V}_I(t_1), [\hat{V}_I(t_2), \rho_I^{(0)}]]dt_2dt_1, \tag{S26}$$

where $\rho^{(0)}$ is the unperturbed equilibrium density matrix and in the special case of zero-temperature is simply $\rho^{(0)} = |0\rangle\langle 0|$ (generalization to finite-temperature is straightforward).

The current is therefore

$$\begin{aligned}\boldsymbol{j}(t) \equiv \mathrm{Tr}(\rho_I(t)\hat{\boldsymbol{j}}_I(t)) &= \mathrm{Tr}(\rho_I^{(0)}\hat{\boldsymbol{j}}_I(t)) + \frac{1}{i\hbar}\int_{-\infty}^{t}\mathrm{Tr}(\rho_I^{(0)}[\hat{\boldsymbol{j}}_I(t), \hat{V}_I(t_1)]) \\ &+ (\frac{1}{i\hbar})^2\int_{-\infty}^{t}\int_{-\infty}^{t_1}\mathrm{Tr}(\rho_I^{(0)}[[\hat{\boldsymbol{j}}_I(t), \hat{V}_I(t_1)], \hat{V}_I(t_2)])dt_2dt_1 + \mathcal{O}(A^3).\end{aligned} \tag{S27}$$

The shift current is a second-order (rectified) DC current that originates from off-diagonal elements of ρ and is extracted by inserting the identity $\mathbb{1} = \sum_n |n\rangle\langle n|$, performing the time integral, and isolating the resonant part via a small imaginary shift in the energy denominator. Focusing on the resonant (absorptive) part produces the shift current:

$$\boldsymbol{j}_{\text{shift}} = i\pi \sum_{n\neq 0} \delta(E_{n0} - \hbar\omega)\Big[\sum_{m\neq 0} \frac{\langle 0|\hat{\boldsymbol{j}}|m\rangle}{E_{m0}} \langle m|\hat{V}^{-\omega}|n\rangle \langle n|\hat{V}^{\omega}|0\rangle - \sum_{m\neq n} \langle 0|\hat{V}^{-\omega}|m\rangle \frac{\langle m|\hat{\boldsymbol{j}}|n\rangle}{E_{nm}} \langle n|\hat{V}^{\omega}|0\rangle \Big] + h.c. \tag{S28}$$

While Eq. (S28) describes the full shift current, it is useful to rewrite it in a form that explicitly contains the shift vector. To see this we next prove the following useful many-body sum rule:

$$\left(\sum_{m\neq 0} \frac{\langle 0|\hat{\boldsymbol{j}}|m\rangle}{E_{m0}} \langle m|\hat{V}|n\rangle + \sum_{m\neq n} \langle 0|\hat{V}|m\rangle \frac{\langle m|\hat{\boldsymbol{j}}|n\rangle}{E_{nm}} \right) = -\frac{e}{\hbar} \langle 0|\hat{V}|n\rangle \cdot \Big[\langle n|\nabla_{\boldsymbol{\kappa}}|n\rangle - \langle 0|\nabla_{\boldsymbol{\kappa}}|0\rangle - \nabla_{\boldsymbol{\kappa}} \ln(\langle 0|\hat{V}|n\rangle) \Big]. \tag{S29}$$

This can be proved by first noticing the identity $\langle m|\hat{\boldsymbol{j}}|n\rangle / E_{nm} = -\frac{e}{\hbar}\langle m|\nabla_{\boldsymbol{\kappa}}|n\rangle$ (obtained from taking derivative with $\boldsymbol{\kappa}$ of the expression $\langle m|H|n\rangle$ and identifying $\hbar\boldsymbol{\kappa}$ with $-e\boldsymbol{A}$). Then we sum over intermediate states $|m\rangle$ via the identity $\sum_m |m\rangle\langle m| = \mathbb{1}$. Finally utilizing the terms $(\nabla_{\boldsymbol{\kappa}}\langle 0|)\hat{V}|n\rangle + \langle 0|\hat{V}(\nabla_{\boldsymbol{\kappa}}|n\rangle) = \nabla_{\boldsymbol{\kappa}}(\langle 0|\hat{V}|n\rangle)$, we arrive at the sum rule Eq. (S29). Note that the contribution $\langle 0|(\nabla_{\boldsymbol{\kappa}}\hat{V})|n\rangle$ vanishes for $n \neq 0$.

From this we can reexpress Eq.(S28) as (taking into consideration the hermitian conjugation part):

$$\boldsymbol{j}_{\text{shift}} = -\frac{2\pi e}{\hbar} \sum_n |\langle n|\hat{V}^{\omega}|0\rangle|^2 \boldsymbol{\mathcal{R}}_{0n} \delta(E_n - E_0 - \hbar\omega) \tag{S30}$$

where $\boldsymbol{\mathcal{R}}_{0n} \equiv i\langle n|\nabla_{\boldsymbol{\kappa}}|n\rangle - i\langle 0|\nabla_{\boldsymbol{\kappa}}|0\rangle + \nabla_{\boldsymbol{\kappa}} \arg(\langle 0|\hat{O}|n\rangle)$.

B. Degenerate case

In the case with degeneracies, we can proceed exactly as before using time-dependent perturbation theory and arrive at the following by excluding summations over degenerate energy levels:

$$\boldsymbol{j}_{\text{shift}} = i\pi \sum_{E_n\neq E_0} \delta(E_{n_i,0} - \hbar\omega)\Big[\sum_{E_m\neq E_0} \frac{\langle 0|\hat{\boldsymbol{j}}|m_l\rangle}{E_{m0}} \langle m_l|\hat{V}^{-\omega}|n_i\rangle \langle n_i|\hat{V}^{\omega}|0\rangle - \sum_{E_m\neq E_n} \langle 0|\hat{V}^{-\omega}|m_l\rangle \frac{\langle m_l|\hat{\boldsymbol{j}}|n_i\rangle}{E_{nm}} \langle n_i|\hat{V}^{\omega}|0\rangle + h.c.\Big], \tag{S31}$$

where we have put back the subscripts indicating states within the degenerate subspace (we have also assumed the ground state has no degeneracy for simplicity, although this restriction can be easily lifted). The sum rule in the degenerate case therefore goes like:

$$\Big(\sum_{E_m\neq E_0} \frac{\langle 0|\hat{\boldsymbol{j}}|m_l\rangle}{E_{m0}} \langle m_l|\hat{V}|n_i\rangle - \sum_{E_m\neq E_n} \langle 0|\hat{V}|m_l\rangle \frac{\langle m_l|\hat{\boldsymbol{j}}|n_i\rangle}{E_{nm}} \Big) \tag{S32}$$

$$= -\frac{e}{\hbar}\Big[-\nabla_{\boldsymbol{\kappa}}(\langle 0|\hat{V}|n_i\rangle) + \sum_p \langle 0|\hat{V}|n_p\rangle \langle n_p|\nabla_{\boldsymbol{\kappa}}|n_i\rangle - \sum_p \langle 0|\nabla_{\boldsymbol{\kappa}}|0\rangle \langle 0|\hat{V}|n_i\rangle\Big]. \tag{S33}$$

From this we arrive at the shift current formula in the degenerate case:

$$\boldsymbol{j}_{\text{shift}} = -\frac{2\pi e}{\hbar} \sum_n \delta(E_{n0} - \hbar\omega) \text{Im}\Big[\sum_i \hat{V}^{\omega}_{n_i,0} \nabla_{\boldsymbol{\kappa}}(\hat{V}^{-\omega}_{0,n_i}) + \sum_i \langle 0|\nabla_{\boldsymbol{\kappa}}|0\rangle \hat{V}^{\omega}_{n_i,0}\hat{V}^{-\omega}_{0,n_i} - \sum_{i,j} \langle n_j|\nabla_{\boldsymbol{\kappa}}|n_i\rangle \hat{V}^{\omega}_{n_i,0}\hat{V}^{-\omega}_{0,n_j}\Big] \tag{S34}$$

In the special case of state n being degenerate excitons, utilizing the fact that flux insertion affects the envelope function as $\psi^{\boldsymbol{\kappa}}(\mathbf{r}) \doteq e^{-i\boldsymbol{\kappa}\cdot\mathbf{r}}\psi(\mathbf{r})$, we arrive at the following simplified formula:

$$\boldsymbol{j}_{\text{shift}} = -\frac{2\pi e}{\hbar} \sum_n \delta(E_{n0} - \hbar\omega)\Big[\sum_{i,j} \hat{V}^{-\omega}_{0,n_i} \hat{V}^{\omega}_{n_j,0} \boldsymbol{\mathcal{R}}_{n_i,n_j}\Big], \tag{S35}$$

with the help of the following intrinsic shift vector in the exciton degenerate subspace:

$$\boldsymbol{\mathcal{R}}_{n_i,n_j} = \sum_{\mathbf{r},\mathbf{r}'} [\psi_{n_i}]^*_{cv}(\mathbf{r})[\psi_{n_j}]_{c'v'}(\mathbf{r}')\left[\mathbf{r}\delta_{\mathbf{r},\mathbf{r}'}\delta_{c,c'}\delta_{v,v'} + e^{i\boldsymbol{Q}\cdot(\mathbf{r}'-\mathbf{r})/2}\delta_{v,v'}\boldsymbol{d}_{c,c'}(\mathbf{r}'-\mathbf{r}) - e^{i\boldsymbol{Q}\cdot(\mathbf{r}-\mathbf{r}')/2}\boldsymbol{d}_{v,v'}(\mathbf{r}'-\mathbf{r})\delta_{c,c'}\right], \tag{S36}$$

where we have made conduction and valence band indices explicit to incorporate the case of multiple conduction/valence bands, and the Berry connection between bands n and m are: $\boldsymbol{d}_{n,m}(\mathbf{R}) \equiv \int d\mathbf{x} w^*_{n,\mathbf{0}}(\mathbf{x})\mathbf{x}w_{m,\boldsymbol{R}}(\mathbf{x})$.

VI. DISCUSSION OF TOPOLOGICAL OBSTRUCTED BANDS

For topologically nontrivial bands, a global smooth gauge for Bloch wavefunctions—and hence maximally localized Wannier functions—is obstructed. However, as we now explain, the previous discussions remain valid if we now use hybrid Wannier functions. We proceed by considering a d-dimensional system and, for convenience, insert flux along the x-direction. We denote the remaining momenta collectively as $\mathbf{k}_\perp$. We then construct hybrid Wannier functions localized along x [6–8]:

$$w_{R_x,\mathbf{k}_\perp}(\mathbf{x}) = \frac{1}{\sqrt{N_x}}\sum_{k_x} e^{i(\mathbf{k}\cdot\mathbf{x}-k_xR_x)}e^{i\phi_\mathbf{k}}u_\mathbf{k}(\mathbf{x}), \tag{S37}$$

where the gauge freedom $e^{i\phi_\mathbf{k}}$ is fixed to ensure localization along x and N_x is the number of unit-cells along x direction.

Hybrid Wannier functions have well-defined charge center along x direction as $\bar{x}(\mathbf{k}_\perp) \equiv \langle w_{R_x,\mathbf{k}_\perp}|\,\hat{x}\,|w_{R_x,\mathbf{k}_\perp}\rangle$ due to their localization properties along x. The key point of topological obstruction is that the Wannier charge center can shift when $\mathbf{k}_\perp$ is varied, resulting in an obstruction to exponentially localized Wannier function along all spatial directions. For example, for a Chern insulator with Chern number C, the Wannier center will shift a net amount of Ca along the x direction when k_y changes by 2π. The point, however, is that this shift is still on the order of lattice constants, and therefore is microscopically small relative to the linear dimension L.

A general particle-hole state in this basis can be described as:

$$|\psi_{\boldsymbol{Q}}\rangle_{\mathrm{p-h}} = \frac{1}{\sqrt{N_x}}\sum_{R_x,r_x,\mathbf{p}_\perp}\psi_{\boldsymbol{Q}}(r_x,\mathbf{p}_\perp)e^{iQ_xR_x}c^\dagger_{c,R_x+r_x/2,\mathbf{p}_\perp+\boldsymbol{Q}_\perp/2}c_{v,R_x-r_x/2,\mathbf{p}_\perp-\boldsymbol{Q}_\perp/2}\,|\mathrm{GS}\rangle\,, \tag{S38}$$

where $c^\dagger_{c,R_x,\mathbf{k}_\perp}|0\rangle = \int d\mathbf{x}w_{c,R_x,\mathbf{k}_\perp}(\mathbf{x})a^\dagger_\mathbf{x}|0\rangle$. Projecting the Hamiltonian $\hat{H}$ onto the particle-hole basis, we obtain a BSE Hamiltonian of the form $\mathcal{H}(r_x,r'_x,\mathbf{p}_\perp,\mathbf{p}'_\perp)$. The evaluation is straightforward but lengthy, therefore we only show some representative matrix elements to illustrate the idea. The kinetic term from conduction band is $e^{iQ_x(r'_x-r_x)/2}\langle c,r_x,\mathbf{p}_\perp+\boldsymbol{Q}_\perp/2|\,\hat{H}_\mathbf{K}\,|c,r'_x,\mathbf{p}_\perp+\boldsymbol{Q}_\perp/2\rangle$. And the direct interaction is:

$$\begin{aligned}\mathcal{V}_D(r_x,r'_x,\mathbf{p}_{\perp,1},\mathbf{p}_{\perp,2}) = &-\sum_{\Delta R_x}e^{iQ_x(\Delta R_x)}\int d\mathbf{x}_1d\mathbf{x}_2w_{c,\Delta R_x+r_{x_2}/2,\mathbf{p}_{\perp,2}+\boldsymbol{Q}_\perp/2}(\mathbf{x}_1)w^*_{c,r_{x_1}/2,\mathbf{p}_{\perp,1}+\boldsymbol{Q}_\perp/2}(\mathbf{x}_1)\\ &\times V(|\mathbf{x}_1-\mathbf{x}_2|)w_{v,-r_{x_1}/2,\mathbf{p}_{\perp,1}-\boldsymbol{Q}_\perp/2}(\mathbf{x}_2)w^*_{v,\Delta R_x-r_{x_2}/2,\mathbf{p}_{\perp,2}-\boldsymbol{Q}_\perp/2}(\mathbf{x}_2).\end{aligned} \tag{S39}$$

Using the property that $|w_{n,R_x,\mathbf{p}_\perp}(\mathbf{x})| < \mathcal{F}(C,a,\xi_W)e^{-|R_x-x|/\xi_W}$ for large separation $|R_x-x|$, we can proceed exactly as before and prove that the off-diagonal elements of the BSE Hamiltonian decays as $e^{-|r_x-r'_x|/\xi_W}$, with ξ_W the wave-function extent along the x direction. The constant factor $\mathcal{F}(C,a,\xi_W)$ accounts for possible shifts of Wannier center when $\mathbf{p}_\perp$ is varied, but does not change the fact that all terms of the BSE become exponentially suppressed for large $|r_x-r'_x|$.

Next we show that the exciton envelope function decays exponentially at large r_x. The asympotic behavior can be argued as follows. At very large r_x, we can ignore the electron-hole interaction and the BSE Hamiltonian reduces to $\epsilon_c(\hat{p}_x,\mathbf{p}_\perp)-\epsilon_v(\hat{p}_x,\mathbf{p}_\perp)$ in the hybrid coordinate $(r_x,\mathbf{p}_\perp)$, which results from the Fourier transformation of $\epsilon_c(\mathbf{p})-\epsilon_v(\mathbf{p})$ along the x direction and can be solved by the ansatz $e^{ip_xr_x}$. Notice that $\epsilon_c(p_x,\mathbf{p}_\perp)-\epsilon_v(p_x,\mathbf{p}_\perp)=E$ can only be satisfied when p_x has imaginary parts because E lies within the gap. Consequently the envelope function at large r_x behaves as $e^{i\mathrm{Re}[p_x]r_x}e^{-\mathrm{Im}[p_x]r_x}$ is exponentially decaying.

As a result, when flux is inserted along the x direction, the Bloch function transforms as $e^{i\mathbf{k}\cdot\mathbf{r}}u_{\mathbf{k}+\kappa_x\mathbf{e}_x}(\mathbf{x})$, leading to the transformation rule of the hybrid Wannier functions: $w_{R_x,\mathbf{k}_\perp}(\mathbf{x}) \to e^{-i\kappa_x(x-R_x)}w_{R_x,\mathbf{k}_\perp}(\mathbf{x})$. Therefore for an arbitrary one-body short-range operator $\hat{o}$ expanded in terms of hybrid Wannier functions $\hat{o} = \sum_{R_x,R'_x,\mathbf{p}_\perp,\mathbf{p}'_\perp} o_{R_x,\mathbf{p}_\perp;R'_x,\mathbf{p}'_\perp}c^\dagger_{R_x,\mathbf{p}_\perp}c_{R'_x,\mathbf{p}'_\perp}$,

the matrix elements transform as $o_{R_x,\mathbf{p}_\perp;R'_x,\mathbf{p}'_\perp} \to o_{R_x,\mathbf{p}_\perp;R'_x,\mathbf{p}'_\perp} e^{-i\kappa_x\cdot(R_x-R'_x)}$ upon flux insertion. Similarly, the matrix elements of the BSE Hamiltonian transforms as: $\mathcal{H}(r_x, r'_x, \mathbf{p}_\perp, \mathbf{p}'_\perp) \to e^{-i\kappa_x(r_x-r'_x)}\mathcal{H}(r_x, r'_x, \mathbf{p}_\perp, \mathbf{p}'_\perp)$.

In conclusion, all the essential ingredients for our proof still hold, namely:

1. The BSE Hamiltonian $\mathcal{H}(r_x, r'_x, \mathbf{p}_\perp, \mathbf{p}'_\perp)$ is almost diagonal in r_x, r'_x, meaning that the off-diagonal matrix elements decay as $e^{-|r_x-r'_x|/\xi_W}$.

2. The exciton solution $\psi_{\mathbf{Q}}(r_x, \mathbf{p}_\perp)$ to the BSE is exponentially localized in r_x.

3. Coupling to κ_x is achieved as $\mathcal{H}(r_x, r'_x, \mathbf{p}_\perp, \mathbf{p}'_\perp) \to e^{-i\kappa_x(r_x-r'_x)}\mathcal{H}(r_x, r'_x, \mathbf{p}_\perp, \mathbf{p}'_\perp)$.

With the above preparation, we can now proceed to put the BSE Hamiltonian on a periodic lattice with linear size L, adopt the ansatz $\psi_{\boldsymbol{Q}}(r_x, \mathbf{p}_\perp) \to e^{-i\kappa_x r_x}\psi_{\boldsymbol{Q}}(r_x, \mathbf{p}_\perp)$ and arrive at the conclusion that the ansatz is accurate with an exponentially suppressed deviation $(N/N_x)e^{-L_x/(2\xi_M)}$, where N/N_x comes from summation over $\mathbf{p}_\perp$. In the thermodynamic limit, the exponential factor always dominates over N/N_x.

Simlar to that described in the main text, we can then utilize the transformation rule of the exciton envelope function (along x) and the matrix elements of the operators $\hat{V}_1$ and $\hat{V}_2$ under flux insertion, we can proceed exactly as before and prove that $\arg(\langle\Phi_0(\boldsymbol{\kappa})|\,\hat{V}_1(\boldsymbol{\kappa})\,|\Phi_n(\boldsymbol{\kappa})\rangle\,\langle\Phi_n(\boldsymbol{\kappa})|\,\hat{V}_2(\kappa)\,|\Phi_0(\boldsymbol{\kappa})\rangle)$ is $\boldsymbol{\kappa}$-independent for any $\hat{V}_1$ and $\hat{V}_2$.

Different basis choices yield the same eigenstate up to a global phase, allowing us to choose hybrid Wannier functions localized along any direction. This freedom lets us extend the proof of the excitonic transition shift vector's transformation rule Eq. (11) to Chern-band excitons without modification.

VII. DETAILS OF NUMERICAL SIMULATION

We consider a model on a honeycomb lattice with sublattices A and B in a unit-cell. The real space periodicity vectors are chosen as $\mathbf{a}_1 = (1, 0)$, $\mathbf{a}_2 = (\frac{1}{2}, \frac{\sqrt{3}}{2})$ and $\mathbf{d}_1 \equiv \frac{\mathbf{a}_1 - 2\mathbf{a}_2}{3}$ is the spatial displacement between sites A and B in a unit-cell. For simplicity lattice constant is chosen to be 1. The reciprocal vectors are chosen as $\mathbf{b}_1 = 2\pi(1, -\frac{1}{\sqrt{3}})$, $\mathbf{b}_2 = 2\pi(0, \frac{2}{\sqrt{3}})$. ,

The tight-binding Hamiltonian is given by:

$$H = \sum_{j,l=1,2,3} -t_l c^\dagger_{A,\mathbf{R}_j} c_{B,\mathbf{R}_j+\vec{\delta}_l} + h.c. + \frac{\Delta}{2}(n_{A,\mathbf{R}_j} - n_{B,\mathbf{R}_j}), \tag{S40}$$

where $\mathbf{R}_j = n_1\mathbf{a}_1 + n_2\mathbf{a}_2$ runs through all the unit-cells, $\vec{\delta}_1 = \mathbf{0}$, $\vec{\delta}_2 = \mathbf{a}_2$, $\vec{\delta}_3 = -\mathbf{a}_1 + \mathbf{a}_2$, and $n_{A/B,\mathbf{R}_j} = c^\dagger_{A/B,\mathbf{R}_j} c_{A/B,\mathbf{R}_j}$. Fourier transformation is defined as $c^{A(B)}_{\mathbf{k}} = \frac{1}{\sqrt{N}}\sum_j e^{-i\mathbf{k}\cdot\mathbf{R}_j} c_{A(B),\mathbf{R}_j}$. After Fourier transformation, we have:

$$H(\mathbf{k}) = h_x(\mathbf{k})\sigma_x + h_y(\mathbf{k})\sigma_y + h_z(\mathbf{k})\sigma_z, \tag{S41}$$

where: $h_x(\mathbf{k}) = -t_1 - t_2\cos(\alpha) - t_3\cos(\beta)$, $h_y(\mathbf{k}) = t_2\sin(\alpha) + t_3\sin(\beta)$ and $h_z(\mathbf{k}) = \frac{\Delta}{2}$ and $\alpha = \mathbf{k}\cdot\mathbf{a}_2$, $\beta = \mathbf{k}\cdot(\mathbf{a}_2 - \mathbf{a}_1)$.

The non-interacting Hamiltonian can be readily diagonalized by:

$$U = \sqrt{\frac{h + h_z}{2h}} \begin{pmatrix} 1 & -\frac{h_x - ih_y}{h + h_z} \\ \frac{h_x + ih_y}{h + h_z} & 1 \end{pmatrix}, \tag{S42}$$

with $U^\dagger H U = \begin{pmatrix} h & 0 \\ 0 & -h \end{pmatrix}$ where $h = \sqrt{h_x^2 + h_y^2 + h_z^2}$ and the dependence on $\mathbf{k}$ is suppressed.

We consider screened Coulomb interaction that takes a Keldysh form[9, 10] $V_{\text{Keldysh}}(\mathbf{R}) = U_{\text{int}}[H_0(R/r_0) - Y_0(R/r_0)]$ (a cutoff is introduced for $\mathbf{R} = \mathbf{0}$ with $V_{\text{Keldysh}}(\mathbf{0}) \equiv U_{\text{int}}[H_0(1/r_0) - Y_0(1/r_0)]$), where H_0 and Y_0 are the Struve function and the Bessel function of the second kind, U_{int} denotes the interaction strength and r_0 denotes the screening length. Because different sublattices have different spatial locations, the screened Coulomb interaction is measured using the actual distance between orbitals. Consequently, the Coulomb interaction is represented as:

$$\hat{V}_{\text{Coulomb}} = \sum_{i,j} \mathbf{\Psi}_i^\dagger\,\mathbf{V}(\mathbf{R}_j - \mathbf{R}_i)\,\mathbf{\Psi}_j, \tag{S43}$$

where the orbital annihilation operator in each unit cell is $\mathbf{\Psi}_j = \begin{pmatrix} c_{A,\mathbf{R}_j} \\ c_{B,\mathbf{R}_j} \end{pmatrix}$, and the interaction matrix is defined as:

$$\mathbf{V}(\mathbf{R}) = \begin{pmatrix} V_{\text{Keldysh}}(\mathbf{R}) & V_{\text{Keldysh}}(\mathbf{R}+\mathbf{d}_1) \\ V_{\text{Keldysh}}(\mathbf{R}-\mathbf{d}_1) & V_{\text{Keldysh}}(\mathbf{R}) \end{pmatrix}. \tag{S44}$$

We emphasize that this procedure is the physically correct way to account for the embedding of Bloch functions and only then does the interaction potential respect the lattice symmetry.

For simplicity in the ensuing numerical simulation, we only include the direct interaction. The BSE in the momentum space is:

$$\langle \mathbf{p}, \boldsymbol{Q}| \hat{H} |\mathbf{p}', \boldsymbol{Q}\rangle = \delta_{\mathbf{p},\mathbf{p}'}\big(\epsilon_c(\mathbf{p}+\boldsymbol{Q}/2) - \epsilon_v(\mathbf{p}-\boldsymbol{Q}/2)\big) - \mathcal{V}_D(\mathbf{p},\mathbf{p}',\boldsymbol{Q}), \tag{S45}$$

where the direct interaction is given by Fourier transforming $\hat{V}_{\text{Coulomb}}$ to the momentum space and projecting onto the band basis.

The flux-inserted Hamiltonian is given as follows. Notice that sublattice A and B are located at different spatial positions, the basis vector $(c_{A,\mathbf{R}}, c_{B,\mathbf{R}})$ picks up a phase factor after flux $\boldsymbol{\kappa}$ is inserted and becomes $(e^{i\boldsymbol{\kappa}\cdot\mathbf{R}}c_{A,\mathbf{R}}, e^{i\boldsymbol{\kappa}\cdot(\mathbf{R}+\mathbf{d}_1)}c_{B,\mathbf{R}})$. Consequently the flux-inserted Hamiltonian is:

$$H^{\boldsymbol{\kappa}}(\mathbf{k}) = \mathcal{U}_{\boldsymbol{\kappa}} H(\mathbf{k}+\boldsymbol{\kappa})\mathcal{U}_{\boldsymbol{\kappa}}^\dagger, \tag{S46}$$

where $\mathcal{U}_{\boldsymbol{\kappa}} \equiv \begin{pmatrix} 1 & 0 \\ 0 & e^{-i\boldsymbol{\kappa}\cdot\mathbf{d}_1} \end{pmatrix}$. The flux-inserted Hamiltonian can be diagonalized by $\mathcal{U}_{\boldsymbol{\kappa}} U(\mathbf{k}+\boldsymbol{\kappa})$.

The current operator in the momentum space is defined as $\hat{\boldsymbol{v}}(\boldsymbol{\kappa}) \equiv \frac{\partial H^{\boldsymbol{\kappa}}}{\partial \boldsymbol{\kappa}}$. When projected onto the band basis, it yields the matrix elements:

$$\begin{pmatrix} \boldsymbol{v}_{cc} & \boldsymbol{v}_{cv} \\ \boldsymbol{v}_{vc} & \boldsymbol{v}_{vv} \end{pmatrix} \equiv [U^\dagger(\mathbf{k}+\boldsymbol{\kappa})\mathcal{U}_{-\boldsymbol{\kappa}}](\partial_{\boldsymbol{\kappa}} H^{\boldsymbol{\kappa}}(\mathbf{k}))[\mathcal{U}_{\boldsymbol{\kappa}} U(\mathbf{k}+\boldsymbol{\kappa})]. \tag{S47}$$

The matrix element corresponding to the optical transition between the ground state $|\Phi_0\rangle$ and an excited state $|\Phi_n\rangle$ can therefore be evaluated as:

$$\langle \Phi_0| \boldsymbol{v} |\Phi_n\rangle = \frac{1}{\sqrt{N}} \sum_{\mathbf{k}} \psi(\mathbf{k}) \boldsymbol{v}_{vc}(\mathbf{k}). \tag{S48}$$

In order to break C_{3z} symmetry and arrive at a non-zero excitonic transition shift vector, we choose the parameters $t_2 = t_3 = 0.5\Delta$, $t_1 = 0.45\Delta$, $U_{\text{int}} = 0.75\Delta$ and $r_0 = 1.5$. Flux is inserted along $\mathbf{b}_1$ direction. The Wilson loop is defined as $\mathcal{W} = \langle \Phi_0| \hat{V}_1 |\Phi_n\rangle \langle \Phi_n| \hat{V}_2 |\Phi_0\rangle$ with $\hat{V}_1 = (\sqrt{3}\hat{v}_x + \hat{v}_y)/2$ and $\hat{V}_2 = (\hat{v}_x - \sqrt{3}\hat{v}_y)/2$.

For each system size L, the standard deviation of $\arg(\mathcal{W}_{\boldsymbol{\kappa}})$ is evaluated over $\kappa L/2\pi$ values ranging from 0.01 to 0.91 with step size 0.1. The offset 0.01 is added to avoid possible degeneracies at $\kappa L = 0, \pi$ arising from Kramers degeneracy[11]. The data in Fig. (2) (a,b) in the main text are simulated on a lattice with $\sqrt{N} = 24$. In producing the scaling with $\sqrt{N}$ in Fig. (2)(a,c), we have taken $\sqrt{N}$ ranging from 12 to 42 with steps of 6. Because the number of excitons grow with system sizes, we order the delocalized states above the gap Δ by energy and then select the same ordinal state (e.g., the tenth) across different sizes in plotting Fig. (2).

VIII. NUMERICAL DETAILS OF MOS_2 CALCULATION AND COMPARISON BETWEEN REAL-SPACE FORMULA AND MOMENTUM SPACE FORMULA

We adopt the three-band nearest-neighbor MoS_2 tight-binding model in Ref.[12]. The model characterizes Mo-$|d_{z^2}\rangle, |d_{xy}\rangle, |d_{x^2-y^2}\rangle$ orbitals with on-site spin-orbital coupling to account for spin-valley locking. After projecting the on-site spin orbital coupling onto this three-d-orbital space, only the term $\lambda L_z S_z$ ($S_z = \pm 1/2$ in this convention), with:

$$L_z = \begin{bmatrix} 0 & 0 & 0 \\ 0 & 0 & 2i \\ 0 & -2i & 0 \end{bmatrix}. \tag{S49}$$

We have used the SOC value $\lambda = 0.073eV$ for MoS_2 according to Ref.[12]. And the lattice constant is taken to be $a = 3.16$Å. Due to the conserved S_z in the three-band model, exciton states can be labeled by spins. Note that

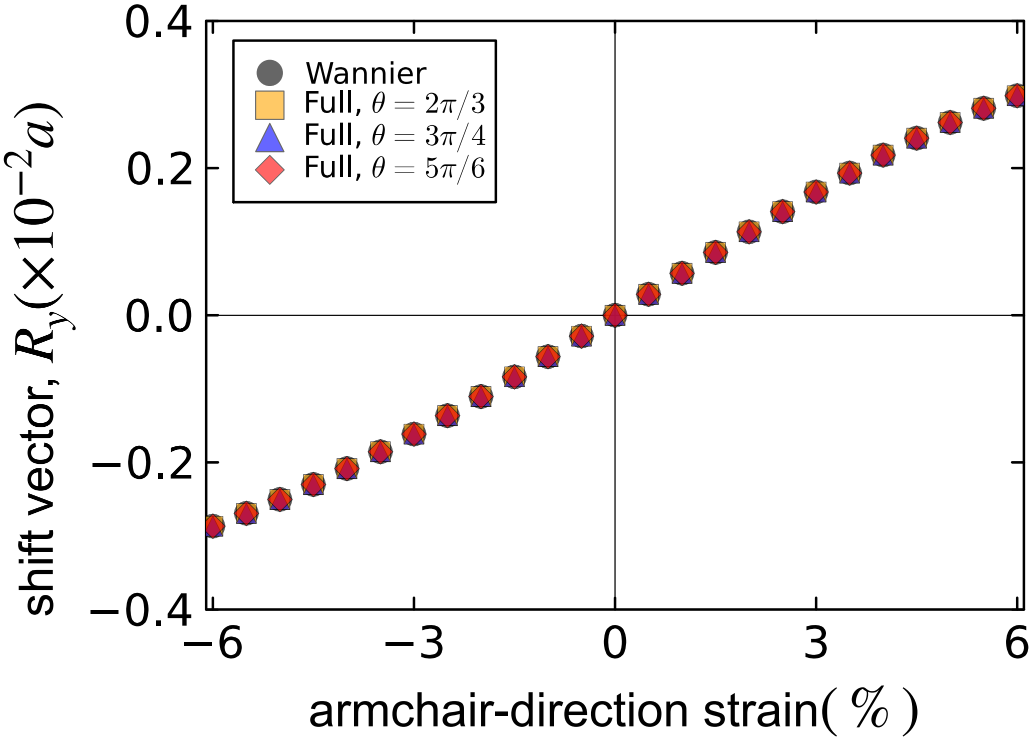

FIG. S2. **Light-polarization independence of the excitonic transition shift vector.** We numerically compute the shift vector of the K-valley $A1s$ exciton in a three-band model of MoS_2 using two formulations. (Method 1) The full expression [Eq. (7) of the main text], which includes the $\boldsymbol{\kappa}$ derivative and explicit dependence on the light-polarization angle θ, is used to compute the armchair(y)-direction shift vector. The resulting shift vectors as functions of armchair-direction strain are independent of θ and vanish ($R_y = 0$) in the absence of strain. (Method 2) The same quantity is computed using the Wannier-based formula [Eq. (10) of the main text] involving only Wannier functions and the exciton envelope function, and is manifestly independent of light-polarization. The two methods agree quantitatively: the standard deviations between the Wannier-based and full results for the three different light polarizations are all below $5 \times 10^{-7}a$.

for energy-degenerate excitons in the unstrained case, opposite S_z corresponds to excitons in opposite valleys, since the time-reversal symmetry relating them reverses both momentum and spin. To simulate the armchair-direction strain[13], we modify the bond length along the y direction by a strain $\epsilon_y \equiv \frac{|r_y - r_{0y}|}{|r_{0y}|}$ and modify the hopping strength according to $t(\mathbf{r}) = t(\mathbf{r}_0)(1 - \Lambda\frac{|\mathbf{r}-\mathbf{r}_0|}{|\mathbf{r}_0|})$, where $\Lambda = -\frac{d\ \ln\ t}{d\ \ln\ r}$ is taken to be 5, following Ref.[14].

The Coulomb interaction is of the Keldysh form $V_R = \frac{\pi e^2}{2\epsilon r_0}[H_0(R/r_0) - Y_0(R/r_0)]$ as adopted in Ref. [10]. Following Ref.[15], we have used the following parameters: $r_0 = 33.875\text{Å}/\epsilon$ and $\epsilon = 2.5$; the on-site interaction is regularized as $V_0 = V_{R=a}$. At each strain ϵ_y, we compute Bloch eigenstates on a 60×60 $\mathbf{k}$ grid. We then generate maximally localized Wannier functions [16] using a workflow that involves: (1) gauge fixing of initial states via local trial orbitals $|g_v\rangle, |g_{c_1}\rangle, |g_{c_2}\rangle$, and (2) minimize the functional of Wannier spreading via unitary rotations of Bloch states on the $\mathbf{k}$ grid, using a gradient descent algorithm until the minimum is achieved. All steps are implemented in our tight-binding calculation. This procedure ensures the Bloch functions have a smooth gauge, which will be crucial in calculating shift vector later. With the gauge-fixed Bloch functions we then solve for the exciton envelope function. Now we have two methods to calculate the excitonic transition shift vectors:

1. Perform a calculation of the shift vector based on the full expression Eq. (7) with explicit knowledge of light-matter interaction. This involves solving the BSE multiple times.

2. Perform a direct evaluation of the many-body shift vector using the Wannier-based expression: Eq. (10), which is efficient as the BSE only needs to be constructed and solved once.

Both methods are used to calculate the excitonic transition shift vectors as summarized in Fig. S2. They agree very well quantitatively. Details of implementation are described as follows.

Method 1: The full formula is evaluated as follows. The $\boldsymbol{\kappa}$-dependence enters the BSE through the Bloch functions: $|u_{c/v,\mathbf{k}}\rangle \to |u_{c/v,\mathbf{k}+\boldsymbol{\kappa}}\rangle$. In practice, we use the interpolation scheme to obtain these Bloch functions at finite $\boldsymbol{\kappa}$ based on the MLWFs previously constructed: $u_{c/v}(\mathbf{k}+\boldsymbol{\kappa}) = \sum_{\mathbf{R}} e^{-i(\mathbf{k}+\boldsymbol{\kappa})\cdot\mathbf{R}} w_{\mathbf{0}}(\mathbf{R})$, where $w_{\mathbf{0}}$ is the Wannier function localized at the origin, with $\mathbf{R}$ shifted accordingly to be centered around the origin. The flux-inserted Hamiltonian is then projected onto the particle-hole basis formed by these flux-inserted Bloch functions to construct the flux-inserted BSE kernel, from which we solve the flux-inserted envelope functions $\psi^{\boldsymbol{\kappa}}(\mathbf{k})$. The explicit form of the full formula is:

$$\begin{aligned}\boldsymbol{\mathcal{R}}_{0n} = i\sum_{\mathbf{k},i}[\psi^{\boldsymbol{\kappa}}_{c_iv}(\mathbf{k})]^*\nabla_{\boldsymbol{\kappa}}[\psi^{\boldsymbol{\kappa}}_{c_iv}(\mathbf{k})] + \sum_{\mathbf{k},i,j}\psi^*_{c_iv}(\mathbf{k})\psi_{c_jv}(\mathbf{k}) \\ \times(\mathcal{A}_{c_ic_j}(\mathbf{k}) - \delta_{i,j}\mathcal{A}_v(\mathbf{k})) + \nabla_{\boldsymbol{\kappa}}\arg[\sum_{\mathbf{k},i} v^{\boldsymbol{\kappa}}_{vc_i}(\mathbf{k})\psi^{\boldsymbol{\kappa}}_{c_iv}(\mathbf{k})],\end{aligned} \tag{S50}$$

where c_i labels the two conduction bands c_1, c_2, and $\mathcal{A}_{c_ic_j}(\mathbf{k}) = i\langle u_{c_i,\mathbf{k}}|\nabla_{\mathbf{k}}u_{c_j,\mathbf{k}}\rangle$ and $\mathcal{A}_v(\mathbf{k}) = i\langle u_{v,\mathbf{k}}|\nabla_{\mathbf{k}}u_{v,\mathbf{k}}\rangle$ are the Berry connections. In calculating the shift vector, we approximated the derivative with respect to $\boldsymbol{\kappa}$ using a finite difference formula $\partial_{\boldsymbol{\kappa}}f|_{\boldsymbol{\kappa}=\mathbf{0}} = \frac{f(\delta\boldsymbol{\kappa})-f(-\delta\boldsymbol{\kappa})}{2|\delta\boldsymbol{\kappa}|} + \mathcal{O}(|\delta\boldsymbol{\kappa}|^2)$ with a small step size $|\delta\boldsymbol{\kappa}| = 0.01|b_1|/\sqrt{N}$, where b_1 is a reciprocal lattice vector.

Method 2: The Wannier-based formula is particularly straightforward. To evaluate Eq. (10), one needs only the MLWFs and the exciton envelope function; consequently, the BSE needs to be solved only once, at $\boldsymbol{\kappa} = 0$. A further advantage of Eq. (10) is that both the envelope function and the MLWFs decay exponentially at large distances. In practice, it is therefore sufficient to retain only terms with $|\mathbf{r}|, |\mathbf{r}'|$ within a few lattice constants. In our calculation, however, we have retained terms with large values of $|\mathbf{r}|, |\mathbf{r}'|$ to ensure convergence.

All our exciton calculations are done on a $N = 60\times 60$ system with periodic boundary conditions. It can be seen that the full formula Eq. (7), evaluated with different light polarizations, yields results that are independent of light-polarization and agree with the Wannier-based formula (Fig. S2). As a measure of accuracy, the root-mean-square deviation (defined as $\sqrt{\frac{1}{n}\sum_i(y_i - y'_i)^2}$) between these results are all below $5\times 10^{-7}a$, confirming that the excitonic transition shift vector is independent of light polarization and enabling the practical use of the more efficient Wannier-based formula Eq. (10). In calculating the shift current, we have regularized the Dirac δ function by $\delta(E-\hbar\omega) = \frac{\Gamma}{\pi}\frac{1}{(E-\hbar\omega)^2+\Gamma^2}$ with $\Gamma = 20meV$ to account for the spectral broadening.

IX. FREE PARTICLE-HOLE SHIFT VECTORS DO NOT TRANSFORM AS A VECTOR

To illustrate that free particle-hole (p-h) shift vectors do not transform as a vector under fixed light polarization, we consider the shift vector for a free p-h optical transition at the C_{3z}-invariant momentum K of unstrained MoS_2, which we denote as $\boldsymbol{\mathcal{R}}_K(\hat{e}_\theta)$, where $\hat{e}_\theta$ denotes the unit vector with light-polarization angle θ. It satisfies the following transformation rule under point group symmetry C_{3z}:

$$\boldsymbol{\mathcal{R}}_K(\hat{e}_\theta) = \hat{U}_{C_{3z}}\left[\boldsymbol{\mathcal{R}}_K(\hat{e}_{\theta+2\pi/3})\right]. \tag{S51}$$

This means that the symmetry acts simultaneously on both the spatial components of the shift vector and the light polarization (Fig. S3). At a fixed polarization θ, the shift vector is not constrained by C_{3z}:

$$\boldsymbol{\mathcal{R}}_K(\hat{e}_\theta) \neq \hat{U}_{C_{3z}}\left[\boldsymbol{\mathcal{R}}_K(\hat{e}_\theta)\right], \tag{S52}$$

therefore it does not transform as a vector at a fixed θ and remains generally nonzero, producing a finite shift current from delocalized p-h excitations. This response can be probed by photons with energies just above the band gap. This behavior contrasts with excitons, where C_{3z} enforces a vanishing excitonic transition shift vector at fixed polarization and an identically zero excitonic transition shift current.

X. FURTHER DETAILS ON MANY-BODY QUANTUM GEOMETRY

In this section, we provide further details on the many-body quantum geometry of resonant optical transitions. Building on the main text, we first derive the explicit form of the parallel-transported tangent vector for a general many-body transition. The derivative reads:

$$\partial_{\boldsymbol{\kappa}}[|\Phi_0(\boldsymbol{\kappa})\rangle\, r^a_{0n}(\boldsymbol{\kappa})\,\langle\Phi_n(\boldsymbol{\kappa})|] = |\partial_{\boldsymbol{\kappa}}\Phi_0(\boldsymbol{\kappa})\rangle\, r^a_{0n}\,\langle\Phi_n(\boldsymbol{\kappa})| + |\Phi_0(\boldsymbol{\kappa})\rangle\,\partial_{\boldsymbol{\kappa}}(r^a_{0n})\,\langle\Phi_n(\boldsymbol{\kappa})| + |\Phi_0(\boldsymbol{\kappa})\rangle\, r^a_{0n}\,\langle\partial_{\boldsymbol{\kappa}}\Phi_n(\boldsymbol{\kappa})|\,. \tag{S53}$$

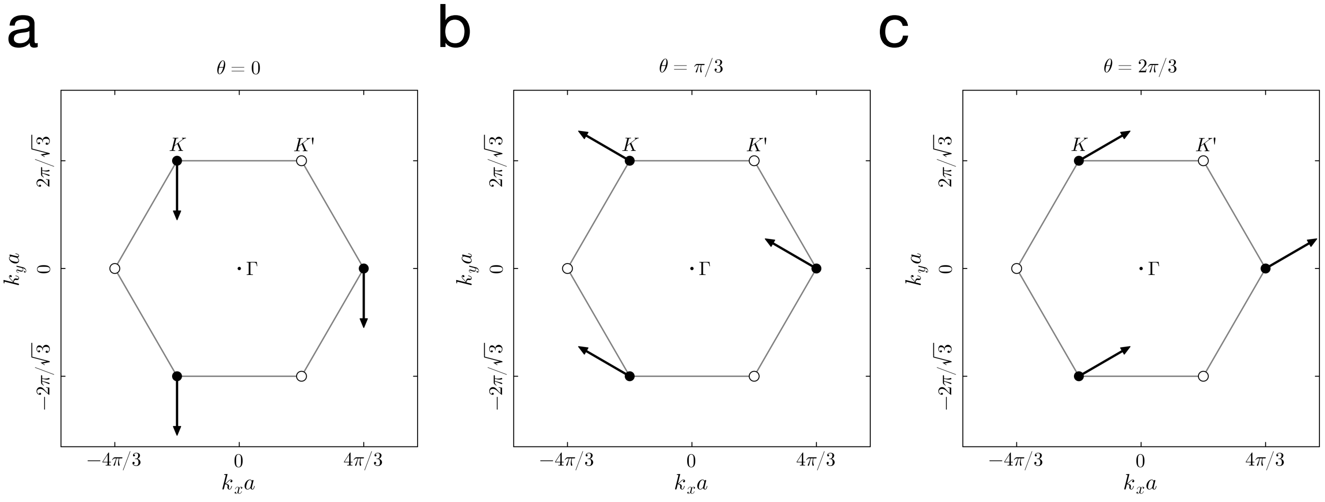

FIG. S3. **Free particle-hole pair shift vector in a three-band tight-binding model of unstrained MoS_2.** (a) Arrows indicate shift vectors evaluated at the three C_{3z}-equivalent K points for light polarization $\theta = 0$. At this fixed light polarization, the shift vector does not transform as a vector under C_{3z} symmetry and is therefore not constrained to vanish. (b) Same setup as in (a), but with the light-polarization angle $\theta = \pi/3$. In this case, shift vectors are related to those at $\theta = 0$ by $\boldsymbol{R}_K(\hat{e}_{\theta=\pi/3}) = [\hat{U}_{C_{3z}}]^2 \boldsymbol{R}_K(\hat{e}_{\theta=0})$. (c) Same setup as in (a), but with the light-polarization angle $\theta = 2\pi/3$. The shift vectors are related to those at $\theta = 0$ by $\boldsymbol{R}_K(\hat{e}_{\theta=2\pi/3}) = \hat{U}_{C_{3z}} \boldsymbol{R}_K(\hat{e}_{\theta=0})$.

After projecting onto the tangent plane, we obtain:

$$\begin{aligned}\nabla_{\boldsymbol{\kappa}}[|\Phi_0(\boldsymbol{\kappa})\rangle\, r^a_{0n}(\boldsymbol{\kappa})\, \langle\Phi_n(\boldsymbol{\kappa})|] &= |\Phi_0(\boldsymbol{\kappa})\rangle\, \langle\Phi_n(\boldsymbol{\kappa})|\, [(\langle\Phi_0|\partial_{\boldsymbol{\kappa}}\Phi_0\rangle + \langle\partial_{\boldsymbol{\kappa}}\Phi_n|\Phi_n\rangle) r^a_{0n} + \partial_{\boldsymbol{\kappa}}(r^a_{0n})] \\ &= |\Phi_0(\boldsymbol{\kappa})\rangle\, \langle\Phi_n(\boldsymbol{\kappa})|\, r^a_{0n}[\partial_{\boldsymbol{\kappa}}(\ln r^a_{0n}) + i\boldsymbol{\mathcal{A}}_n - i\boldsymbol{\mathcal{A}}_0] = \hat{e}^{0n}_a D_{\boldsymbol{\kappa}} \ln(r^a_{0n}),\end{aligned} \tag{S54}$$

with $D_{\kappa_c}\ln(r^a_{0n}) = \partial_{\kappa_c}\ln(r^a_{0n}) + i(\mathcal{A}^c_n - \mathcal{A}^c_0))$. For excitons, following our discussion in the main text, the prefactor $D_{\boldsymbol{\kappa}}\ln(r^a_{0n_{\rm ex}})$ is precisely i times the excitonic transition shift vector $\boldsymbol{\mathcal{R}}_{0n_{\rm ex}}$ which does not depend on light-polarization direction a. The covariant derivative then becomes:

$$\nabla_{\boldsymbol{\kappa}}(\hat{e}^{0n_{\rm ex}}_a) = i\boldsymbol{\mathcal{R}}_{0n_{\rm ex}}\hat{e}^{0n_{\rm ex}}_a. \tag{S55}$$

This factorized form preserves the inner product of tangent vectors under parallel transport, or equivalently, the covariant derivative of the Hermitian metric vanishes:

$$\nabla_{\kappa_c} Q^{0n_{\rm ex}}_{ba} = (\nabla_{\kappa_c}(\hat{e}^{0n_{\rm ex}}_b), \hat{e}^{0n_{\rm ex}}_a) + (\hat{e}^{0n_{\rm ex}}_b, \nabla_{\kappa_c}(\hat{e}^{0n_{\rm ex}}_a)) = -i\mathcal{R}^c_{0n_{\rm ex}} Q^{0n_{\rm ex}}_{ba} + i\mathcal{R}^c_{0n_{\rm ex}} Q^{0n_{\rm ex}}_{ba} = 0, \tag{S56}$$

where we have used the property of the Hilbert-Schmidt inner product $(A, B) = \mathrm{Tr}[A^\dagger B]$.

Consequently, parallel transport of exciton transition tangent vectors is both norm- and angle-preserving. To see this, notice that both the norm of a tangent vector and the angle between two tangent vectors are determined by the Hermitian metric: the norm $||\hat{e}^{0n_{\rm ex}}_a|| \equiv \sqrt{(\hat{e}^{0n_{\rm ex}}_a, \hat{e}^{0n_{\rm ex}}_a)} = \sqrt{Q^{0n_{\rm ex}}_{aa}}$, and the angle $\cos(\theta^{0n_{\rm ex}}_{ab}) = \frac{\mathrm{Re}(\hat{e}^{0n_{\rm ex}}_a, \hat{e}^{0n_{\rm ex}}_b)}{||\hat{e}^{0n_{\rm ex}}_a||\,||\hat{e}^{0n_{\rm ex}}_b||} = \mathrm{Re}(Q^{0n_{\rm ex}}_{ab})/\sqrt{Q^{0n_{\rm ex}}_{aa} Q^{0n_{\rm ex}}_{bb}}$. Using Eq. (S56), it then naturally follows that $\nabla_{\boldsymbol{\kappa}}||\hat{e}^{0n_{\rm ex}}_a|| = 0$ and $\nabla_{\boldsymbol{\kappa}}\cos(\theta^{0n_{\rm ex}}_{ab}) = 0$.

For delocalized transitions, notice that $Q^{0n_{\rm d}}_{ba}$ is precisely the Wilson loop $\mathcal{W}^{0n_{\rm d}}_{ba}$, which depends sensitively on $\boldsymbol{\kappa}$ (Fig. (2)). Therefore in general $\nabla_{\boldsymbol{\kappa}} Q^{0n_{\rm d}}_{ba} \neq 0$, and hence parallel transporting tangent vectors is not necessarily norm- or angle-preserving.

The Hermitian connection follows directly from the parallel transport rule. For general many-body transitions: $C^{0n}_{bca} = (\hat{e}^{0n}_b, \nabla_{\kappa_c}(\hat{e}^{0n}_a)) = r^b_{n0} D_{\kappa_c}(r^a_{0n})$, where $D_{\kappa_c}(r^{0n}_a) = \partial_{\kappa_c}(r^a_{0n}) + r^a_{0n}(i\mathcal{A}^c_n - i\mathcal{A}^c_0)$. And for exciton transition: $C^{0n_{\rm ex}}_{bca} = (\hat{e}^{0n_{\rm ex}}_b, \nabla_{\kappa_c}(\hat{e}^{0n_{\rm ex}}_a)) = i\mathcal{R}^c_{0n_{\rm ex}} r^b_{n_{\rm ex}0} r^a_{0n_{\rm ex}}$. In particular, we have $C^{0n_{\rm ex}}_{bca} + (C^{0n_{\rm ex}}_{acb})^* = 0$ for excitons according to

Eq. (S56), which in general does not hold for delocalized particle-hole pairs.

---

[1] Kohn, W. Theory of the insulating state. *Physical review* **133**, A171 (1964).
[2] Souza, I., Wilkens, T. & Martin, R. M. Polarization and localization in insulators: Generating function approach. *Physical Review B* **62**, 1666 (2000).
[3] Luttinger, J. The effect of a magnetic field on electrons in a periodic potential. *Physical Review* **84**, 814 (1951).
[4] Shi, L.-k., Zhang, D., Chang, K. & Song, J. C. Geometric photon-drag effect and nonlinear shift current in centrosymmetric crystals. *Physical Review Letters* **126**, 197402 (2021).
[5] Resta, R. Geometrical theory of the shift current in presence of disorder and interaction. *Physical Review Letters* **133**, 206903 (2024).
[6] Sgiarovello, C., Peressi, M. & Resta, R. Electron localization in the insulating state: Application to crystalline semiconductors. *Physical Review B* **64**, 115202 (2001).
[7] Taherinejad, M., Garrity, K. F. & Vanderbilt, D. Wannier center sheets in topological insulators. *Physical Review B* **89**, 115102 (2014).
[8] Gresch, D. *et al.* Z2pack: Numerical implementation of hybrid wannier centers for identifying topological materials. *Physical Review B* **95**, 075146 (2017).
[9] Keldysh, L. V. *Coulomb interaction in thin semiconductor and semimetal films*, 155–158.
[10] Cudazzo, P., Tokatly, I. V. & Rubio, A. Dielectric screening in two-dimensional insulators: Implications for excitonic and impurity states in graphane. *Physical Review B—Condensed Matter and Materials Physics* **84**, 085406 (2011).
[11] Fu, L. & Kane, C. L. Time reversal polarization and a z 2 adiabatic spin pump. *Physical Review B—Condensed Matter and Materials Physics* **74**, 195312 (2006).
[12] Liu, G., Shan, W., Yao, Y., Yao, W. & Xiao, D. Three-band tight-binding model for monolayers of group-vib. *Phys Rev B* **88**, 085433 (2013).
[13] Dong, Y. *et al.* Giant bulk piezophotovoltaic effect in 3r-mos2. *Nature nanotechnology* **18**, 36–41 (2023).
[14] Rostami, H., Roldán, R., Cappelluti, E., Asgari, R. & Guinea, F. Theory of strain in single-layer transition metal dichalcogenides. *Physical Review B* **92**, 195402 (2015).
[15] Wu, F., Qu, F. & MacDonald, A. H. Exciton band structure of monolayer mos 2. *Physical Review B* **91**, 075310 (2015).
[16] Marzari, N. & Vanderbilt, D. Maximally localized generalized wannier functions for composite energy bands. *Physical review B* **56**, 12847 (1997).
