# Supplementary material for: Correlated quantum shift vector of particle-hole excitations
Source: Nat Commun. 2026 May 15;17:6465. doi: 10.1038/s41467-026-72878-8 (PMC13376538; doi:10.1038/s41467-026-72878-8)
Supplement: Supplementary file 2 — Transparent Peer Review file [file 41467_2026_72878_MOESM2_ESM.pdf]

nature portfolio

Peer Review File

Correlated quantum shift vector of particle-hole excitations

Corresponding Author: Professor Justin Song

This file contains all reviewer reports in order by version, followed by all author rebuttals in order by version.

Version 0:

Reviewer comments:

Reviewer #1

(Remarks to the Author)
This paper shows that there is a fundamental difference in the optical response between exciton bound states and free electron-hole pairs. The authors trace this difference back to how exciton and free electron-hole wavefunctions transform under flux insertions, a result that is interesting in its own right from a theoretical / conceptual point of view. At the same time, the paper shows how optical responses can be used to characterize the excitons and crystal symmetry of a given material, it is therefore also of immediate practical application. This point is underscored by the section on (non-)polar point groups and the concrete predictions for 3R-MoS$_2$.

We find that this paper is generally well written, technically sound, and a useful contribution to the rapidly growing field studying the excitons and quantum geometry. We are therefore happy to recommend it for publication subject to responses to the following comments:

1. Eq. (2) is mathematically problematic. Does the derivative act on $\delta_{\boldsymbol{r}, \boldsymbol{r}'}$? Also, is it clear that $\mathcal{V}(\boldsymbol{r}, \boldsymbol{r}')$ does not depend on total momentum $\boldsymbol{Q}$? (\emph{e.g.} Eq. S5 and S6 seem to be momentum dependent.)

2. What exactly is meant by flux insertion? This notion needs to be fully defined mathematically before it can be used in equations such as Eq.(3).

3. The authors discuss the deviation of the envelope function at the ``system boundary" but it is not immediately clear what is meant by this, since it seems incompatible with the use of periodic/twisted boundary conditions. Moreover, since the authors consider the relative rather than absolute position coordinate, it is unclear to what extent this coordinate can have a ``boundary" that is independent of the center of mass. In general, we think this section could be clarified significantly to aid the reader not already familiar with flux insertion. (In addition, it would be good to explicitly state the definition of flux insertion/ twisted boundary conditions, as already remarked above.)

4. The statement ``Flux-inserted Wannier functions characterize the quantum geometry of electronic systems" is too vague and more confusing than useful. Similarly, in the statement: ``Our work demonstrates that strong electron-hole interaction radically transforms excited state quantum geometry", what exactly do the authors mean by ``quantum geometry" in the present context?

5. We note that the expression for the exiton transition shift vector [Eq.(8)] can be further simplified using,
\begin{equation}
\int \mathrm{d}\boldsymbol{x} \: w^*_{c(v), 0}(\boldsymbol{x}) \boldsymbol{x}w_{c(v), \boldsymbol{R}}(\boldsymbol{x}) = \delta_{\boldsymbol{R}, 0} \frac{\gamma_{c(v)}}{2\pi},
\end{equation}
where $\frac{\gamma_{c(v)}}{2\pi}$ is the (maximally localized) electronic Wannier center and $\gamma_{c(v)}$ is the Berry phase. This means that the total expression can be written in the more compact form
\begin{equation}
\boldsymbol{\mathcal{R}}_{0\rightarrow\mathrm{ex}} \, \dot{=} \, \frac{\gamma_{c} - \gamma_{v}}{2\pi} + \sum_{r}|\psi_Q(r)|^2 r.
\end{equation}
In this particular form, it is a bit clearer that this represents the exciton polarization. As multiple definitions of the exciton polarization exist in the literature, it would be helpful if the authors discuss briefly to what extent their definition is compatible

with these (see for example the momentum space expression given for the exciton polarization in arXiv:2507.22983, Eq. 20, or also arXiv:2408.10300, Eq. 24).

6. It would be very helpful if the authors take a bit more space to explain in what sense the finding that ``the quantum shift vector for excitonic optical excitations is independent of the light polarization" is counter-intuitive.

7. Lastly, we spotted a few minor typos: the definition of $d_{c(v)}(\boldsymbol{R})$ in the main text mistakenly uses the position operator ($\boldsymbol{\hat{\mathrm{x}}}$) not the vector position $\boldsymbol{x}$. The definition of $d_{c}(\boldsymbol{R})$ in appendix V [under Eq. (S31)] also mistakenly uses the position operator and also misses the $\mathrm{d}\mathrm{x}$.

Reviewer #2

(Remarks to the Author)
The authors address for the first time the shift vector of a quantum-system discriminating between the cases of localized and de-localised electron hole excitons. These are described through the Bethe-Salpeter formalism. Although the authors leverage on a modern theory of the shift current (Ref [6] in the manuscript), their advances, namely the analysis in terms of the localisation properties of the excited states, are significant. Indeed, they could explain, with this, recent experimental findings.
The manuscript is clearly written. I think it lies within the impact zone of nature communication. I suggest publication after the authors will have considered the following minor point:

The paragraph regarding the possible first-principles calculation with the GW-BSE method should be expanded. If possible a trial calculation should be inserted as well.

I noticed also a typo on the first page-first column: ‘posssess’

Reviewer #3

(Remarks to the Author)
I co-reviewed this manuscript with one of the reviewers who provided the listed reports. This is part of the Nature Communications initiative to facilitate training in peer review and to provide appropriate recognition for Early Career Researchers who co-review manuscripts.

Reviewer #4

(Remarks to the Author)
In this work, Yang Srivastava, and Song have adapted the flux insertion technique to the Bethe salpeter Hamiltonian for excitons. They show the flux couples to the relative e-h coordinate so that the flux can be gauged away for bound excitons up to exponentially small boundary corrections. Then, they define an exciton shift vector as a derivative with respect to the flux and argue it is insensitive to the polarization of the external field and transforms as a polar vector. As a consequence, it must be zero for non-polar point groups.

While it is interesting to see the parallel in the mathematical structure between the shift current and earlier ideas about quantum geometry, conceptually, there does not seem to be much that is fundamentally new in this paper. The flux insertion is a standard technique and the derived dependence of the shift vector on polar symmetry is true for any vector and also seen in the symmetry of the shift current conductivity tensor derived in previous work. There does not seem to be a clear measurable prediction that differs from previous work.

If the authors wish to strengthen the points they are making, they could consider expounding on the following points, which could add to the theoretical novelty of the paper

1. Flux insertion is typically justified by the idea that you have coupling to a physical gauge field, allowing the integrated phase response to be expressed as a curvature or geometric quantity, such as a Berry curvature or quantum metric. In this case, the flux acts on the relative coordinate of the exciton and the response is a matrix element phase derivative not clearly tied to a curvature. The mathematical analogy seems convincing, but it would be more insightful to explicitly show the relation of the shift vector to an intrinsic differential geometric object (in the same way the Berry curvature is an intrinsically defined curvature tensor). Morimoto and Nagaosa have derived such a mapping for the single particle shift vector but not the exciton
2. The flux insertion technique is also related to the Laughlin argument in quantum Hall effects. Is there any connection between the unbound electron-hole pairs’ spectrum’s susceptibility to the inserted flux and, say, how the wave functions of electrons are distorted in the presence of a magnetic field as in integer quantum Hall effect?
3. It is not really clear how Eq. 7 follows directly from the decay property in Eq. 4. The argument appears to assume that the off-diagonal elements of the BSE kernel decay exponentially and that the exciton envelope is sufficiently localized, but this connection is not rigorously demonstrated in the main text. It would help if the authors explicitly showed how the localization of the exciton wavefunction quantitatively ensures the flux-independence of the overlap.
4. The numerical calculations also appear somewhat insufficient to support all the claims in the paper. It would be valuable to show the shift vector with and with-out electron-hole interactions to explicitly demonstrate the role of excitons relative to

the independent particle picture. Also, it would be helpful if the shift current conductivity tensor could be calculated in addition to just the shift vector for a clearer connection to physical observables.
5. It would be greatly helpful if the authors can provide a numerical example of how the shift vector does not transform as a vector when the exciton is not strongly bound.

Overall, it seems like the main contribution of the paper is in introducing a more sophisticated theoretical interpretation of known phenomena. Additional analysis of the physical meaning and experimental observables would strengthen the paper, but even then, this paper may be more appropriate for a more technical or theoretically-focused journal such as Physical Review Letters or Physical Review B.

Reviewer #5

(Remarks to the Author)
I co-reviewed this manuscript with one of the reviewers who provided the listed reports. This is part of the Nature Communications initiative to facilitate training in peer review and to provide appropriate recognition for Early Career Researchers who co-review manuscripts.

Version 1:

Reviewer comments:

Reviewer #1

(Remarks to the Author)
The authors have responded adequately to our concerns and we are happy to recommend the manuscript for publication.

Reviewer #3

(Remarks to the Author)
I co-reviewed this manuscript with one of the reviewers who provided the listed reports. This is part of the Nature Communications initiative to facilitate training in peer review and to provide appropriate recognition for Early Career Researchers who co-review manuscripts.

Reviewer #4

(Remarks to the Author)
The authors have responded thoroughly to my questions, and the revisions substantially improved the paper. In particular, the explicit demonstration that standard tensor symmetry analysis of existing exciton shift current formulas permits nonzero responses, while the current analysis shows the exciton shift current must vanish, clarifies that the result goes beyond what was previously known. I think my initial review underestimated the subtlety of the distinction between symmetry constraints on the shift current tensor versus the shift vector itself. The new discussion on many-body quantum geometry and numerical calculations comparing excitonic and free particle-hole responses also add significant value. I recommend acceptance.

Reviewer #5

(Remarks to the Author)
I co-reviewed this manuscript with one of the reviewers who provided the listed reports. This is part of the Nature Communications initiative to facilitate training in peer review and to provide appropriate recognition for Early Career Researchers who co-review manuscripts.

Open Access This Peer Review File is licensed under a Creative Commons Attribution 4.0 International License, which permits use, sharing, adaptation, distribution and reproduction in any medium or format, as long as you give appropriate credit to the original author(s) and the source, provide a link to the Creative Commons license, and indicate if changes were made.
In cases where reviewers are anonymous, credit should be given to 'Anonymous Referee' and the source.
The images or other third party material in this Peer Review File are included in the article's Creative Commons license, unless indicated otherwise in a credit line to the material. If material is not included in the article's Creative Commons license and your intended use is not permitted by statutory regulation or exceeds the permitted use, you will need to obtain permission directly from the copyright holder.
To view a copy of this license, visit https://creativecommons.org/licenses/by/4.0/

Detailed point-by-point response to Reviewer 1

Reviewer 1: We find that this paper is generally well written, technically sound, and a useful contribution to the rapidly growing field studying the excitons and quantum geometry. We are therefore happy to recommend it for publication subject to responses to the following comments:

Our response: We thank the Reviewer positive assessment of our work. We answer all the Reviewer's comments and questions below fully in detail below and have implemented all suggestions in our revised text. This has helped improve the clarity of our work.

1. **Reviewer 1:** Eq. (2) is mathematically problematic. Does the derivative act on $\delta_{\boldsymbol{r},\boldsymbol{r}'}$? Also, is it clear that $\mathcal{V}(\boldsymbol{r},\boldsymbol{r}')$ does not depend on total momentum $\boldsymbol{Q}$? (*e.g.* Eq. S5 and S6 seem to be momentum dependent.)

   Our response: We thank the Reviewer for spotting these typographical issues. Indeed, the derivative $\partial_{\mathbf{r}}$ does not act on the Kronecker delta $\delta_{\boldsymbol{r},\boldsymbol{r}'}$. It only acts on the wavefunctions as per the standard treatment in Wannier's paper on excitons, see Physical Review 52, 191 (1937). For clarity, we have moved the Kronecker delta function to the front to avoid confusion. Additionally, the reviewer is also right that $\mathcal{V}(\boldsymbol{r},\boldsymbol{r}')$ does indeed depend on the center of mass momentum $\boldsymbol{Q}$ (as referee noticed is shown in Eq. S5 and S6); we had suppressed showing the explicit $\boldsymbol{Q}$ dependence in the main text for brevity but have now restored the explicit $\boldsymbol{Q}$ dependence for full clarity.

2. **Reviewer 1:** What exactly is meant by flux insertion? This notion needs to be fully defined mathematically before it can be used in equations such as Eq.(3).

   Our response: We thank the Reviewer for the suggestion on improving our presentation. We define flux insertion as follows.

   1. Flux insertion is implemented by imposing periodic boundary conditions along one direction to form a cylinder and threading a static gauge flux ϕ through the hole. This induces a uniform static vector potential $\boldsymbol{\kappa}$ along the periodic direction, with $\oint \boldsymbol{\kappa}\cdot d\mathbf{l} = \phi$ (see Fig. R1).

   2. When a particle with charge q move along a path L in the background of $\boldsymbol{\kappa}$, it accumulates a quantum mechanical phase $e^{-iq\int_L \boldsymbol{\kappa}\cdot d\boldsymbol{l}}$. Consequently, the flux-inserted electronic Wannier function is modified by a phase dependent on the electron's displacement from its Wannier center: $w^{\boldsymbol{\kappa}}_{\boldsymbol{R}}(\mathbf{x}) = e^{-i\boldsymbol{\kappa}\cdot(\mathbf{x}-\boldsymbol{R})}w_{\boldsymbol{R}}(\mathbf{x})$.

   3. Crucially, since $\boldsymbol{\kappa}$ couples to charge of the particle, the accumulated phase has opposite signs for electrons and holes. As a result, the co-motion of an electron and a hole incurs no net phase, and $\boldsymbol{\kappa}$ couples only to the relative coordinate $\boldsymbol{r}$ (see Fig. R1).

   4. Accordingly, the flux-inserted BSE Hamiltonian picks up a phase factor corresponding to the change in this relative coordinate. As the BSE Hamiltonian annihilates a particle-hole pair with separation $\mathbf{r}'$ and creates a particle-hole pair with separation $\mathbf{r}$, the matrix element becomes: $\mathcal{H}^{\boldsymbol{\kappa}}_{\boldsymbol{Q}}(\mathbf{r},\mathbf{r}') = e^{-i\boldsymbol{\kappa}\cdot(\mathbf{r}-\mathbf{r}')}\mathcal{H}_{\boldsymbol{Q}}(\mathbf{r},\mathbf{r}')$.

   Change: we have add the above discussion in the main text right after we introduced flux threading as well as added the schematic figure illustrating Flux threading to the supplement as Fig S1 with reference to it from both the main text and supplement.

3. **Reviewer 1:** The authors discuss the deviation of the envelope function at the "system boundary" but it is not immediately clear what is meant by this, since it seems incompatible with the use of periodic/twisted boundary conditions. Moreover, since the authors

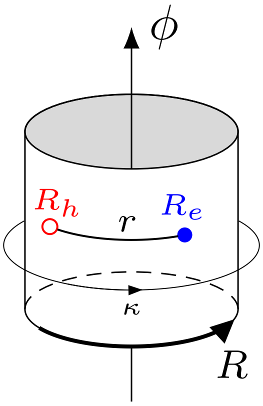

Figure R1: *A schematic of flux threading.* The system is wrapped to a cylinder, with flux ϕ piercing through the hole. $\boldsymbol{R}$ indicates the coordinates of particles. Electrons and holes carry opposite charges, resulting in opposite signs of the phase factor when moving in a background of vector potential $\boldsymbol{\kappa}$. Consequently, only the relative coordinate $\boldsymbol{r} = \boldsymbol{R}_e - \boldsymbol{R}_h$ couples to $\boldsymbol{\kappa}$.

consider the relative rather than absolute position coordinate, it is unclear to what extent this coordinate can have a "boundary" that is independent of the center of mass. In general, we think this section could be clarified significantly to aid the reader not already familiar with flux insertion. (In addition, it would be good to explicitly state the definition of flux insertion/ twisted boundary conditions, as already remarked above.)

Our response: We thank the Reviewer for the suggestion on improving our presentation. We acknowledge this is a confusing point and what we meant by "boundary" needs further elaboration especially for the relative coordinates of the particle-hole excitations that we consider. Here we have adopted the use of the word "boundary" in order to make contact with the existing literature on flux threading, e.g., Physical Review 133.A171 (1964), Physical Review B 31, 3372 (1985), and Physical Review B 62, 1666 (2000).

To understand this nomenclature it is instructive to examine the procedure for flux insertion. To perform flux insertion along a given direction $\boldsymbol{e}_\kappa$, we wrap the system into a periodic ring along this direction. All coordinates then satisfy periodic boundary conditions; for example, the Wannier functions obey $w_{\boldsymbol{R}}(\mathbf{x}+L\boldsymbol{e}_\kappa) = w_{\boldsymbol{R}}(\mathbf{x})$. From the electron and hole coordinates, $\mathbf{R}_e$ and $\mathbf{R}_h$, we define the relative coordinate $\mathbf{r} = \mathbf{r}_e - \mathbf{r}_h$ and center-of-mass coordinate $\mathbf{R}_{\text{cm}} = (\mathbf{R}_e + \mathbf{R}_h)/2$. Functions expressed in these coordinates inherit the system's periodicity. Specifically, winding the electron around the periodic ring shifts the relative coordinates $\mathbf{r} \to \mathbf{r} + L\boldsymbol{e}_\kappa$, so the envelope function shares the periodicity of the system: $\psi_{\boldsymbol{Q}}(\mathbf{r} + L\boldsymbol{e}_\kappa) = \psi_{\boldsymbol{Q}}(\mathbf{r})$.

Critically, although the envelope function is periodic, it is strongly localized for excitons near $|\mathbf{r} \cdot \boldsymbol{e}_\kappa| = 0 \bmod L$, i.e., when **electrons and holes are close**. This allows a clear distinction between bulk and boundaries. We define the "boundary" region as the vicinity of $|\mathbf{r} \cdot \boldsymbol{e}_\kappa| = L/2 \pmod{L}$, corresponding to the **maximal electron-hole separation**. It is precisely in this "boundary" region (maximal electron-hole separation) that the distinction between delocalized and localized particle-hole pairs becomes apparent: the delocalized particle-hole envelope functions remain non-negligible at the "boundary", while exciton envelope functions are exponentially suppressed there in the large system size limit.

Changes: We have added a clear description of flux threading right after we introduced

the idea. Additionally, we have now defined what we meant by "boundary", which is defined in the relative coordinate space where the electrons and holes are maximally separated.

4. **Reviewer 1:** The statement "Flux-inserted Wannier functions characterize the quantum geometry of electronic systems" is too vague and more confusing than useful. Similarly, in the statement: "Our work demonstrates that strong electron-hole interaction radically transforms excited state quantum geometry", what exactly do the authors mean by "quantum geometry" in the present context?

   Our response: We thank the Reviewer for encouraging us to provide a deeper examination of these points. We acknowledge that our initial explanation was too brief to be fully clear. We appreciate the opportunity to elaborate further on these interesting aspects. We now answer in two parts:

   (A): In response to Reviewer's first comment on the sentence "Flux-inserted Wannier functions characterize the quantum geometry of electronic systems", we answer below. What we meant by the quantum geometry associated with non-interacting Wannier functions was that Berry connection, Berry curvature and related quantum geometric quantities can be readily derived from the flux-threaded Wannier functions by taking a derivative with respect to flux.

   For example, with the flux inserted Wannier function $w^{\boldsymbol{\kappa}}_{\mathbf{R}}(\mathbf{x}) = e^{-i\boldsymbol{\kappa}\cdot(\mathbf{x}-\mathbf{R})}w_{\mathbf{R}}(\mathbf{x})$, the matrix elements of the position operator are obtained from derivatives with respect to $\boldsymbol{\kappa}$:

$$i \lim_{\boldsymbol{\kappa}\to 0} \langle w^{\boldsymbol{\kappa}}_{\mathbf{R}}|\partial_{\boldsymbol{\kappa}}|w^{\boldsymbol{\kappa}}_{\mathbf{0}}\rangle = \int d\mathbf{x} w^{*}_{\mathbf{R}}(\mathbf{x})\mathbf{x}w_{\mathbf{0}}(\mathbf{x}). \tag{R1}$$

   The Berry connection then follows directly as the Fourier transform of these matrix elements,

$$i \langle u_{\mathbf{k}}| \nabla_{\mathbf{k}} |u_{\mathbf{k}}\rangle = i \sum_{\mathbf{R}} e^{-i\mathbf{k}\cdot\mathbf{R}} \lim_{\boldsymbol{\kappa}\to 0} \langle w^{\boldsymbol{\kappa}}_{\mathbf{R}}|\partial_{\boldsymbol{\kappa}}|w^{\boldsymbol{\kappa}}_{\mathbf{0}}\rangle\,, \tag{R2}$$

   cf. Physical Review B 56, 12847 (1997). In similar spirit, people have used flux threading to study many-body Berry curvature (Physical Review B 31, 3372 (1985)) and many-body quantum metric (Physical Review B 62, 1666 (2000)) in generic many-body interacting systems.

   We admit that space is limited for explaining these interesting ideas, and to maintain focus on the correlated quantum geometry, we have removed the statement regarding the quantum geometry associated with single-particle Wannier functions. We have replaced it with the following sentence with message more concretely conveyed "Flux-insertion has been used in the literature to describe the many-body Berry curvature and quantum metric of electronic systems" and inserted the appropriate references to literature, namely Physical Review B 31, 3372 (1985), and Physical Review B 62, 1666 (2000).

   (B): In response to Reviewer's second comment on "strong electron-hole interaction radically transforms excited state quantum geometry", we now clarify what we mean by "quantum geometry" and how interaction radically transforms it. The geometry of resonant optical transitions in **non-interacting bands** has recently undergone intense scrutiny (see a most up-to-date account by Ahn, Guo, Nagaosa, Vishwanath, Nature Physics 18, 290-295 (2022)), where the central object idea is the Hermitian connection governing parallel transport of the tangent vector of optical transitions.

   Using flux threading, we have extended the Riemannian geometry framework to many-body resonant transitions. The many-body tangent vector for a resonant optical transition is defined as

$$\hat{e}^{0n}_{a}(\boldsymbol{\kappa}) \equiv |\Phi_0(\boldsymbol{\kappa})\rangle\, r^{a}_{0n}(\boldsymbol{\kappa})\, \langle\Phi_n(\boldsymbol{\kappa})|\,. \tag{R3}$$

Parallel transport of this tangent vector is captured by the covariant derivative $\nabla_{\boldsymbol{\kappa}}$, defined by projection of its derivative onto the tangent plane:

$$\nabla_{\boldsymbol{\kappa}} \hat{e}_a^{0n}(\boldsymbol{\kappa}) = P_0 \big[\partial_{\boldsymbol{\kappa}} \hat{e}_a^{0n}(\boldsymbol{\kappa})\big] P_n, \tag{R4}$$

where $\partial_{\boldsymbol{\kappa}}$ is the ordinary derivative and $P_{0/n} = |\Phi_{0/n}\rangle \langle \Phi_{0/n}|$ are projectors.

By construction, $\nabla_{\boldsymbol{\kappa}} \hat{e}_a^{0n}$ lies in the tangent plane and can thus be expanded in the tangent basis via the Hermitian connection C_{bac}^{0n} associated with parallel transport:

$$\nabla_c \hat{e}_a^{0n} = \sum_b (C^{0n})^b_{ca} \hat{e}_b^{0n}, \quad C_{bca}^{0n} \equiv \sum_e Q_{be}^{0n} (C^{0n})^e_{ca} = (\hat{e}_b^{0n}, \nabla_{\kappa_c} \hat{e}_a^{0n}), \tag{R5}$$

where the many-body Hermitian metric $Q_{ba}^{0n} \equiv (\hat{e}_b^{0n}, \hat{e}_a^{0n}) = r_{n0}^b r_{0n}^a$ determines the optical transition rate.

Resonant optical responses can thus be succinctly represented using this geometric language. For example, the many-body shift current is determined by the Hermitian connection tensor:

$$j^a_{\text{shift}} = -\frac{2\pi e^3}{\hbar} \sum_n \delta(E_{n0} - \hbar\omega)\, \text{Im}[C_{bab}^{n0}]\, |E^b|^2. \tag{R6}$$

Crucially, we find that, electron-hole interactions can produce fundamentally different Riemannian-geometric structure for exciton bound states as compared to the delocalized free particle–hole pairs. Using Eq. (R4) and (R5), we explicitly evaluate the Riemannian geometry structure for these two cases: (i) delocalized particle-hole pairs, (ii) strongly-bound exciton pairs.

From our shift vector discussion, we find that the covariant derivative (by directly evaluating Eq. (R4)) has the structure:

$$\nabla_c(\hat{e}_a^{0n}) = \overbrace{[D_{\kappa_c} \ln(r_{0n}^a)] \hat{e}_a^{0n}}^{\text{General form for delocalized p-h pairs}} \quad ; \quad \nabla_c(\hat{e}_a^{0n}) = \overbrace{i\mathcal{R}_{0n}^c \hat{e}_a^{0n}}^{\text{Factorized form for exciton bound pairs}} \quad . \tag{R7}$$

where parallel transport of the tangent vector of delocalized e-h pairs is multiplied by a complex coefficient $D_{\kappa_c} \ln(r_{0n}^a)$ (with D the covariant derivative: $D_{\kappa_c} \ln(r_{0n}^a) = \partial_{\kappa_c} \ln(r_{0n}^a) + i(\mathcal{A}_0^c - \mathcal{A}_n^c)$) depending on the direction of the tangent vector (see Nature Physics 18, 290-295 (2022)).

In sharp contrast, we find the tangent vector of the exciton bound pairs is only multiplied by a purely imaginary number (i times the exciton shift vector $\mathcal{R}_{0n}^c$) independent of the tangent vector. **Geometrically, this means that parallel transport of the exciton tangent vector is norm and angle-preserving**. Parallel transport of the delocalized pair tangent vector does **not** have these geometric properties.

This has important implications for the Hermitian connection which is determined by the parallel transport of tangent vector:

$$C_{bca}^{0n} = \overbrace{r_{n0}^b D_{\kappa_c}(r_{0n}^a)}^{\text{General form for delocalized p-h pairs}} \quad ; \quad C_{bca}^{0n} = \overbrace{i\mathcal{R}_{0n}^c r_{n0}^b r_{0n}^a}^{\text{Factorized form for exciton bound pairs}} \quad . \tag{R8}$$

Their distinct forms indicate fundamentally different responses for the two cases. For example, since the exciton shift vector $\mathcal{R}_{0n}$ is independent of light polarization, the polarization dependence of the exciton Hermitian connection arises solely from the optical transition matrix elements. For linearly polarized light along direction b, the linear shift current—determined by the imaginary part of the Hermitian connection—factorizes as

$\mathcal{R}^{c}_{0n}|r^{b}_{0n}|^2$. Since $|r^{b}_{0n}|^2$ is positive-definite, the direction of linear shift current remains unchanged when the light polarization is varied. In contrast, the shift vector of delocalized pairs, defined in literature by $\mathcal{R}^{c,a}_{0n} = \mathrm{Im}(C^{0n}_{aca})/Q^{0n}_{aa}$, depends on the light-polarization direction a, and consequently its corresponding shift current can change sign with changing light-polarization direction (cf. Physical Review B 61.5337 (2000), Nature Physics 18, 290-295 (2022)).

Change: We have incorporated the above discussion on many-body quantum geometry into the main text as a new section entitled *Excitonic vs delocalized particle-hole excitation quantum geometry.*

5. **Reviewer 1:** We note that the expression for the exiton transition shift vector [Eq.(8)] can be further simplified using,

$$\int \mathrm{d}\boldsymbol{x}\, w^{*}_{c(v),0}(\boldsymbol{x})\boldsymbol{x}w_{c(v),\boldsymbol{R}}(\boldsymbol{x}) = \delta_{\boldsymbol{R},0}\frac{\gamma_{c(v)}}{2\pi}, \tag{R9}$$

where $\frac{\gamma_{c(v)}}{2\pi}$ is the (maximally localized) electronic Wannier center and $\gamma_{c(v)}$ is the Berry phase. This means that the total expression can be written in the more compact form

$$\boldsymbol{\mathcal{R}}_{0\to\mathrm{ex}} \doteq \frac{\gamma_c - \gamma_v}{2\pi} + \sum_r |\psi_Q(r)|^2 r. \tag{R10}$$

In this particular form, it is a bit clearer that this represents the exciton polarization. As multiple definitions of the exciton polarization exist in the literature, it would be helpful if the authors discuss briefly to what extent their definition is compatible with these (see for example the momentum space expression given for the exciton polarization in arXiv:2507.22983, Eq. 20, or also arXiv:2408.10300, Eq. 24).

Our response: We thank the Reviewer for bridging our works with various ideas in the literature. We answer in two parts:

(A): We believe that the simplification suggested by Reviewer does not generally hold. When the spatial coordinate $\boldsymbol{x}$ is not sandwiched in Eq. (R9) of the reply, it is true that the Wannier functions center at different sites are orthogonal, i.e., $\int \mathrm{d}\boldsymbol{x}\, w^{*}_{c(v),0}(\boldsymbol{x})w_{c(v),\boldsymbol{R}}(\boldsymbol{x}) = \delta_{\boldsymbol{R},0}$. Putting in $\boldsymbol{x}$, however, we are essentially dealing with the matrix elements of the position operator in the Wannier basis, see e.g., Eq. (4) of the seminal paper by Marzari & Vanderbilt (Physical Review B 56, 12847 (1997)):

$$\langle \mathbf{R}n|\boldsymbol{x}|\mathbf{0}m\rangle = i\frac{V}{(2\pi)^d}\int d\mathbf{k}e^{i\mathbf{k}\cdot\mathbf{R}}\langle u_{n,\mathbf{k}}|\nabla_{\mathbf{k}}|u_{m,\mathbf{k}}\rangle\,, \tag{R11}$$

where their $\mathbf{R}$ and $\mathbf{0}$ have the same meaning as us, which are used to label lattices sites where Wannier functions are centered at, and n, m are band indices. The Reviewer suggests that $\langle \mathbf{R}n|\boldsymbol{x}|\mathbf{0}n\rangle = 0$ for $\mathbf{R} \neq \mathbf{0}$. However, it is precisely $\langle \mathbf{R}n|\boldsymbol{x}|\mathbf{0}m\rangle$ elements that control the gauge-dependent part of the Wannier function spreading $\langle \boldsymbol{x}^2\rangle - \langle \boldsymbol{x}\rangle^2 = \sum_{\mathbf{R}\neq\mathbf{0}} |\langle \mathbf{R}n|\boldsymbol{x}|\mathbf{0}n\rangle|^2$ (the case of a single band n is shown in Eq. 15 in the Marzari Vanderbilt paper, Physical Review B 56, 12847 (1997)), and is in general non-zero even after minimizing it by a gauge choice to construct maximally localized Wannier functions (MLWFs).

On the other hand, it is instructive to deduce under what conditions the simplification that the reviewer suggests occurs. From Eq. 5 in the Marzari & Vanderbilt paper [Physical Review B 56, 12847 (1997)], the matrix elements of the position operator in the Wannier basis are connected to the Berry connection via Fourier transformation:

$$\langle u_{n,\mathbf{k}}|\nabla_{\mathbf{k}}|u_{m,\mathbf{k}}\rangle = -i\sum_{\mathbf{R}} e^{-i\mathbf{k}\cdot\mathbf{R}}\langle \mathbf{R}n|\boldsymbol{x}|\mathbf{0}m\rangle\,, \tag{R12}$$

From this, it follows that vanishing off-diagonal elements imply a uniform momentum space Berry connection consequently zero Berry curvature. Since we are interested in physical phenomena associated with non-trivial quantum geometry, we focus on the general case where $\langle \mathbf{R}n|\boldsymbol{x}|\mathbf{0}m\rangle$ elements are non-vanishing.

(B): We thank the Reviewer for pointing out these relevant literature. Yes, indeed the difference between electric polarization between initial and final states obtained from the real-space formulation of the shift vector coincides with the exciton's electric polarization. Our real-space formula [Eq. 10] for the non-degenerate case in our work is indeed compatible with the momentum space formulation of the exciton polarization found in arXiv:2507.22983 [Eq. 20] and also arXiv:2408.10300 [Eq. 24]. They are connected via a Fourier transform and application of Eq. 5 in Marzari & Vanderbilt paper. Even so, it is important to make two distinctions.

First, our work focusses on tracking the electric polarization change for excitonic transitions between excitonic states $\boldsymbol{\mathcal{R}}_{0\to\{\cdots\}\to\mathrm{ex}}$. Notice that $\boldsymbol{\mathcal{R}}_{0\to\{\cdots\}\to\mathrm{ex}}$ is *a priori* distinct from simply the electric dipole of the exciton by itself; instead, it deals with the accrual of polarization under excitonic transitions critical for the excitonic shift current. Our work shows that the details of the transitions are unimportant (they are exponentially suppressed in the thermodynamic limit) yielding $\boldsymbol{\mathcal{R}}_{0\to\{\cdots\}\to\mathrm{ex}} = \boldsymbol{\mathcal{R}}_{0\to\mathrm{ex}}$ that depends on the initial and final states only. In contrast, arXiv:2507.22983 [Eq. 20] and arXiv:2408.10300 [Eq. 24] expressions are motivated from gauge-invariance consideration alone aiming at finding the electric polarization of an excited excitonic state.

Second, when employing momentum space formulations for excitons, care needs to be taken when evaluating momentum-space integrals. For example, in arXiv:2408.10300, where a decaying envelope function at large relative momenta is assumed (Appendix C, below Eq.43). For tightly bound excitons, the envelope functions remain non-negligible throughout the Brillouin zone, so momentum-space integrals require careful treatment at the zone edges. Our Wannier-based real-space formula for shift vector, however, avoids this issue, as the envelope function decays exponentially with electron-hole separation, ensuring well-behaved physical quantities. This also highlights a computational advantage: our formula converges exponentially fast, requiring only a constant number of terms even for larger system, making it more efficient.

Finally, while these two works formulate the exciton shift vector in the non-degenerate case guided by gauge invariance, our formulation is in fact more powerful and can be used to track **degenerate** exciton states. To illustrate this we have now added a new section in **SI**, Sec. VIIB where we provide a general framework to also include degenerate excitons, which we describe below. In the degenerate case, a full description of the shift vector within the degenerate subspace at energy E_n requires a rank-three tensor $\mathcal{R}^a_{n_i,n_j}$, where n_i, n_j label states in the degenerate subspace. Owing to the degenerate subspace indices, the rank-three tensor $\mathcal{R}^a_{n_i,n_j}$ transforms under a basis rotation, and is therefore basis dependent. It acquires physical meaning only after contraction with the optical transition matrix elements $V_{0,n_i} \equiv \langle\Phi_0|\,\hat{v}\,|\Phi_{n_i}\rangle$, yielding a basis-independent and observable shift current. This entirely new formulation directly links the many-body exciton shift vector to realistic physical observables, revealing richer responses arising from exciton degeneracy.

6. **Reviewer 1:** It would be very helpful if the authors take a bit more space to explain in what sense the finding that "the quantum shift vector for excitonic optical excitations is independent of the light polarization" is counter-intuitive.

Our response: We thank the Reviewer for comment. We answer in two parts below:

(A): The shift vector for non-interacting particle-hole excitations is well known to depend

on the polarization of light through [e.g., Physical Review B 61, 5337 (2000)]

$$R_{cv}^{a,b}(\mathbf{k}) = \mathcal{A}_c(\mathbf{k}) - \mathcal{A}_v(\mathbf{k}) + \nabla_{\mathbf{k}}\mathrm{arg}(r_{cv}^b(\mathbf{k})) \tag{R13}$$

where a is the cartesian direction of the shift vector, r_{cv}^b is the optical transition matrix element with b the light polarization direction, and $\mathcal{A}_{c,v}(\mathbf{k})$ are the momentum-space Berry connections of the conduction and valence band electrons respectively. Critically, the light-polarization-dependent part $\nabla_{\mathbf{k}}\mathrm{arg}(r_{cv}^b(\mathbf{k}))$ is essential for ensure gauge invariance of $R_{cv}^{a,b}(\mathbf{k})$. Indeed, the non-interacting shift vector even for a specific vertical transition $v, \mathbf{k} \to c, \mathbf{k}$ is well known to depend on the polarization of light [see e.g., Figure 2I,L Science advances 5, eaav9743 (2019)].

(B): In contrast, our analysis demonstrates that the shift vector for the exciton transition is **independent** of the polarization of light. To demonstrate this visually for the reader we have now performed a numerical calculation of a MoS_2 three-band tight-binding model showing the shift vector for both free particle-hole pairs and the exciton shift vector (see Fig. R2). The free particle-hole pair shift vector indeed exhibits strong light-polarization dependence as shown in Fig. R2(c) and inset (Fig.3c and inset in the main text). In contrast, although the full formula for exciton shift vector contains the information of light polarization explicitly, the computed exciton shift vectors doesn't depend on light polarization, as shown in Fig. R2(d) and inset (Fig.3d and inset in the main text). This is a non-trivial consequence of its bound state nature as we have demonstrated.

Change: We have incorporated the above discussion into the second paragraph on page 5 and into the section *Excitonic transition shift vector in MoS_2* (and correspondingly Fig.3 in the main text), where we present a numerical simulation for MoS_2.

7. **Reviewer 1:** Lastly, we spotted a few minor typos: the definition of $d_{c(v)}(\boldsymbol{R})$ in the main text mistakenly uses the position operator ($\hat{\mathbf{x}}$) not the vector position $\boldsymbol{x}$. The definition of $d_c(\boldsymbol{R})$ in appendix V [under Eq. (S31)] also mistakenly uses the position operator and also misses the dx.

   Our response: We thank the Reviewer for the careful reading and pointing out these typos. We have removed the hats in the definition of $d_{c(v)}(\boldsymbol{R})$ and added back dx in Eq. (S31).

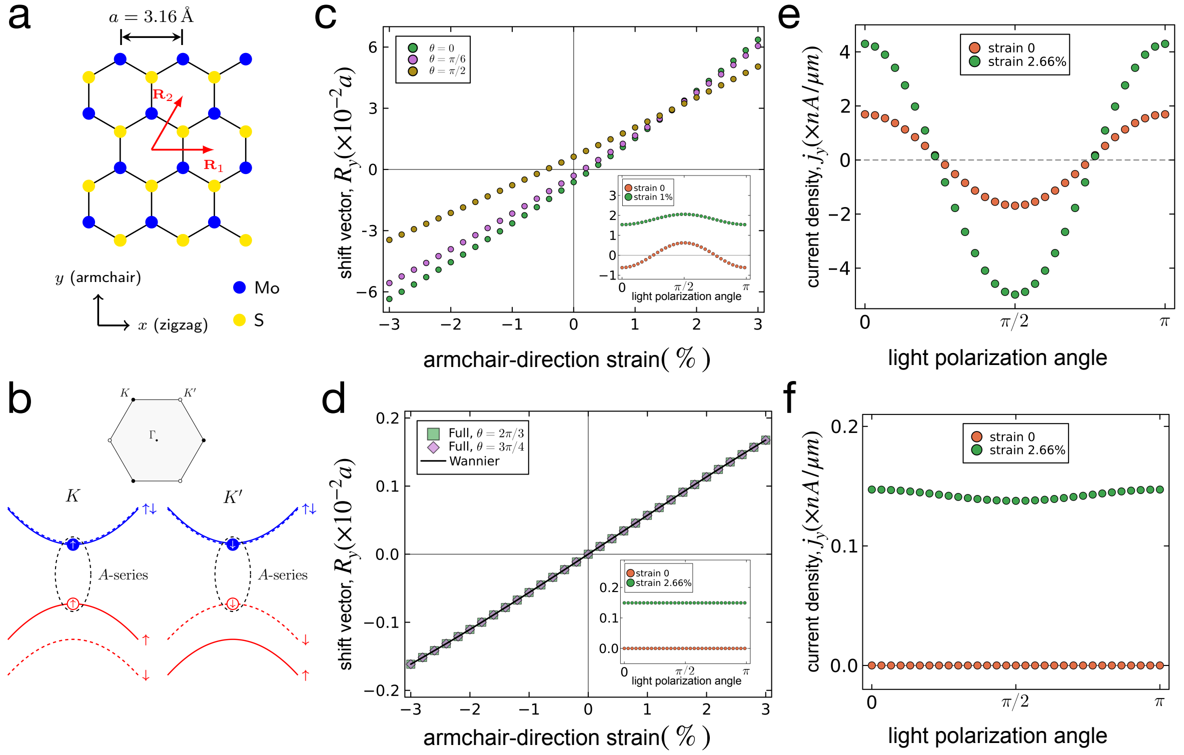

Figure R2: *Numerical calculation of shift vector and shift current in a three-band tight-binding model of MoS_2.* (a) Schematic of the monolayer MoS_2 lattice; arm chair direction is along y. (b) A-series excitons at the K, K' valleys in the Brillouin zone. Each exciton is two-fold valley-degenerate. Strong spin–orbit coupling splits the valence bands (with smaller conduction-band splittings) and produces spin-valley locking: K-valley A-excitons involve up-spin electron-hole pairs, while K'-valley excitons involve down-spin pairs. (c) Strain dependence of free particle-hole pair shift vectors for direct optical transitions at sampled momenta, which remain non-zero in the unstrained case and depend strongly on light-polarization angle θ (inset: polarization dependence of shift vector at K for the unstrained and 1% strained case). (d) Strain dependence of the A1s exciton shift vector at K composed of spin-up particle-hole pairs, which are polarization independent and vanish in the unstrained limit. Dots are computed via the full formula (Eq. (7) in the main text) and line is computed via the Wannier-based formula (Eq. (10) in the main text); both agree with each other. (inset: polarization dependence of A1s exciton shift vector for the unstrained and 2.66% strained ($\delta t/t = -0.1$) case via full formula). (e) Shift current for *free particle-hole pairs* at a photon energy of $\hbar\omega = 2.5$eV, for both zero strain and 2.66% strain ($\delta t/t = -0.1$). Strong polarization-dependent shift vectors cause the current to *reverse sign* as the polarization direction changes. (f) Shift current for A_{1s} *excitons* at photon energy $\hbar\omega = 1.08$eV with the same strains (e). Since the exciton shift vector is polarization-independent, the resulting excitonic transition shift current's weak polarization dependence comes from the squared optical matrix element and does not change sign.

Detailed point-by-point response to Reviewer 2

1. **Reviewer 2:** The manuscript is clearly written. I think it lies within the impact zone of nature communication. I suggest publication after the authors will have considered the following minor point: The paragraph regarding the possible first-principles calculation with the GW-BSE method should be expanded. If possible a trial calculation should be inserted as well.

   Our response: We thank the Reviewer for positive assessment as well as recommendation for onward publication. We answer the Reviewer's comment in two parts:

   (A) We thank the reviewer for the excellent suggestion to expand the paragraph describing how our formulae can be applied within a GW-BSE method. We have now expanded our discussion on the workflow of GW-BSE calculation (see "discussion" section of the revised main text), which we summarize below for the convenience of the Reviewer:

   The computational workflow is as follows. Bloch states are first obtained from *ab initio* DFT calculations and subsequently treated within either one-shot G_0W_0 or self-consistent GW, yielding undressed or quasiparticle-renormalized wave functions, respectively, with GW energies providing a more accurate description of optical gaps. Maximally localized Wannier functions (MLWFs) for the bands relevant for optical transitions are then constructed using Wannier90, providing a set of smooth single-particle basis. On this basis, the BSE kernel is constructed and solved to obtain excitonic envelope functions using standard packages such as YAMBO or BerkeleyGW. These envelope functions, combined with the MLWFs, allow direct evaluation of the excitonic shift vector via the Wannier-based formula Eq. (10). Multiplying the shift vectors by the corresponding optical transition rates then yields the exciton shift current.

   (B) The fully converged and relaxed DFT+GW-BSE calculation incorporating strain in a excitonic material is not the focus of our manuscript. Instead, we performed a realistic calculation using a "DFT-tight-binding" model of MoS_2, adopting parameters from the literature that were fitted to DFT band structure. In this approach, which demonstrates the Wannier workflow, we obtained Bloch functions by diagonalizing the Hamiltonian on a finite momentum grid and fixed the gauge via the Marzari-Vanderbilt routine to generate maximally-localized Wannier functions (MLWFs), see details in **SI** Sec. X. These gauge-fixed Bloch functions were then used to construct the BSE kernel and solve for the exciton envelope functions.

   To evaluate the exciton shift vector using the exciton envelope functions, we employed two methods:

   <u>**Method 1**</u> where we used the full expression given in Eq. (7) of the main text which involves a $\boldsymbol{\kappa}$ derivative and explicitly encodes the light-polarization. The BSE kernel at flux $\boldsymbol{\kappa}$ was constructed via interpolation of the MLWFs with zero flux, a technique called Wannier interpolation widely used in literature (see Physical Review B 96, 115147 (2017) and Physical Review B 97, 245143 (2018)). And the $\boldsymbol{\kappa}$ derivative was approximated using a finite difference scheme with envelope functions evaluated at small but finite $\boldsymbol{\kappa}$. Importantly, by explicitly find that the full formula Eq. (7) which explicit encodes light polarization, yields shift vectors that are independent of light polarization. This agrees with our analysis.

   We also performed a numerical calculation using the Wannier-based <u>**Method 2**</u> where we used the compact Wannier-based formula Eq. (10) of the main text. We find both Method 1 and Method 2 results coincide with each other; both exhibit light polarization independence (see Figure. R3). This further numerically validates our claim of exciton shift

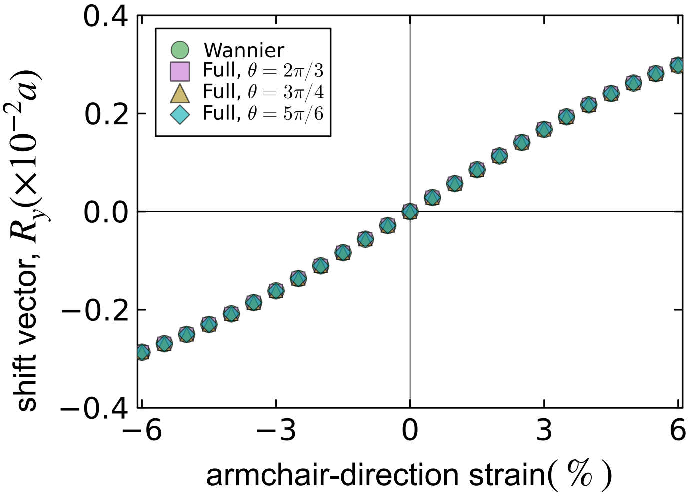

Figure R3: *Light-polarization independence of the exciton shift vector.* We numerically compute the shift vector of the K-valley $A1s$ exciton in a three-band model of MoS_2 using two formulations. (Method 1) The "full" expression [Eq. (7) of the main text], which includes the $\boldsymbol{\kappa}$ derivative and explicit dependence on the light-polarization angle θ, is used to compute the y-direction shift vector. The resulting shift vectors as functions of armchair-direction strain are independent of θ and vanish ($R_y = 0$) in the absence of strain. (Method 2) The same quantity is computed using the Wannier-based formula [Eq. (10) of the main text] involving only Wannier functions and the exciton envelope function, and is manifestly independent of light-polarization. The two methods agree quantitatively: the standard deviations between the Wannier-based and full results for the three different light polarizations are all below $5 \times 10^{-7}a$.

vectors being light-polarization-independent and highlights the computational efficiency of the simplified Wannier based approach (i.e. Method 2). Because the Wannier based shift vector formula Eq. (10) depends only on MLWFs-readily generated by the Wannier90 package-and envelope functions from standard GW-BSE calculations, it is highly efficient and can be readily adapted into standard DFT+GW-BSE workflow in future studies.

Change: A detailed discussion of how this procedure is performed is now described in **SI** Section. X.

2. **Reviewer 2:** I noticed also a typo on the first page-first column: 'posssess'

   Our response: We thank the Reviewer for the careful reading and pointing out the typo. We have corrected the typo, as well as several others we identified, to the best of our knowledge.

Detailed point-by-point response to Reviewer 3

Reviewer 3: I co-reviewed this manuscript with one of the reviewers who provided the listed reports. This is part of the Nature Communications initiative to facilitate training in peer review and to provide appropriate recognition for Early Career Researchers who co-review manuscripts.

Our response: We have addressed all comments from the co-reviewer in our responses above.

Detailed point-by-point response to Reviewer 4

0. **Reviewer 4:** While it is interesting to see the parallel in the mathematical structure between the shift current and earlier ideas about quantum geometry, conceptually, there does not seem to be much that is fundamentally new in this paper. The flux insertion is a standard technique and the derived dependence of the shift vector on polar symmetry is true for any vector and also seen in the symmetry of the shift current conductivity tensor derived in previous work. There does not seem to be a clear measurable prediction that differs from previous work.

   Our Response: We thank the Reviewer for refereeing our manuscript as well as providing comments and questions which have helped us improve our manuscript. We have fully implemented all suggestions by the Reviewer and we address all questions and comments in the detailed point-by-point response below.

   Importantly, we respectfully but strongly disagree with the Reviewer's assessment that "there does not seem to be much that is fundamentally new" in our work. First, we find the dramatic conclusions (e.g., zeroing of shift current in non-polar systems) obtained from our result that the exciton shift vector is light polarization independent cannot be seen from simple symmetry analysis of the shift current conductivity tensor of previous works. Second, we believe our work provides a clear measurable prediction that differs from previous work. We address these explicitly in three parts below:

   (A) To showcase the conceptual novelty of our work and its distinct difference with previous work, we compare with existing exciton shift current studies. We note that there exists explicit formulas for exciton shift currents in the literature, as we discuss presently.

   The following formula (derived by Pedersen in Physical Review B 92.235432 (2015) and later used by Louie's group in PNAS 118(25), e1906938118 (2021) and subsequent publications) is now widely used in the community to compute the exciton shift current:

$$\sigma^{abc}(0;\omega,-\omega) = -\frac{e^3}{8\pi^3 m}\sum_{m,n}\frac{P^a_m Q^b_{mn}[\Omega^c_n]^*}{E_m(E_n-\hbar\omega)} + (\omega\to-\omega)^*. \tag{R14}$$

   Here $P^a_m = \sum_{\mathbf{k}} \psi^{(m)}_{cv}(\mathbf{k}) p^a_{v c\mathbf{k}}$ is the exciton momentum; $Q^b_{mn} = i\sum_{\mathbf{k}} \psi^{(m)*}_{cv}(\mathbf{k})(\psi^{(n)}_{cv}(\mathbf{k}))_{;k^b}$ is the generalized derivative, with $\psi^{(n)}_{cv}(\mathbf{k})_{;k^b} \equiv \partial_{k^b}\psi^{(n)}_{cv}(\mathbf{k}) - i\psi^{(n)}_{cv}(\mathbf{k})[\Omega^b_{cc,\mathbf{k}} - \Omega^b_{vv,\mathbf{k}}]$); $\Omega^c_n = \sum_{\mathbf{k}} \psi^{(n)}_{cv}(\mathbf{k})\Omega^c_{vc}(\mathbf{k})$ is the Berry connection. Note that only in this equation in the reply do we use Ω to denote the Berry conenction quoted verbatim from Pedersen's paper —it is more conventionally denoted as $\mathcal{A}_{nm}(\mathbf{k}) \equiv i\langle u_{n,\mathbf{k}}|\nabla_{\mathbf{k}}|u_{m,\mathbf{k}}\rangle$, which we use in our main text.

   As each of the quantities $P^a_m, Q^b_{mn}, \Omega^c_n$ carries a spatial index that transforms as a vector under spatial symmetries, the resulting rank-3 tensor σ^{abc} is analyzed using tensor transformation rules to derive symmetry constraints. As an illustrative example, let's consider a 2d non-centrosymmetric system with C_{3z} symmetry. **Applying C_{3z} to Eq.** (R14) **reveals that a non-vanishing shift current tensor is allowed.** The action of C_{3z} is to relate different tensor components of the shift current tensor : $\sigma_{xxx} = -\sigma_{yxy} = -\sigma_{yyx} = -\sigma_{xyy}$, and $\sigma_{yyy} = -\sigma_{yxx} = -\sigma_{xxy} = -\sigma_{xyx}$ so that it can be described by two linearly-independent components. Importantly, these components can be non-vanishing.

(B) In stark contrast, our results for the shift vector reveal stronger symmetry constraints for excitons than that obtained from a symmetry analysis of Eq. (R14) above. In particular, we find that that non-degenerate exciton shift vector is forced to **vanish** by C_{3z} symmetry. **Consequently, we find the exciton shift current (and associated shift current tensor) is zero.** This is clearly different from what symmetry analysis would produce from the existing literature [namely Physical Review B 92.235432 (2015) and PNAS 118(25), e1906938118 (2021)]. The reason for this distinction is that Eq. (R14) treats excitons and non-interacting particle-hole pairs on equal footing. Consequently, **tensor symmetry analysis of Eq.** (R14) **alone** (without input of detailed information about the relative coordinate localization of excitons) does not distinguish between these cases. As a result, symmetry analysis of Eq. (R14) cannot differentiate excitons and delocalized electron-hole pairs. Only a full calculation with summation over a complete set of intermediate states is able to reveal a vanishing exciton shift current despite a finite response from delocalized particle–hole pairs—a physically natural scenario that becomes clear in our work.

Our formulation clarifies this distinction by isolating the shift vector as the central quantity. After factorization, the optical transition matrix element remains non-zero, and it is the shift vector that encodes the essential difference between excitons and delocalized particle-hole pairs. This then leads to a clear measurable consequence: for excitons, the shift vector doesn't depend on light polarization and inherits the symmetry of the system, i.e. it points along polar directions in a polar crystal (this strong symmetry constraint was not known prior to our work). In contrast, delocalized particle-hole shift vectors depend sensitively on light polarization, with symmetry constraints on shift current only governed by the full response tensor σ^{abc}. As a result, our formulation and its predictions are distinct from what was known before as described above.

This is important because several recent prominent experimental works have puzzled about the negligible observed excitonic shift current in nonpolar C_{3z} symmetric systems [see e.g, Nature Nanotechnology 18, 36–41 (2023)]. Our work demonstrates that excitonic transition shift vector vanishes in nonpolar systems thereby zeroing its associated excitonic shift current.

(C) Our work has several further consequences for many-body quantum geometry. This is important because there has been very active discussion of quantum geometry of resonant transitions which focused on non-interacting electrons (e.g., Nature Physics 18, 290-295 (2022)). Our work reveals that the geometric structure associated with resonant optical transitions between the ground state and excitons is qualitatively different from that of transitions between the ground state and delocalized particle–hole states. In particular, our work uncovers that the parallel transport of exciton tangent vectors preserves both its norm and angle. As a result, the Hermitian connection acquires a factorized form, ensuring that the exciton shift current does not reverse direction upon changing the light polarization (see full discussion in Reply to Reviewer 4-Point 1 below). None of these properties generally hold for delocalized or non-interacting particle–hole pairs, signaling the fundamental impact of interactions on the many-body geometric structure of excitations.

Change: We have added an extensive discussion showcasing the significance of our findings at the end of the section "Symmetry and intrinsic excitonic transition shift vector". Importantly, our formula establishes stricter symmetry constraints for exciton shift currents compared to that of free particle-hole pairs. We have also added a new section discussing the many-body quantum geometry of excitonic transitions titled *Excitonic vs delocalized particle-hole excitation quantum geometry.*

Reviewer 4: If the authors wish to strengthen the points they are making, they could consider expounding on the following points, which could add to the theoretical novelty of the paper

1. **Reviewer 4:** Flux insertion is typically justified by the idea that you have coupling to a physical gauge field, allowing the integrated phase response to be expressed as a curvature or geometric quantity, such as a Berry curvature or quantum metric. In this case, the flux acts on the relative coordinate of the exciton and the response is a matrix element phase derivative not clearly tied to a curvature. The mathematical analogy seems convincing, but it would be more insightful to explicitly show the relation of the shift vector to an intrinsic differential geometric object (in the same way the Berry curvature is an intrinsically defined curvature tensor). Morimoto and Nagaosa have derived such a mapping for the single particle shift vector but not the exciton

   Our response: We thank the Reviewer for this constructive comment regarding the geometric interpretation of the many-body shift vector. We take this opportunity to clarify how the many-body shift vector fits into the framework of many-body quantum geometry. As the Reviewer has correctly pointed out, Morimoto & Nagaosa pioneered the study of expressing single-particle shift vector via differential geometric object (Science Advances, 2(5), e1501524 (2016)). This line of thought was further explored by many authors; an up-to-date treatment for non-interacting electrons was recently presented by by Ahn, Guo, Nagaosa, & Vishwanath, (Nature Physics 18, 290-295 (2022)), where the central object that governs a range of quantum geometric responses is the Hermitian connection governing parallel transport of the tangent vector of optical transitions. Below we answer the Reviewer's question in two parts revealing new results for the many-body excitonic case:

   (A): Using flux threading, we have now extended the Riemannian geometry framework to many-body resonant transitions. The many-body tangent vector for a resonant optical transition is defined as

   $$\hat{e}_a^{0n}(\boldsymbol{\kappa}) \equiv |\Phi_0(\boldsymbol{\kappa})\rangle\, r_{0n}^a(\boldsymbol{\kappa})\, \langle\Phi_n(\boldsymbol{\kappa})|\,. \tag{R15}$$

   Parallel transport of this tangent vector is captured by the covariant derivative $\nabla_{\boldsymbol{\kappa}}$, defined by projection of its derivative onto the tangent plane:

   $$\nabla_{\boldsymbol{\kappa}}\hat{e}_a^{0n}(\boldsymbol{\kappa}) = P_0\big[\partial_{\boldsymbol{\kappa}}\hat{e}_a^{0n}(\boldsymbol{\kappa})\big]P_n, \tag{R16}$$

   where $\partial_{\boldsymbol{\kappa}}$ is the ordinary derivative and $P_{0/n} = |\Phi_{0/n}\rangle\langle\Phi_{0/n}|$ are projectors.

   By construction, $\nabla_{\boldsymbol{\kappa}}\hat{e}_a^{0n}$ lies in the tangent plane and can thus be expanded in the tangent basis via the Hermitian connection C_{bac}^{0n} associated with parallel transport:

   $$\nabla_c\hat{e}_a^{0n} = \sum_b (C^{0n})^b_{ca}\hat{e}_b^{0n}, \quad C_{bca}^{0n} \equiv \sum_e Q_{be}^{0n}(C^{0n})^e_{ca} = (\hat{e}_b^{0n}, \nabla_{\kappa_c}\hat{e}_a^{0n}), \tag{R17}$$

   where the many-body Hermitian metric $Q_{ba}^{0n} \equiv (\hat{e}_b^{0n}, \hat{e}_a^{0n}) = r_{n0}^b r_{0n}^a$ determines the optical transition rate.

   Resonant optical responses can thus be succinctly represented using this geometric language. For example, the many-body shift current is determined by the Hermitian connection tensor:

   $$j^a_{\text{shift}} = -\frac{2\pi e^3}{\hbar}\sum_n \delta(E_{n0} - \hbar\omega)\,\text{Im}[C_{bab}^{n0}]\,|E^b|^2. \tag{R18}$$

(B): Crucially, we find that, electron-hole interactions can produce fundamentally different Riemannian-geometric structure for exciton bound states as compared to the delocalized free particle–hole pairs. Using Eq. (R16) and (R17), we explicitly evaluate the Riemannian geometry structure for these two cases: (i) delocalized particle-hole pairs, (ii) strongly-bound exciton pairs.

From our shift vector discussion, we find that the covariant derivative (by directly evaluating Eq. (R16)) has the structure:

$$\nabla_c(\hat{e}_a^{0n}) = \overbrace{[D_{\kappa_c}\ln(r_{0n}^a)]\hat{e}_a^{0n}}^{\text{General form for delocalized p-h pairs}} \quad ; \quad \nabla_c(\hat{e}_a^{0n}) = \overbrace{i\mathcal{R}_{0n}^c\hat{e}_a^{0n}}^{\text{Factorized form for exciton bound pairs}} . \tag{R19}$$

where parallel transport of the tangent vector of delocalized e-h pairs is multiplied by a complex coefficient $D_{\kappa_c}\ln(r_{0n}^a)$ (with D the covariant derivative: $D_{\kappa_c}\ln(r_{0n}^a) = \partial_{\kappa_c}\ln(r_{0n}^a) + i(\mathcal{A}_0^c - \mathcal{A}_n^c)$) depending on the direction of the tangent vector (see Nature Physics 18, 290-295 (2022)).

In sharp contrast, we find the tangent vector of the exciton bound pairs is only multiplied by a purely imaginary number (i times the exciton shift vector $\mathcal{R}_{0n}^c$) independent of the tangent vector. **Geometrically, this means that parallel transport of the exciton tangent vector is norm and angle-preserving**. Parallel transport of the delocalized pair tangent vector does **not** have these geometric properties.

This has important implications for the Hermitian connection which is determined by the parallel transport of tangent vector:

$$C_{bca}^{0n} = \overbrace{r_{n0}^b D_{\kappa_c}(r_{0n}^a)}^{\text{General form for delocalized p-h pairs}} \quad ; \quad C_{bca}^{0n} = \overbrace{i\mathcal{R}_{0n}^c r_{n0}^b r_{0n}^a}^{\text{Factorized form for exciton bound pairs}} . \tag{R20}$$

Their distinct forms indicate fundamentally different responses for the two cases. For example, since the exciton shift vector $\mathcal{R}_{0n}$ is independent of light polarization, the polarization dependence of the exciton Hermitian connection arises solely from the optical transition matrix elements. For linearly polarized light along direction b, the linear shift current—determined by the imaginary part of the Hermitian connection—factorizes as $\mathcal{R}_{0n}^c|r_{0n}^b|^2$. Since $|r_{0n}^b|^2$ is positive-definite, the direction of linear shift current remains unchanged when the light polarization is varied. In contrast, the shift vector of delocalized pairs, defined in literature by $\mathcal{R}_{0n}^{c,a} = \mathrm{Im}(C_{aca}^{0n})/Q_{aa}^{0n}$, depends on the light-polarization direction a, and consequently its corresponding shift current can change sign with changing light-polarization direction (cf. Physical Review B 61.5337 (2000), Nature Physics 18, 290-295 (2022)).

Change: We have incorporated the above discussion on many-body quantum geometry into the main text as a new section entitled *Excitonic vs delocalized particle-hole excitation quantum geometry.*

2. **Reviewer 4:** The flux insertion technique is also related to the Laughlin argument in quantum Hall effects. Is there any connection between the unbound electron-hole pairs' spectrum's susceptibility to the inserted flux and, say, how the wave functions of electrons are distorted in the presence of a magnetic field as in integer quantum Hall effect?

   Our response: We thank the Reviewer for the comment. We agree that flux insertion and Laughlin's argument in quantum Hall effects are related. For clarity, we answer in three parts:

   (A): Laughlin's argument considers threading a flux in the hole of the cylindrical geometry and examining the wavefunction distortion in response to the flux threading. Laughlin

pointed out (Physical Review B 23.5632 (1981)): "the quantum Hall effect is intimately related to the extended nature of the states near the center of the disorder-broadened Landau level". Even in the presence of disorder, such delocalized bulk states persist. Because of such delocalized states, flux insertion strongly distorts electron wavefunctions allowing "spectral flow": pumping electrons between the edges as flux is inserted.

(B): Yes, the same intuition applies to unbound electron–hole *pair* spectrum under flux insertion. Owing to the strong distortion of their wave functions, the energy spectra of delocalized electron–hole pairs are highly susceptible to flux insertion rapidly changing as flux is inserted (see e.g., explicit example in Fig. 2a and inset of the main text). Similar to Laughlin's "spectral flow" argument, such sensitivity of the unbound electron-hole pair spectrum is a signature of their delocalized nature. Beyond the energy spectrum, our work introduces the Wilson-loop phase $\mathcal{W}$ (a geometric phase accrued associated with photoexcitation) as a powerful tool for analyzing the optical properties of electron–hole pairs. Similar to the unbound electron-hole spectrum, $\mathcal{W}$ also changes rapidly with flux insertion due to the delocalized nature of the unbound electron-hole pair. As explained in the main text, such strong sensitivity of Wilson loop $\mathcal{W}$ to flux insertion for delocalized electron-hole pairs (Fig.2b lower panel) indicates that their shift vector strongly depends on light polarizations.

(C): Importantly, this intuition can be used to directly characterize bound exciton states. For example, owing to their bound nature, exciton energy spectra are exponentially insensitive to flux insertion (see Fig. 2a in the main text). Critically, this means that excitonic Wilson loop phase $\mathcal{W}$ is also exponentially insensitive to flux insertion (see Fig. 2b in the main text). This latter property directly leads to the insensitivity of their shift vectors to light polarizations, showcasing the crucial difference between bound exciton states and delocalized particle-hole pairs in their optical response.

3. **Reviewer 4:** It is not really clear how Eq. 7 follows directly from the decay property in Eq. 4. The argument appears to assume that the off-diagonal elements of the BSE kernel decay exponentially and that the exciton envelope is sufficiently localized, but this connection is not rigorously demonstrated in the main text. It would help if the authors explicitly showed how the localization of the exciton wavefunction quantitatively ensures the flux-independence of the overlap.

   Our response: We appreciate the Reviewer's comment on improving the clarity of our paper. In our previous version, we provided details of this derivation in the supplementary information. However, we agree that it may be better to show this in the main text (see revised page 3). We have now moved this discussion into the main text and sharpened the logical flow. For the convenience of the Reviewer, we sketch the essential steps below.

   Our key claim is that electric polarization changes from an insulating many-body ground state to an excited excitonic state is *insensitive* to the form of $\hat{V}$, where $\hat{V}$ is the light-matter interaction. To see this we consider two light matter interactions $\hat{V}_1$ and $\hat{V}_2$ e.g., from two different light polarizations; both $\hat{V}_1$ and $\hat{V}_2$ produce an excitation from the ground state to exciton state. We write the difference between their light induced polarization changes as $\delta\boldsymbol{\mathcal{R}} = \boldsymbol{\mathcal{R}}^{V_1}_{0\to\text{ex}} - \boldsymbol{\mathcal{R}}^{V_2}_{0\to\text{ex}} = \nabla_{\boldsymbol{\kappa}}\text{arg}\mathcal{W}_{\boldsymbol{\kappa}}$, where the Wilson loop $\mathcal{W}_{\boldsymbol{\kappa}} = [V_1^{\boldsymbol{\kappa}}]_{0\to\text{ex}}[V_2^{\boldsymbol{\kappa}}]_{\text{ex}\to 0}$, with a general transition matrix element represented by $[V^{\boldsymbol{\kappa}}]_{n\to m} = \langle\Phi_n(\boldsymbol{\kappa})|\,\hat{V}(\boldsymbol{\kappa})\,|\Phi_m(\boldsymbol{\kappa})\rangle$. Note that the transition matrix element between the ground state and the exciton state can be directly evaluated via Eq. (1) of the main text:

$$[V^{\boldsymbol{\kappa}}]_{0\to\text{ex}} = \sum_{\mathbf{R}_e,\mathbf{R}_h} \frac{e^{i\boldsymbol{Q}\cdot\mathbf{R}_{\text{cm}}}}{\sqrt{N}} v^{\boldsymbol{\kappa}}_{\mathbf{R}_h,\mathbf{R}_e}\psi^{\boldsymbol{\kappa}}_{\boldsymbol{Q}}(\mathbf{R}_e - \mathbf{R}_h), \tag{R21}$$

where $v^{\boldsymbol{\kappa}}_{\mathbf{R}_h,\mathbf{R}_e} = \langle w^{\boldsymbol{\kappa}}_{\mathbf{R}_h} | \hat{V} | w^{\boldsymbol{\kappa}}_{\mathbf{R}_e} \rangle$ is the transition amplitude in the Wannier basis and $\mathbf{R}_{\rm cm} = (\mathbf{R}_e + \mathbf{R}_h)/2$ is the center-of-mass coordinate. To analyze Eq. (R21), we first note that the flux-inserted transition amplitude is related to that without flux via the relation: $v^{\boldsymbol{\kappa}}_{\mathbf{R}_h,\mathbf{R}_e} = e^{-i\boldsymbol{\kappa}(\mathbf{R}_h - \mathbf{R}_e)} v_{\mathbf{R}_h,\mathbf{R}_e}$, which originates from the Wannier function transformation rule $w^{\boldsymbol{\kappa}}_{\boldsymbol{R}}(\mathbf{x}) = e^{-i\boldsymbol{\kappa}\cdot(\mathbf{x}-\boldsymbol{R})} w_{\boldsymbol{R}}(\mathbf{x})$. Secondly, the flux-inserted envelope function $\psi^{\boldsymbol{\kappa}}_{\boldsymbol{Q}}(\mathbf{R}_e - \mathbf{R}_h)$ picks up a phase factor $e^{-i\boldsymbol{\kappa}\cdot(\mathbf{R}_e - \mathbf{R}_h)}$ according to $\psi^{\boldsymbol{\kappa}}_{\boldsymbol{Q}}(\mathbf{r}) = e^{-i\boldsymbol{\kappa}\cdot\mathbf{r}} \psi_{\boldsymbol{Q}}(\mathbf{r}) + \mathcal{O}(e^{-L/(2\xi_M)})$ in the main text. Combining these, we find that the $\boldsymbol{\kappa}$-dependent phases compensate in the transition matrix element Eq. (R21), resulting in the $\boldsymbol{\kappa}$ insensitivity of the full Wilson loop operator and leads to the following inequality:

$$|\delta \boldsymbol{\mathcal{R}}| = |\nabla_{\boldsymbol{\kappa}} \arg \mathcal{W}_{\boldsymbol{\kappa}}| < \mathcal{D} e^{-L/\xi_M}, \tag{R22}$$

where $\mathcal{D}$ is a constant independent of L.

Change: We have added the above discussion right before we introduce the inequality Eq.(9) in the main text.

4. **Reviewer 4:** The numerical calculations also appear somewhat insufficient to support all the claims in the paper. It would be valuable to show the shift vector with and without electron-hole interactions to explicitly demonstrate the role of excitons relative to the independent particle picture. Also, it would be helpful if the shift current conductivity tensor could be calculated in addition to just the shift vector for a clearer connection to physical observables.

   Our response: We thank the Reviewer for the comment which we have fully implemented (see points A and B below). While we believe our central claims (e.g., exciton shift vector is independent of light polarization) have been rigorously proven analytically in the large system size limit (see e.g., Eq. (9) and (10) in the main text), we nevertheless appreciate the Reviewer's suggestion to illustrate our results in a clearer figures more easily parsed by a broad audience.

   To this end, we performed numerical simulations on a tight-binding model of MoS_2, with parameters all obtained from density functional theory calculations of MoS_2 (cf. Phys Rev B, 88, 085433 (2013)). A strain is incorporated along the armchair direction to reveal its effects on the shift-current response. These are now plotted as a new Figure 3 in the revised main text and discussed on page 7. For the convenience of the reviewer, we also include them here and discuss them briefly. The results are two fold:

   (A): Following the Reviewer's first suggestion, we computed the shift vectors for both bound exciton states and non-interacting particle-hole pairs. As shown in Fig. R4(c) (Fig. 3c of the revised main text), the non-interacting particle-hole shift vectors were evaluated at K point for different light polarizations and at varying strain. They exhibit a strong light-polarization dependence. Importantly, notice even in the unstrained case, the shift vectors at these C_{3z} symmetric momenta are non-zero and vary significantly with light polarization as shown in Fig. R4(c) inset (Fig. 3c inset in the main text). By contrast, we also computed the A1s exciton shift vector at K valley. A direct evaluation of the shift-vector formula (Eq.7) shows that exciton shift vector is completely independent of light-polarization (Fig. R4(d), Fig. 3d in the main text), and moreover vanishes in the unstrained case due to C_{3z} rotation symmetry, in full agreement with our theoretical analysis.

   (B): Per the Reviewer's second suggestion, we have also computed the shift currents under different light polarizations. A salient feature of the free particle-hole shift current is its strong light-polarization dependence, which enables the shift current to change sign as light polarization varies, as shown in Fig. R4(e) (Fig.3e in the main text). In stark

contrast, the exciton shift current is obtained by multiplying the polarization-independent exciton shift vector by the squared optical matrix element, which is positive definite and only weakly polarization-dependent. Consequently, the exciton shift current does not change sign and exhibits only a weak dependence on on light polarization, as shown in Fig.R4(f) for the convenience of the Reviewer (Fig.3f in the main text).

Change: We have incorporated the above discussion into the section *Excitonic transition shift vector in* MoS_2 (and correspondingly Fig.3 in the main text), where we present a numerical simulation for MoS_2.

5. **Reviewer 4:** It would be greatly helpful if the authors can provide a numerical example of how the shift vector does not transform as a vector when the exciton is not strongly bound.

Our response: We thank the Reviewer for the useful suggestion which we fully implement below. Key to our revision was providing clarity in what we mean by "transform as a vector" and "does not transform as a vector". We provide a detailed discussion as well as a numerical example (per Reviewer suggestion) in the revised text. We summarized this below in two parts:

(A): First, we clarify what we mean by "transform as a vector". When we say that a physical object $\mathbf{R}$ transforms as a vector, we mean that it carries one spatial index, and under point-group operation g, its components transform as: $R^a \to \sum_b [U_g]^{ab} R^b$, where U_g is the matrix representation of g acting on spatial coordinates. If the system is symmetric under g, then any physical vector quantity $\mathbf{R}$ of this system must remain invariant under the symmetry operation g. Therefore, it must satisfy

$$R^a = \sum_b [U_g]^{ab} R^b \tag{R23}$$

for every symmetry element g. Such conditions restrict the possible non-zero components of $\mathbf{R}$. When an observable satisfies Eq. (R23), we say it "transforms as a vector".

In the case of excitons, we have derived the exciton shift vector relation under point group symmetry g for non-degenerate excitons:

$$\boldsymbol{\mathcal{R}}_{0\to\mathrm{ex}}(\hat{U}_g \boldsymbol{Q}) = \hat{U}_g[\boldsymbol{\mathcal{R}}_{0\to\mathrm{ex}}(\boldsymbol{Q})]. \tag{R24}$$

where $\boldsymbol{Q}$ is the center-of-mass momentum. According to the criteria laid out in Eq.(R24) above, we conclude that $\boldsymbol{\mathcal{R}}_{0\to\mathrm{ex}}(\boldsymbol{Q}=0)$ **transforms as a vector when** $\boldsymbol{Q}=\mathbf{0}$ (vertical optical transition). From this constraint, one can directly determine the non-zero components and directions that the exciton shift vector $\boldsymbol{\mathcal{R}}$ may take. In particular, this means that $\boldsymbol{\mathcal{R}}_{0\to\mathrm{ex}}(\boldsymbol{Q}=0)$ lies along the polar axes of a material; it vanishes in non-polar materials as discussed in the main text. For example, for a C_{3z} symmetric 2D material, the exciton shift vector *vanishes.*

(B): By delocalized p-h pair shift vector "does not transform as a vector", we mean that symmetry operations does not constrain their shift vector at a fixed light polarization, i.e. it does not satisfy Eq. (R23) for a fixed light polarization. The reason is that delocalized p-h pair shift vectors depend explicitly on the light polarization, and symmetry operations only relate shift vectors evaluated at *different* polarizations.

To illustrate this, consider free particle-hole pairs in MoS_2. The band extrema are located at the K and K' points in the Brillouin zone, both of which are invariant under C_{3z} rotations. Focusing on an excitation at the K valley, the delocalized p-h pair shift vector transforms under C_{3z} symmetry as

$$\boldsymbol{R}_K(\hat{e}_\theta) = \hat{U}_{C_{3z}}\left[\boldsymbol{R}_K(\hat{e}_{\theta+2\pi/3})\right], \tag{R25}$$

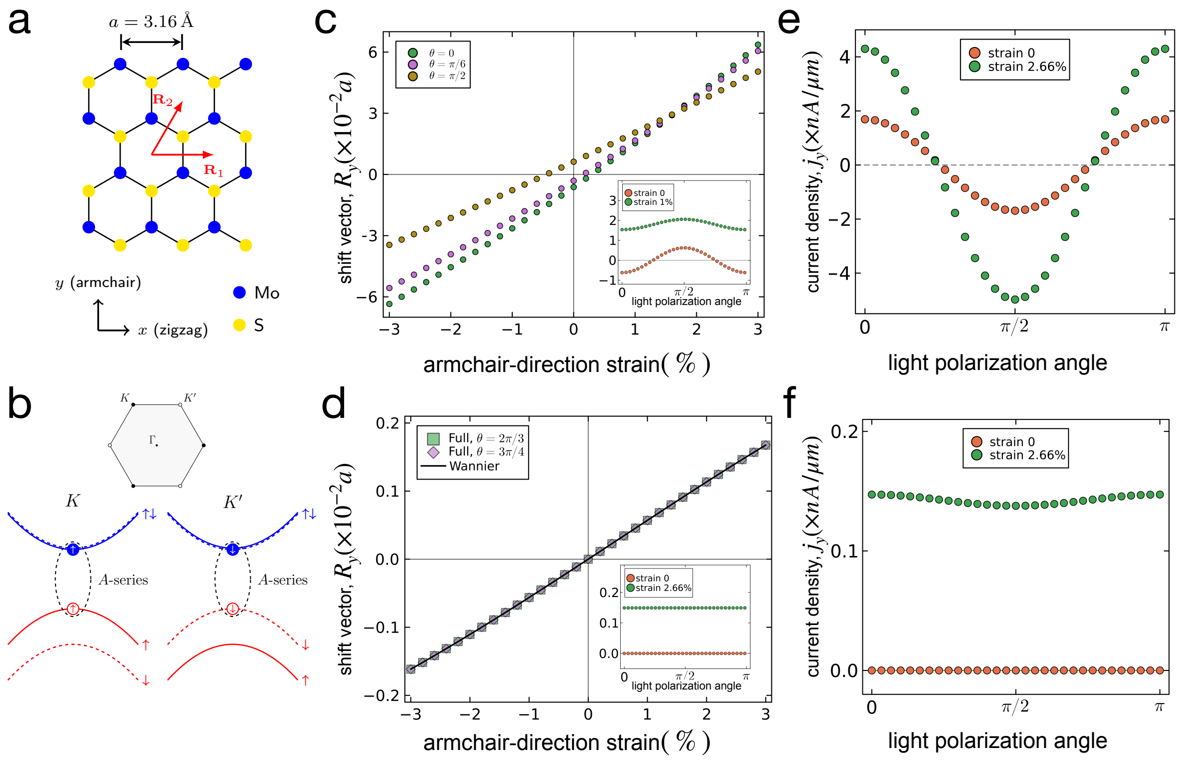

Figure R4: *Numerical calculation of shift vector and shift current in a three-band tight-binding model of MoS_2.* (a) Schematic of the monolayer MoS_2 lattice; arm chair direction is along y. (b) A-series excitons at the K, K' valleys in the Brillouin zone. Each exciton is two-fold valley-degenerate. Strong spin–orbit coupling splits the valence bands (with smaller conduction-band splittings) and produces spin-valley locking: K-valley A-excitons involve up-spin electron-hole pairs, while K'-valley excitons involve down-spin pairs. (c) Strain dependence of free particle-hole pair shift vectors for direct optical transitions at sampled momenta, which remain non-zero in the unstrained case and depend strongly on light-polarization angle θ (inset: polarization dependence of shift vector at K for the unstrained and 1% strained case). (d) Strain dependence of the A1s exciton shift vector at K composed of spin-up particle-hole pairs, which are polarization independent and vanish in the unstrained limit. Dots are computed via the full formula (Eq. (7) in the main text) and line is computed via the Wannier-based formula (Eq. (10) in the main text); both agree with each other. (inset: polarization dependence of A1s exciton shift vector for the unstrained and 2.66% strained ($\delta t/t = -0.1$) case via full formula). (e) Shift current for *free particle-hole pairs* at a photon energy of $\hbar\omega = 2.5$eV, for both zero strain and 2.66% strain ($\delta t/t = -0.1$). Strong polarization-dependent shift vectors cause the current to *reverse sign* as the polarization direction changes. (f) Shift current for A_{1s} *excitons* at photon energy $\hbar\omega = 1.08$eV with the same strains (e). Since the exciton shift vector is polarization-independent, the resulting excitonic transition shift current's weak polarization dependence comes from the squared optical matrix element and does not change sign.

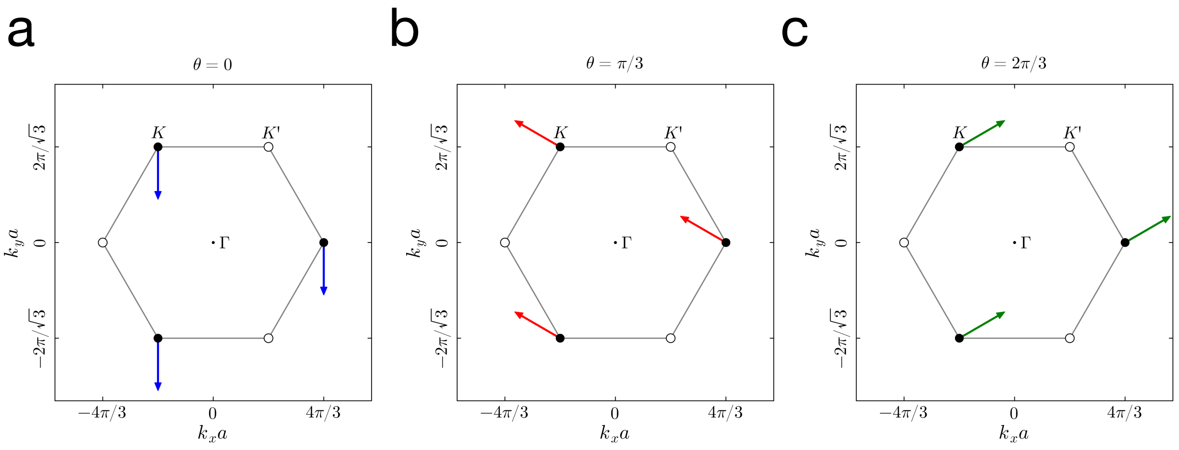

Figure R5: *Free particle-hole pair shift vector in a three-band tight-binding model of unstrained* MoS_2. (a) Shift vectors evaluated at the three C_{3z}-equivalent K points for light polarization $\theta = 0$. At this polarization, the shift vector does not transform as a vector under C_{3z} symmetry and is therefore not constrained to vanish. (b) Same setup as in (a), but with the light-polarization angle $\theta = \pi/3$. In this case, shift vectors are related to those at $\theta = 0$ by $\boldsymbol{R}_K(\hat{e}_{\theta=\pi/3}) = [\hat{U}_{C_{3z}}]^2 \boldsymbol{R}_K(\hat{e}_{\theta=0})$. (c) Same setup as in (a), but with the light-polarization angle $\theta = 2\pi/3$. The shift vectors are related to those at $\theta = 0$ by $\boldsymbol{R}_K(\hat{e}_{\theta=2\pi/3}) = \hat{U}_{C_{3z}} \boldsymbol{R}_K(\hat{e}_{\theta=0})$.

This relation shows that the symmetry operation acts simultaneously on the spatial components of the shift vector *and* on the light polarization.

Thus, in this context, "does not transform as a vector" means that the shift vector evaluated at a fixed light polarization angle θ is not constrained by C_{3z} symmetry. In particular,

$$\boldsymbol{R}_K(\hat{e}_\theta) \neq \hat{U}_{C_{3z}} \big[\boldsymbol{R}_K(\hat{e}_\theta)\big], \tag{R26}$$

so that the shift vector of a delocalized particle-hole pair does not satisfy the vector constraint Eq. (R23). As a result, and as explicitly shown in Fig. R5 (found in Sec. XI of revised SI), the shift vector of a delocalized particle-hole pair at fixed light polarization is generically nonzero, leading to a finite shift current from delocalized p-h excitations. This finite response is probed by photons with energies just above the band gap. This behavior stands in stark contrast to the exciton case, where C_{3z} symmetry enforces a vanishing exciton shift vector at fixed light polarization and, consequently, an identically zero exciton shift current.

Change: We have clarified what we mean by "transforming as a vector" in the last paragraph of page 4 and have included the numerical example in **SI**, Sec. XI.

Detailed point-by-point response to Reviewer 5

Reviewer 5: I co-reviewed this manuscript with one of the reviewers who provided the listed reports. This is part of the Nature Communications initiative to facilitate training in peer review and to provide appropriate recognition for Early Career Researchers who co-review manuscripts.

Our response: We have addressed all comments from the co-reviewer in our responses above.
